# Supplementary material for: Anti-leishmanial physalins—Phytochemical investigation, in vitro evaluation against clinical and MIL-resistant L. tropica strains and in silico studies
Source: PLoS One. 2022 Nov 28;17(11):e0274543. doi: 10.1371/journal.pone.0274543 (PMC9704608; doi:10.1371/journal.pone.0274543)
Supplement: S1 File — (PDF) [file pone.0274543.s026.pdf]

Window Display Report

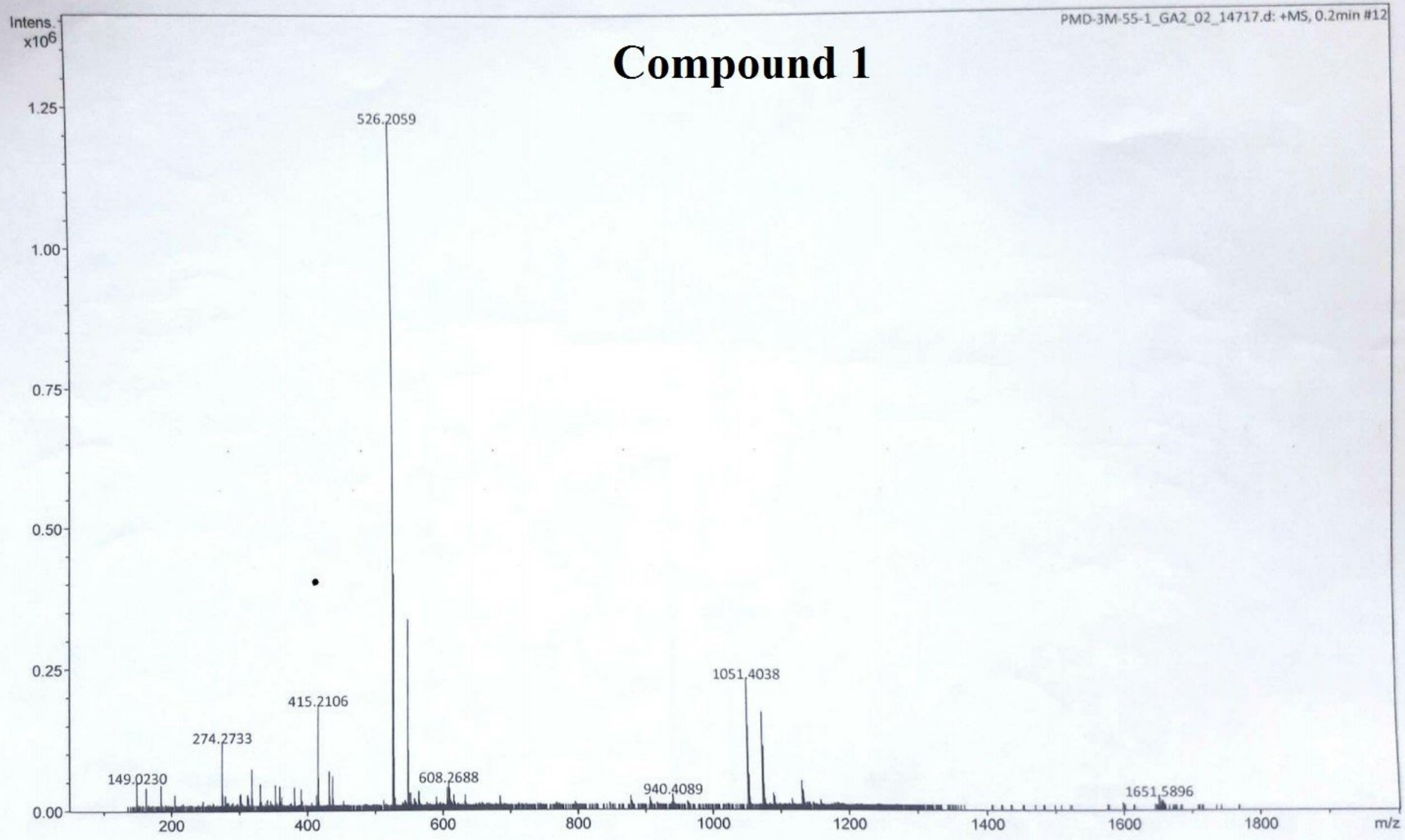

Bruker Compass DataAnalysis 4.2 printed: 11/24/2020 9:52:48 AM by: ARSALAN/JUNAID

CS CamScanner

Compound 1

Lower formula: C_{23}

Upper formula: $C_{28}H_{35}$

C 23-28, H 0-35

Note: for m < 2000 the elements C, H, N, and O are considered implicitly.

Adducts, pos. M+H ☐ Collect adducts

Adducts, neg. M-H

Measured m/z 526.2059 Tolerance: 5 ppm Charge: 1

Meas. m/z	#	Ion Formula	m/z	err [ppm]	mSigma	# mSigma	Score	rdb	e⁻ Conf	N-Rule
526.2059	1	C28H32NO9	526.2072	2.3	11.4	1	83.37	13.5	even	ok
526.2059	2	C25H24N11O3	526.2058	-0.3	15.7	2	100.00	19.5	even	ok
526.2059	3	C24H28N7O7	526.2045	-2.8	23.8	3	54.70	14.5	even	ok

☐ Automatically locate monoisotopic peak Maximum number of formulae 500

☑ Check rings plus double bonds Minimum -0.5 Maximum 40

Electron configuration even

☑ Filter H/C element ratio Minimum H/C: 0 Maximum H/C: 3

☑ Estimate carbon number ☑ Generate immediately

Copy to SmartFormula Parameters Show Pattern Generate Help

CS CamScanner

Compound 1

DR.SAIRA BANO/DR.IQBAL/PMD-3M-55-IR/CDCL3
1H

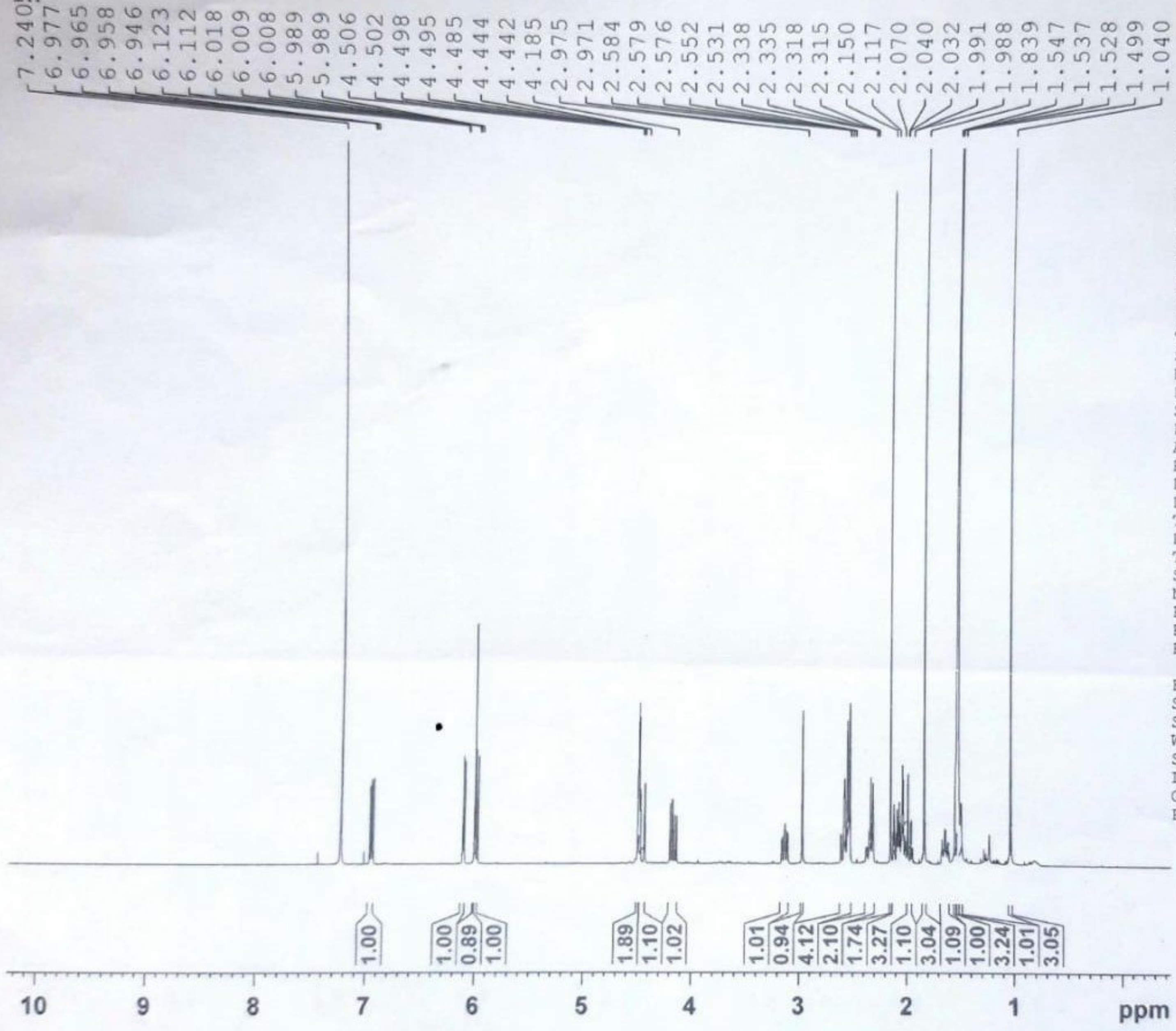

AVANCE NEO
500 MHz
LAB # 109-B

Current Data Parameters	
NAME	oct16-20
EXPNO	2
PROCNO	1

F2 - Acquisition Parameters	
Date_	20201016
Time	13.17 h
INSTRUM	AVNeo500MHz
PROBHD	Z859201_0007 (
PULPROG	zg30
TD	65536
SOLVENT	CDCl3
NS	128
DS	0
SWH	10000.000 Hz
FIDRES	0.305176 Hz
AQ	3.2767999 sec
RG	101
DW	50.000 usec
DE	11.31 usec
TE	300.0 K
D1	1.50000000 sec
TD0	1
SFO1	500.1340010 MHz
NUC1	1H
P0	2.33 usec
P1	7.00 usec
PLW1	17.96400070 W

F2 - Processing parameters	
SI	32768
SF	500.1300220 MHz
WDW	EM
SSB	0
LB	0.30 Hz
GB	0
PC	1.00

CS CamScanner

Dr.Saira / Dr.Iqbal / PMD-3M-55-1 / CDCL3
BB

Compound 1

AVANCE NEO
600 MHz (LC)
Cryoprobe
Lab # 108

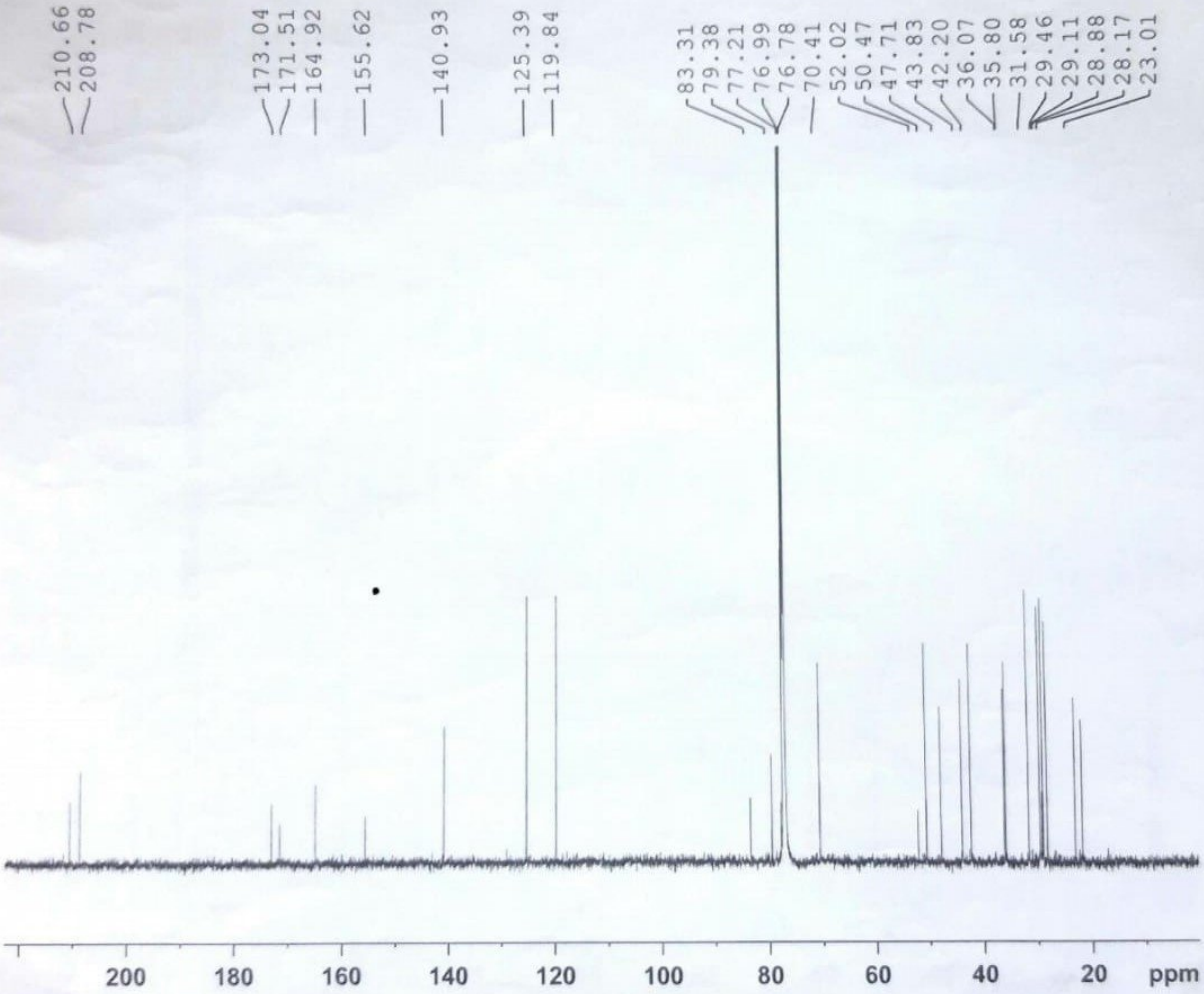


```
Current Data Parameters
NAME            Sep23-20
EXPNO                  8
PROCNO                 1

F2 - Acquisition Parameters
Date_           20200924
Time             20.26 h
INSTRUM  Avance NEO 600MHz
PROBHD   Z44896_0021 (C
PULPROG             zgpg
TD                 32768
SOLVENT            CDCl3
NS                 16384
DS                     8
SWH           35714.285 Hz
FIDRES         2.179827 Hz
AQ            0.4587520 sec
RG                   101
DW               14.000 usec
DE                18.00 usec
TE                298.0 K
D1           2.00000000 sec
D11          0.03000000 sec
TD0                   16
SFO1         150.8950144 MHz
NUC1                 13C
P1                12.00 usec
PLW1         97.90100098 W
SFO2         600.0324001 MHz
NUC2                  1H
CPDPRG[2         waltz65
PCPD2             75.00 usec
PLW2          4.50000000 W
PLW12         0.08000000 W
PLW13         0.04619300 W

F2 - Processing parameters
SI                 16384
SF           150.8776660 MHz
WDW                   EM
SSB      0
LB                  1.00 Hz
GB       0
PC                  0.30
```

CS CamScanner

Dr.Saira / Dr.Iqbal / PMD-3M-55-1 / CDCL3
DEPT135

Compound 1

AVANCE NEO
600 MHz (LC)
Cryoprobe
Lab # 108

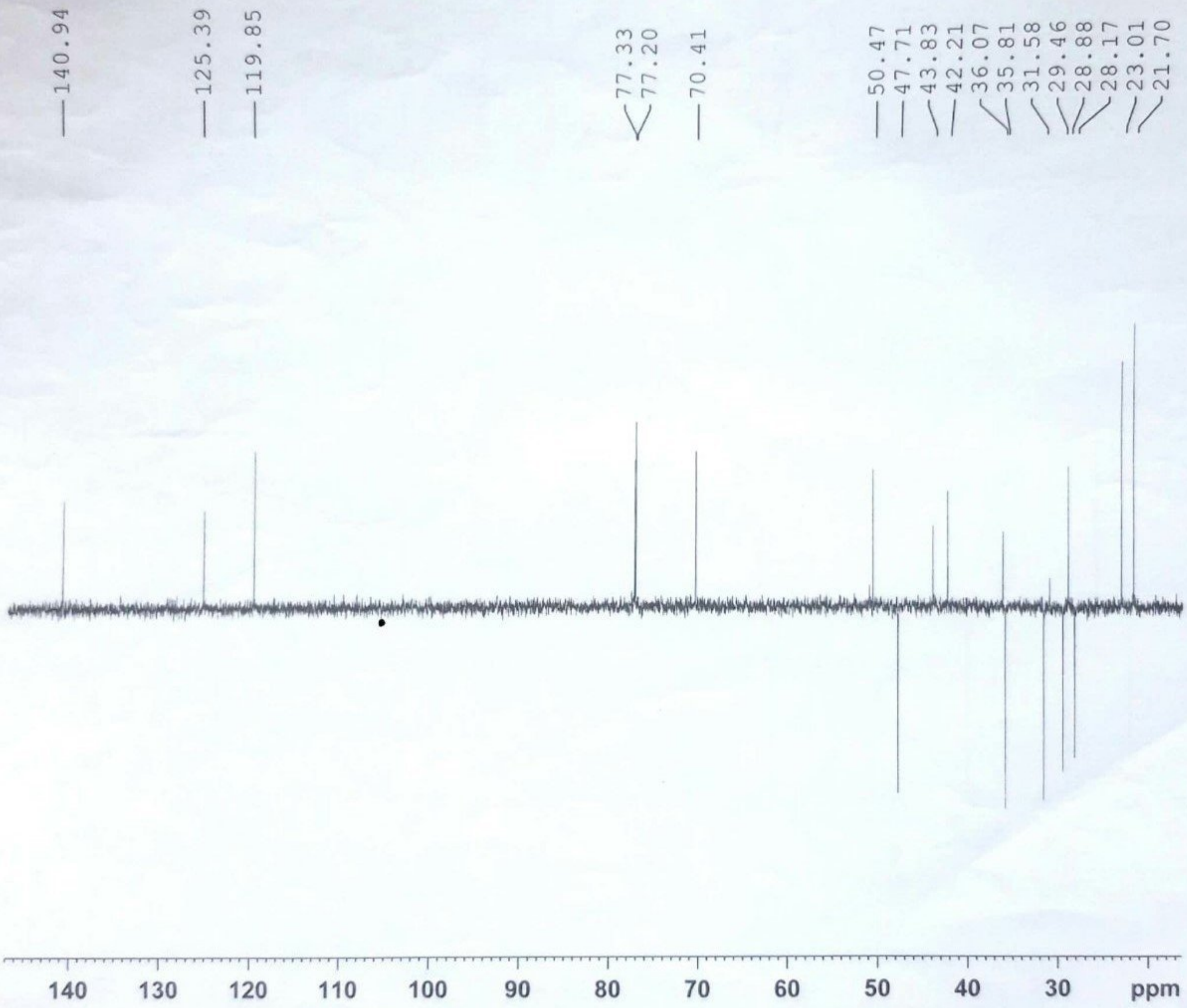

Current Data Parameters
NAME Sep23-20
EXPNO 9
PROCNO 1

F2 - Acquisition Parameters
Date_ 20200925
Time 1.11 h
INSTRUM Avance NEO 600MHz
PROBHD Z44896_0021 (C
PULPROG deptsp135
TD 32768
SOLVENT CDCl3
NS 8192
DS 8
SWH 30120.482 Hz
FIDRES 1.838408 Hz
AQ 0.5439488 sec
RG 101
DW 16.600 usec
DE 18.00 usec
TE 298.0 K
CNST2 145.0000000
D1 1.50000000 sec
D2 0.00344828 sec
D12 0.00002000 sec
TD0 8
SFO1 150.8919969 MHz
NUC1 13C
P1 12.00 usec
P13 2000.00 usec
PLW0 0 W
PLW1 97.90100098 W
SPNAM[5] Crp60comp.4
SPOAL5 0.500
SPOFFS5 0 Hz
SPW5 21.54000092 W
SFO2 600.0324001 MHz
NUC2 1H
CPDPRG[2 waltz65
P3 10.00 usec
P4 20.00 usec
PCPD2 75.00 usec
PLW2 4.50000000 W
PLW12 0.08000000 W

F2 - Processing parameters
SI 16384
SF 150.8776660 MHz
WDW EM
SSB 0
LB 1.00 Hz
GB 0
PC 1.20

CS CamScanner

Compound 1

Dr.Saira / Dr.Iqbal / PMD-3M-55-1 / CDCL3
Dept90

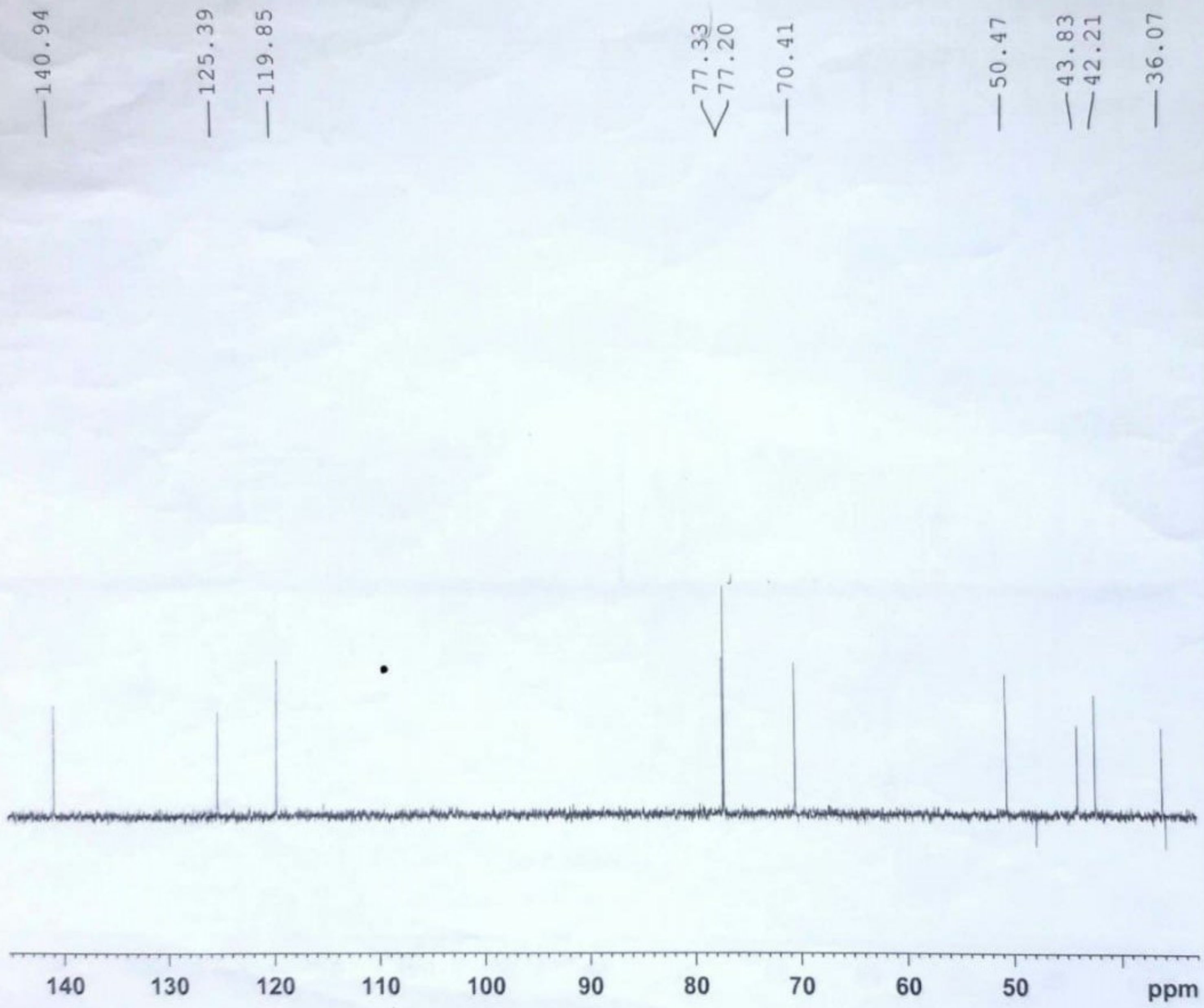

AVANCE NEO
600 MHz (LC)
Cryoprobe
Lab # 108

Current Data Parameters

NAME	Sep23-20
EXPNO	10
PROCNO	1

F2 - Acquisition Parameters

Date_	20200925
Time	3.34 h
INSTRUM	Avance NEO 600MHz
PROBHD	Z44896_0021 (C
PULPROG	deptsp90
TD	32768
SOLVENT	CDCl3
NS	4096
DS	8
SWH	30120.482 Hz
FIDRES	1.838408 Hz
AQ	0.5439488 sec
RG	101
DW	16.600 usec
DE	18.00 usec
TE	298.0 K
CNST2	145.0000000
D1	1.50000000 sec
D2	0.00344828 sec
D12	0.00002000 sec
TD0	4
SFO1	150.8919969 MHz
NUC1	13C
P1	12.00 usec
P13	2000.00 usec
PLW0	0 W
PLW1	97.90100098 W
SPNAM[5]	Crp60comp.4
SPOAL5	0.500
SPOFFS5	0 Hz
SPW5	21.54000092 W
SFO2	600.0324001 MHz
NUC2	1H
CPDPRG[2	waltz65
P3	10.00 usec
P4	20.00 usec
PCPD2	75.00 usec
PLW2	4.50000000 W
PLW12	0.08000000 W

F2 - Processing parameters

SI	16384
SF	150.8776660 MHz
WDW	EM
SSB	0
LB	1.00 Hz
GB	0
PC	0.80

CS CamScanner

Compound 1

Dr.Saira / Dr.Iqbal / PMD-3M-55-1 / CDCL3
Cosy

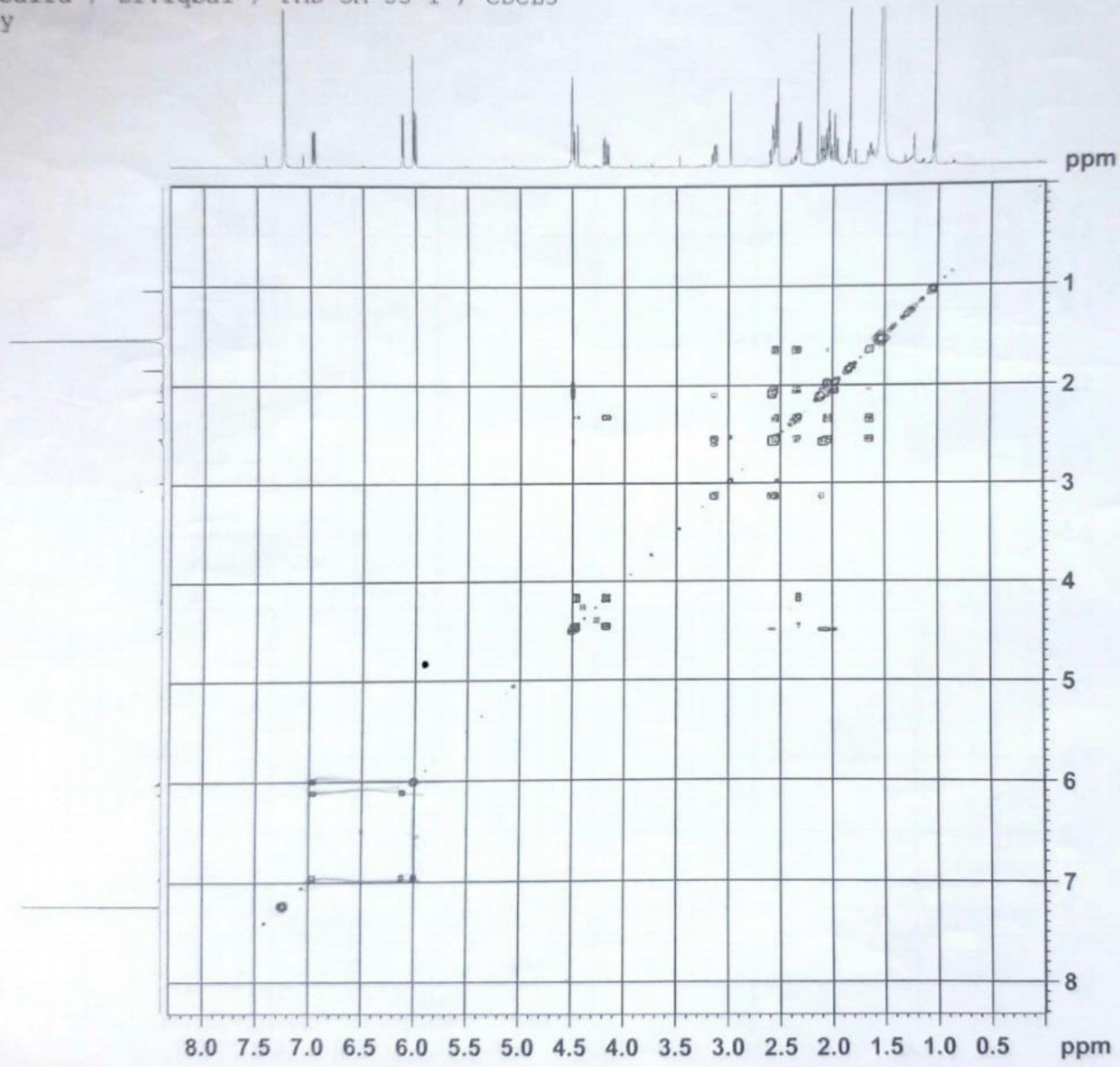

AVANCE NEO
600 MHz (LC)
Cryoprobe
Lab # 108

Current Data Parameters	
NAME	Sep23-20
EXPNO	4
PROCNO	1
F2 - Acquisition Parameters	
Date_	20200923
Time	14.30 h
INSTRUM	Avance NEO 600MHz
PROBHD	244896_0021 (C
PULPROG	cosygpqf
TD	2048
SOLVENT	CDCl3
NS	8
DS	16
SWH	5000.000 Hz
FIDRES	4.882813 Hz
AQ	0.2048000 sec
RG	101
DW	100.000 usec
DE	15.00 usec
TE	298.0 K
D0	0.00000300 sec
D1	2.00000000 sec
D13	0.00000400 sec
D16	0.00020000 sec
IN0	0.00020000 sec
TDav	1
SFO1	600.0325201 MHz
NUC1	1H
P0	10.00 usec
P1	10.00 usec
PLW1	4.25000000 W
GPNAM[1]	SMSQ10.100
GPZ1	10.00 %
P16	1000.00 usec
F1 - Acquisition parameters	
TD	256
SFO1	600.0325 MHz
FIDRES	39.062500 Hz
SW	8.333 ppm
FnMODE	QF
F2 - Processing parameters	
SI	1024
SF	600.0300266 MHz
WDW	QSINE
SSB	0
LB	0 Hz
GB	0
PC	1.00
F1 - Processing parameters	
SI	1024
MC2	QF
SF	600.0300266 MHz
WDW	QSINE
SSB	0
LB	0 Hz
GB	0

CS CamScanner

Compound 1

Dr.Saira / Dr.Iqbal / PMD-3M-55-1 / CDCL3
Noesy

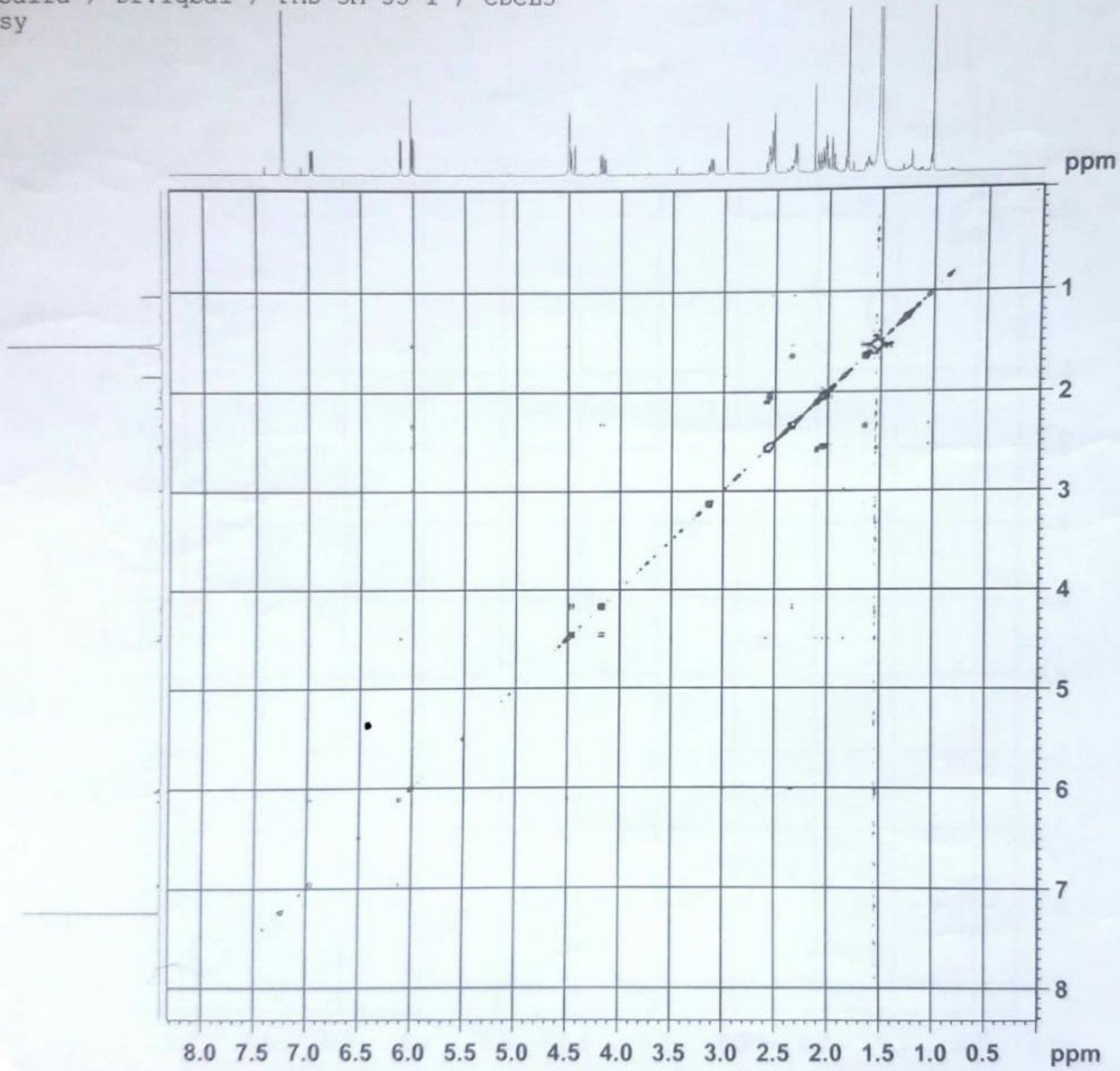

AVANCE NEO
600 MHz (LC)
Cryoprobe
Lab # 108

```
Current Data Parameters
NAME          Sep23-20
EXPNO                5
PROCNO               1

F2 - Acquisition Parameters
Date_           20200923
Time             20.15 h
INSTRUM   Avance NEO 600MHz
PROBHD    Z44896_0021 (C
PULPROG       noesygpph
TD                 2048
SOLVENT           CDCl3
NS                   32
DS                   16
SWH          5000.000 Hz
FIDRES       4.882813 Hz
AQ          0.2048000 sec
RG              67.6187
DW          100.000 usec
DE            15.00 usec
TE              298.0 K
D0       0.00008727 sec
D1       1.50000000 sec
D8       0.80000001 sec
D16      0.00020000 sec
IN0      0.00020000 sec
TDav                  1
SFO1     600.0325201 MHz
NUC1                 1H
P1            10.00 usec
P2            20.00 usec
PLW1       4.25000000 W
GPNAM[1]     SMSQ10.100
GPZ1            40.00 %
P16         1000.00 usec

F1 - Acquisition parameters
TD                  256
SFO1        600.0325 MHz
FIDRES     39.062500 Hz
SW             8.333 ppm
FnMODE      States-TPPI

F2 - Processing parameters
SI                 1024
SF       600.0300266 MHz
WDW               QSINE
SSB                   2
LB          0 Hz
GB          0
PC                 1.00

F1 - Processing parameters
SI                 1024
MC2         States-TPPI
SF       600.0300266 MHz
WDW               QSINE
SSB                   2
LB          0 Hz
GB          0
```

CS CamScanner

Compound 1

Dr.Saira / Dr.Iqbal / PMD-3M-55-1 / CDCL3
Noesy

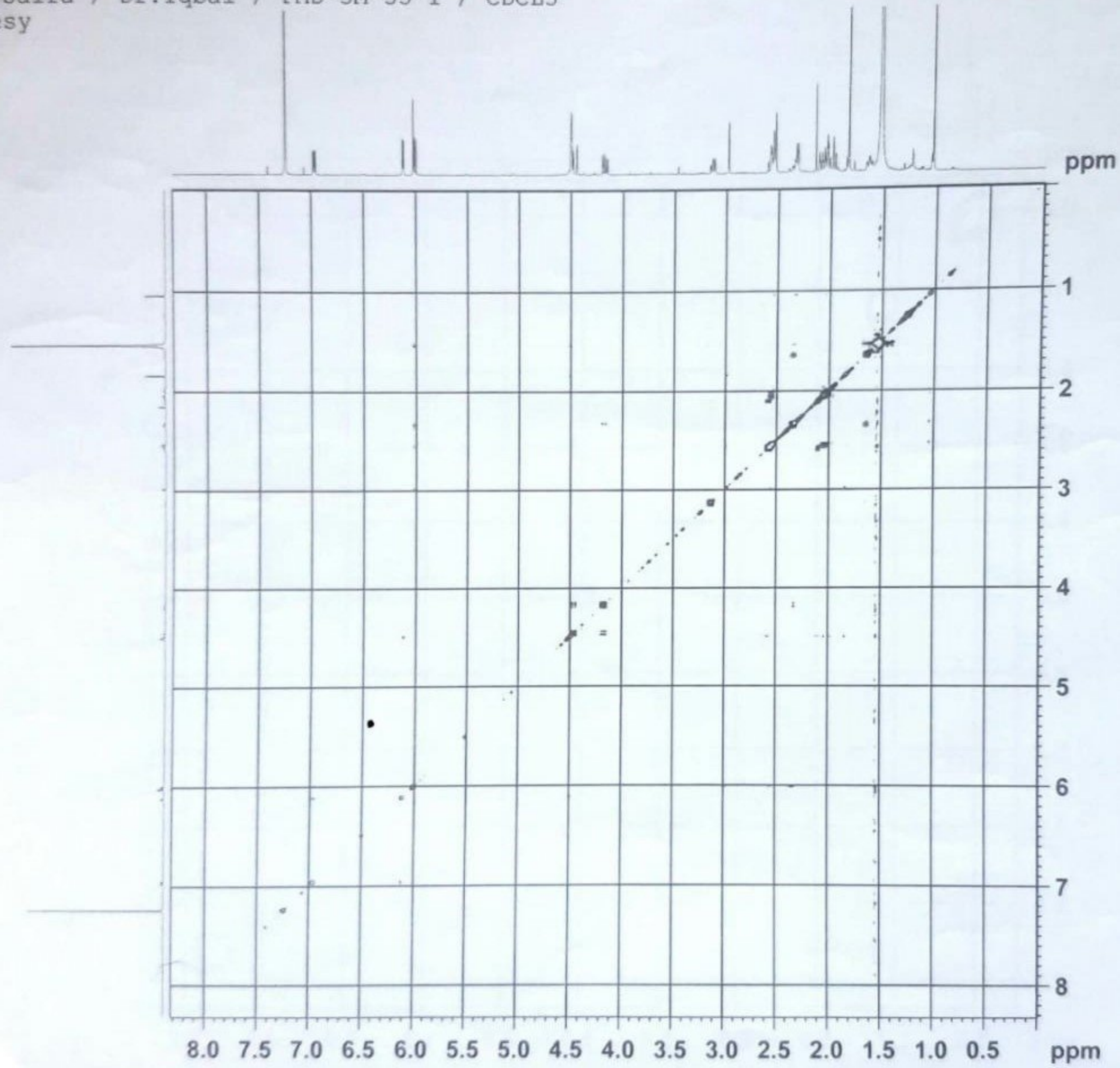

AVANCE NEO
600 MHz (LC)
Cryoprobe
Lab # 108

Current Data Parameters
NAME Sep23-20
EXPNO 5
PROCNO 1

F2 - Acquisition Parameters
Date_ 20200923
Time 20.15 h
INSTRUM Avance NEO 600MHz
PROBHD Z44896_0021 (C
PULPROG noesygpph
TD 2048
SOLVENT CDCl3
NS 32
DS 16
SWH 5000.000 Hz
FIDRES 4.882813 Hz
AQ 0.2048000 sec
RG 67.6187
DW 100.000 usec
DE 15.00 usec
TE 298.0 K
D0 0.00008727 sec
D1 1.50000000 sec
D8 0.80000001 sec
D16 0.00020000 sec
IN0 0.00020000 sec
TDav 1
SFO1 600.0325201 MHz
NUC1 1H
P1 10.00 usec
P2 20.00 usec
PLW1 4.25000000 W
GPNAM[1] SMSQ10.100
GPZ1 40.00 %
P16 1000.00 usec

F1 - Acquisition parameters
TD 256
SFO1 600.0325 MHz
FIDRES 39.062500 Hz
SW 8.333 ppm
FnMODE States-TPPI

F2 - Processing parameters
SI 1024
SF 600.0300266 MHz
WDW QSINE
SSB 2
LB 0 Hz
GB 0
PC 1.00

F1 - Processing parameters
SI 1024
MC2 States-TPPI
SF 600.0300266 MHz
WDW QSINE
SSB 2
LB 0 Hz
GB 0

CS CamScanner

Compound 1

Dr.Saira / Dr.Iqbal / PMD-3M-55-1 / CDCL3
HMBC

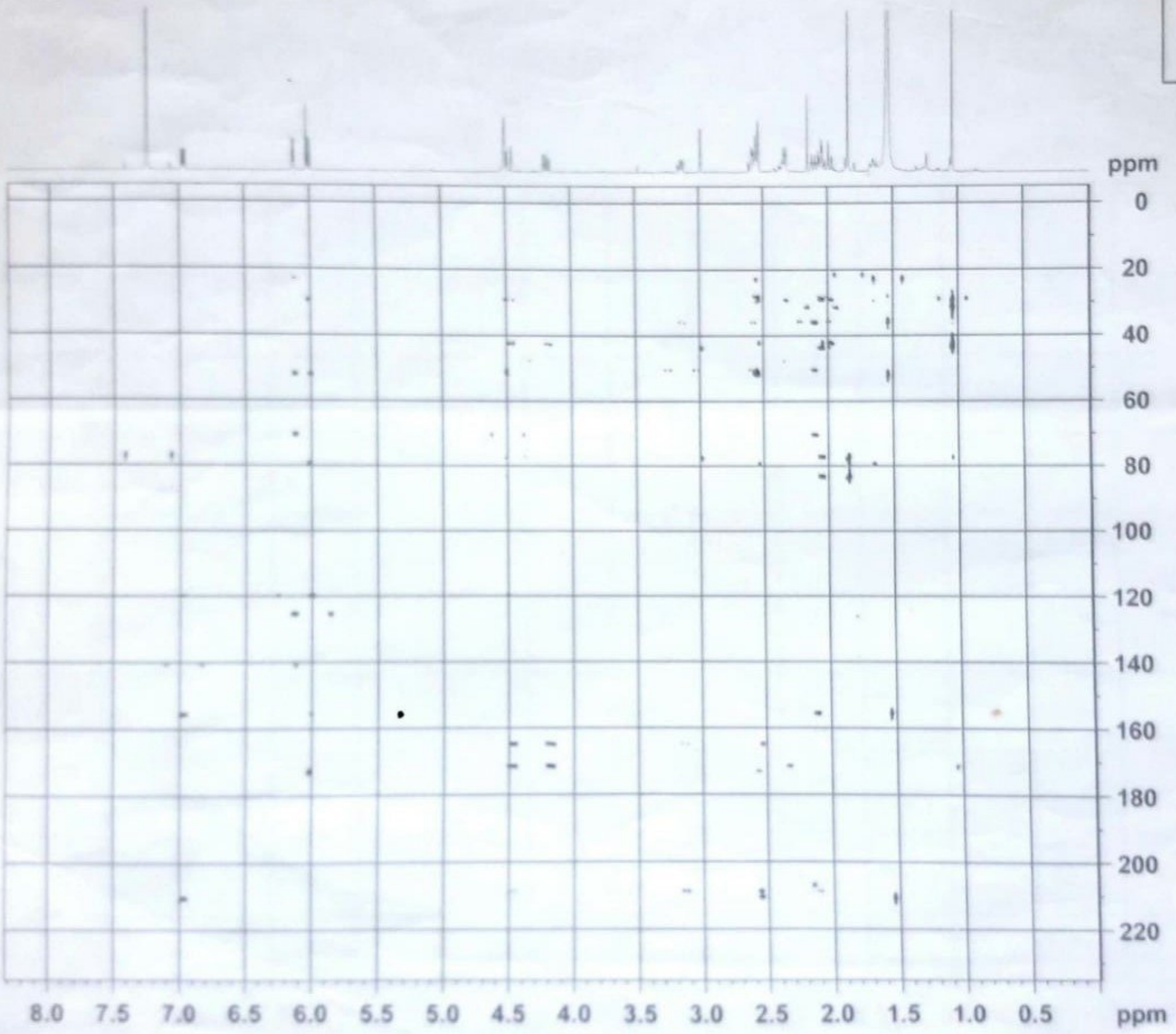

AVANCE NEO
600 MHz (LC)
Cryoprobe
Lab # 108

```
Current Data Parameters
NAME              Sep23-20
EXPNO                    7
PROCNO                   1

F2 - Acquisition Parameters
Date_             20200924
Time                8.58 h
INSTRUM   Avance NEO 600MHz
PROBHD    Z44896_0021 (C
PULPROG        hmbcgpndqf
TD                    4096
SOLVENT              CDCl3
NS                      64
DS                      16
SWH           5000.000 Hz
FIDRES        2.441406 Hz
AQ          0.4096000 sec
RG                     101
DW           100.000 usec
DE             15.00 usec
TE               298.0 K
CNST13          8.0000000
D0         0.00000300 sec
D1         1.50000000 sec
D6         0.06250000 sec
D16        0.00020000 sec
IN0        0.00001380 sec
TDav                     1
SFO1      600.0325201 MHz
NUC1                    1H
P1             10.00 usec
P2             20.00 usec
PLW1         4.25000000 W
SFO2      150.8950144 MHz
NUC2                   13C
P3             12.00 usec
PLW2        97.90100098 W
GPNAM[1]       SMSQ10.100
GPZ1               50.00 %
GPNAM[2]       SMSQ10.100
GPZ2               30.00 %
GPNAM[3]       SMSQ10.100
GPZ3               40.10 %
P16          1000.00 usec

F1 - Acquisition parameters
TD                     256
SFO1           150.895 MHz
FIDRES      283.061584 Hz
SW             240.113 ppm
FnMODE                  QF

F2 - Processing parameters
SI                    2048
SF        600.0300266 MHz
WDW                   SINE
SSB       0
LB        0 Hz
GB        0
PC                    1.00

F1 - Processing parameters
SI                    2048
MC2                     QF
SF        150.8776660 MHz
WDW                   SINE
SSB       0
LB        0 Hz
GB        0
```

Compound 6

Window Display Report

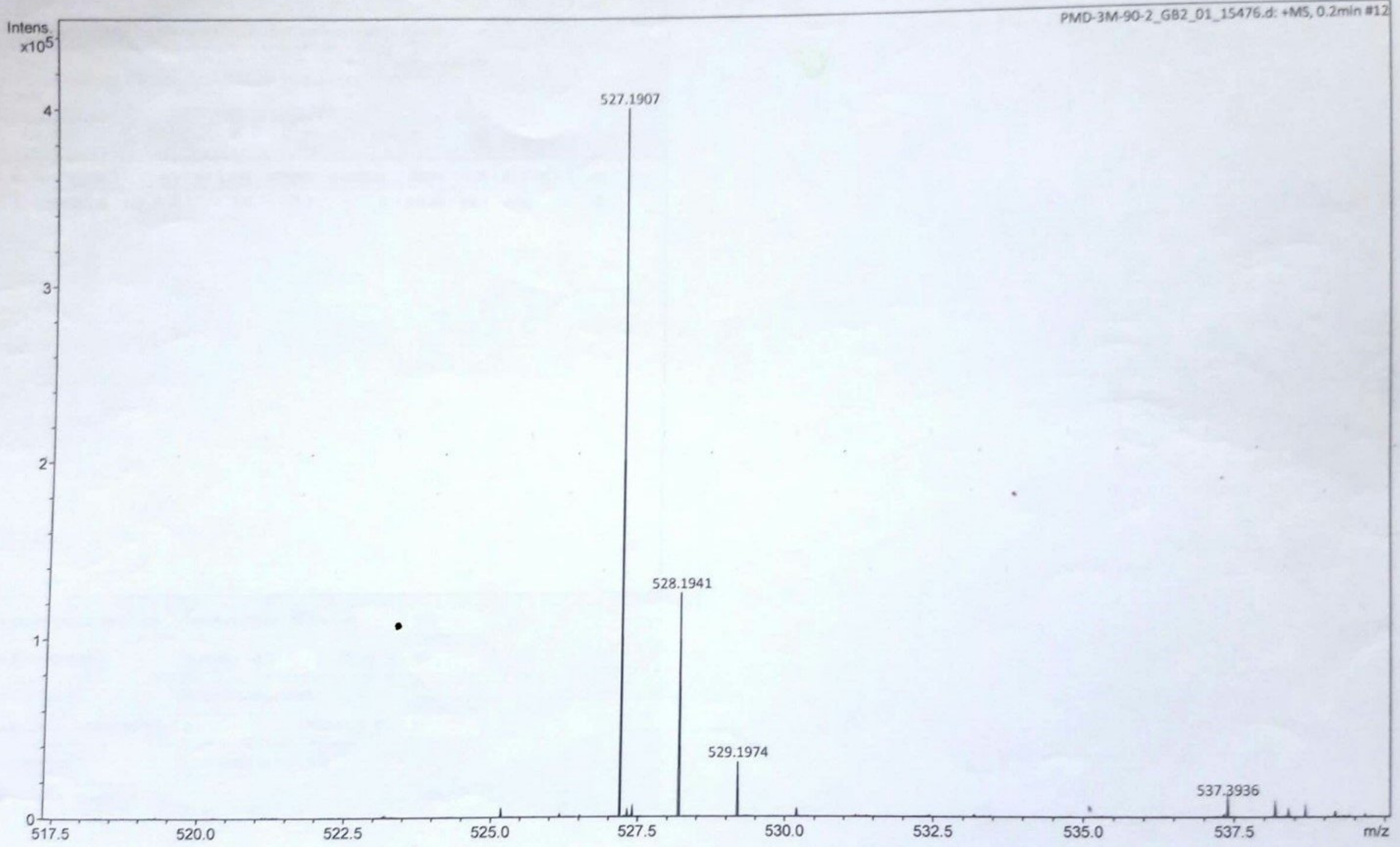

CamScanner

Compound 6

[illegible]wer formula:
Upper formula: N_0
N 0-0
Note: for m < 2000 the elements C, H, N, and O are considered implicitly.
Adducts, pos. M+H — Collect adducts
Adducts, neg. M-H
Measured m/z 527.1907 Tolerance: 10 ppm Charge: 1

Meas. m/z	#	Ion Formula	m/z	err [ppm]	mSigma	# mSigma	Score	rdb	e⁻ Conf	N-Rule
527.1907	1	C28H31O10	527.1912	1.0	3.7	1	100.00	13.5	even	ok

Automatically locate monoisotopic peak — Maximum number of formulae 500
Check rings plus double bonds — Minimum -0.5 Maximum 40
Electron configuration even
Filter H/C element ratio — Minimum H/C: 0 Maximum H/C: 3
Estimate carbon number — Generate immediately
Copy to SmartFormula Parameters — Show Pattern

Generate — Help

CS CamScanner

Compound 6

DR.SAIRA BANO/DR.IQBAL/PMD-3M-80-8/CDCL3
1H

AVANCE NEO
500MHz
LAB#118

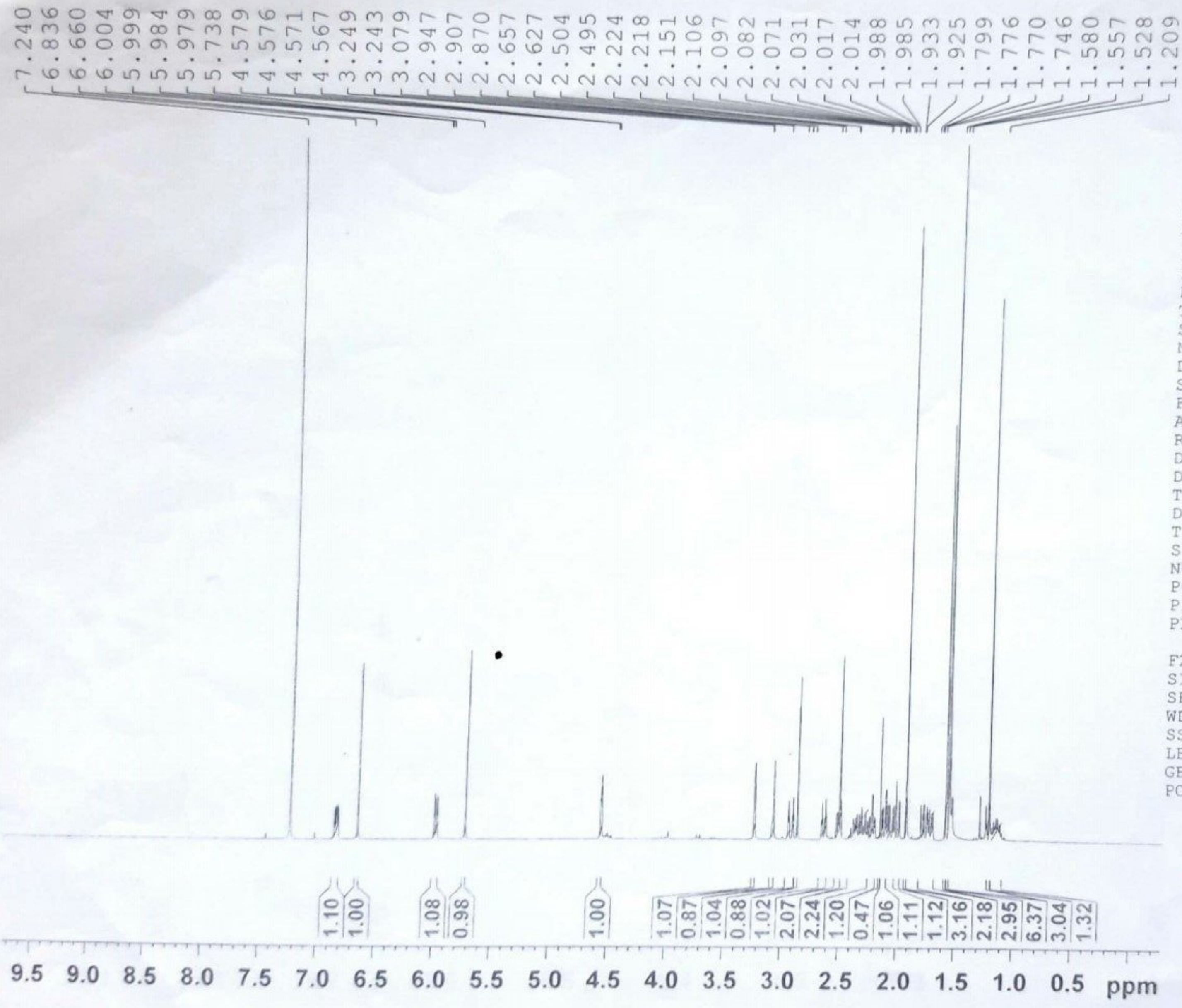

Current Data Parameters
NAME nov04-20
EXPNO 5
PROCNO 1

F2 - Acquisition Parameters
Date_ 20201104
Time 15.40 h
INSTRUM AVNeo_500
PROBHD Z8281_0183_(TX
PULPROG zg30
TD 65536
SOLVENT CDCl3
NS 128
DS 0
SWH 10000.000 Hz
FIDRES 0.305176 Hz
AQ 3.2767999 sec
RG 101
DW 50.000 usec
DE 11.14 usec
TE 300.0 K
D1 1.50000000 sec
TD0 1
SFO1 500.2340018 MHz
NUC1 1H
P0 2.67 usec
P1 8.00 usec
PLW1 18.45899963 W

F2 - Processing parameters
SI 32768
SF 500.2300220 MHz
WDW EM
SSB 0
LB 0.30 Hz
GB 0
PC 1.00

CamScanner

DR.SAIRA BANO/DR.IQBAL/PMD-3M-80-8/CDCL3
BB

Compound 6

AVANCE NEO
500MHz
LAB#118

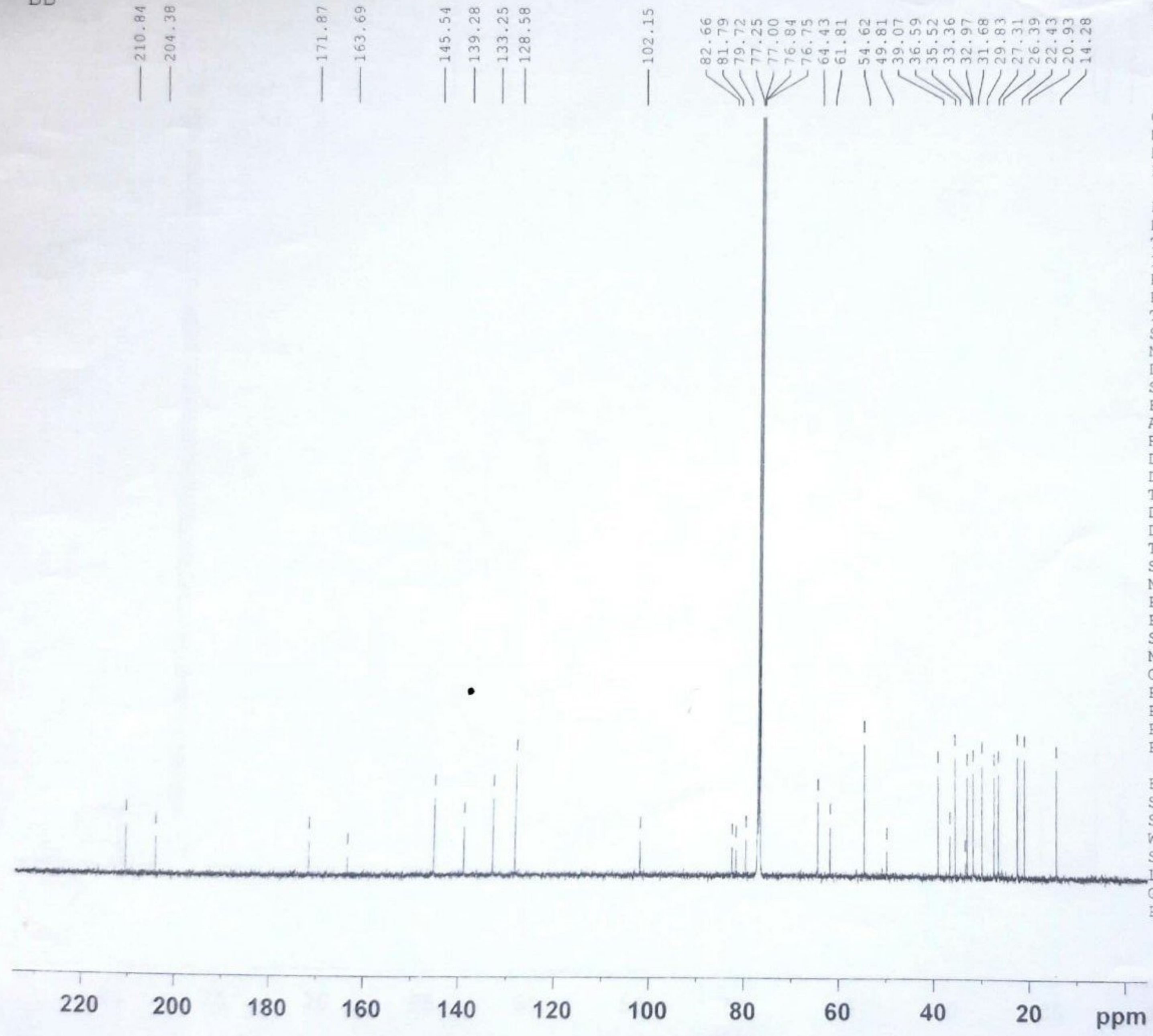

Current Data Parameters
NAME nov17-20
EXPNO 6
PROCNO 1

F2 - Acquisition Parameters
Date_ 20201120
Time 9.14 h
INSTRUM AVNeo_500
PROBHD Z8281_0183 (TX
PULPROG zgpg
TD 32768
SOLVENT CDCl3
NS 44217
DS 4
SWH 30120.482 Hz
FIDRES 1.838408 Hz
AQ 0.5439488 sec
RG 101
DW 16.600 usec
DE 12.00 usec
TE 300.0 K
D1 2.00000000 sec
D11 0.03000000 sec
TD0 25
SFO1 125.7973986 MHz
NUC1 13C
P1 12.00 usec
PLW1 229.03999329 W
SFO2 500.2320009 MHz
NUC2 1H
CPDPRG[2 waltz65
PCPD2 80.00 usec
PLW2 18.45899963 W
PLW12 0.18459000 W
PLW13 0.09284900 W

F2 - Processing parameters
SI 16384
SF 125.7829343 MHz
WDW EM
SSB 0
LB 1.00 Hz
GB 0
PC 1.40

CS CamScanner

Compound 6

DR.SAIRA BANO/DR.IQBAL/PMD-3M-80-8/CDCL3
Dept135

AVANCE NEO
500MHz
LAB#118

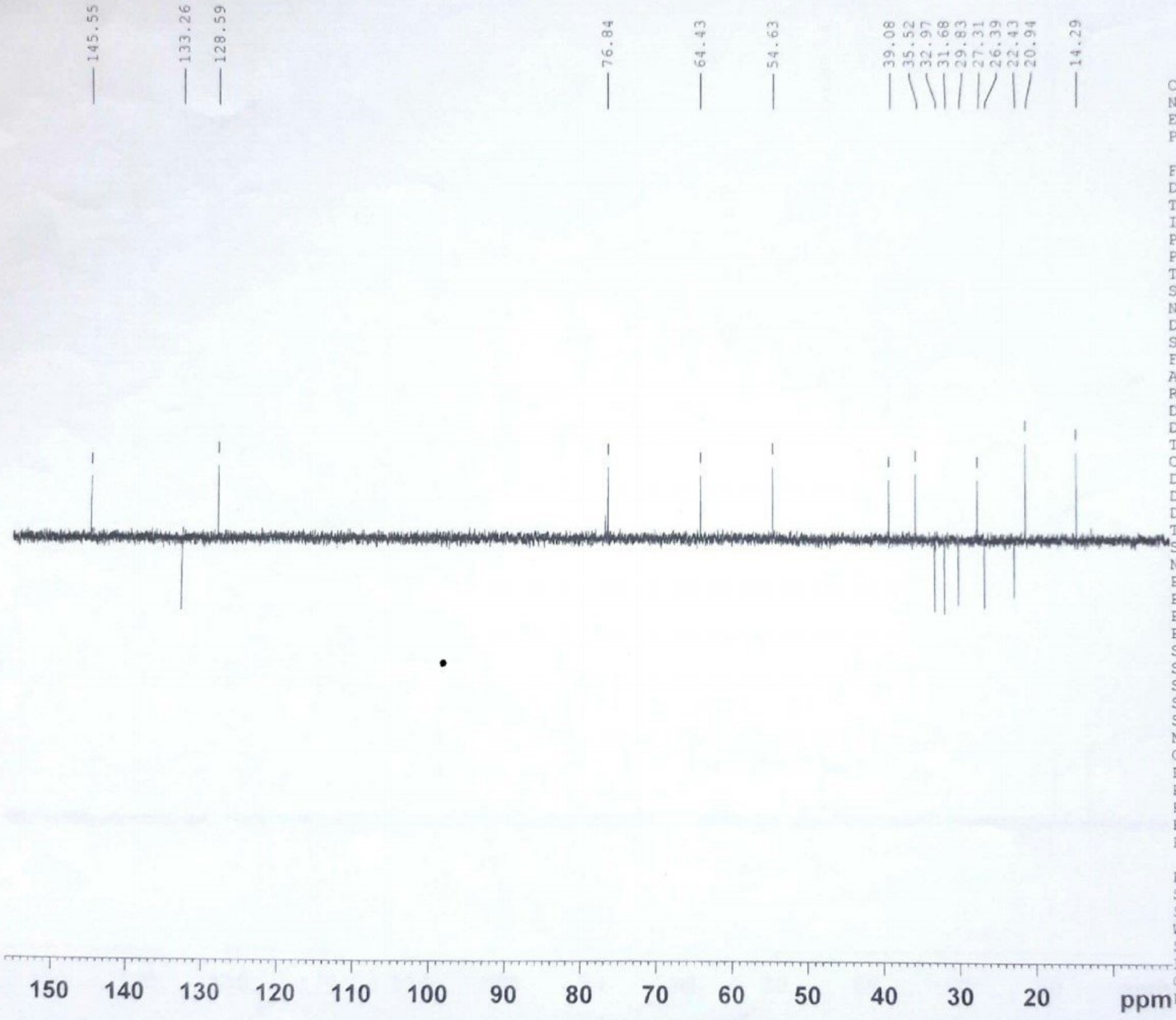

Current Data Parameters
NAME nov17-20
EXPNO 7
PROCNO 1

F2 - Acquisition Parameters
Date_ 20201119
Time 12.10 h
INSTRUM AVNeo_500
PROBHD Z8281_0183_(TX
PULPROG deptsp135
TD 32768
SOLVENT CDCl3
NS 7186
DS 4
SWH 25000.000 Hz
FIDRES 1.525879 Hz
AQ 0.6553600 sec
RG 101
DW 20.000 usec
DE 12.00 usec
TE 300.0 K
CNST2 145.0000000
D1 1.50000000 sec
D2 0.00344828 sec
D12 0.00002000 sec
TD0 10
SFO1 125.7948829 MHz
NUC1 13C
P1 12.00 usec
P13 2000.00 usec
PLW0 0 W
PLW1 229.03999329 W
SPNAM[5] Crp60comp.4
SPOAL5 0.500
SPOFFS5 0 Hz
SPW5 50.39199829 W
SFO2 500.2320009 MHz
NUC2 1H
CPDPRG[2 waltz65
P3 8.00 usec
P4 16.00 usec
PCPD2 80.00 usec
PLW2 18.45899963 W
PLW12 0.18459000 W

F2 - Processing parameters
SI 32768
SF 125.7829343 MHz
WDW EM
SSB 0
LB 1.00 Hz
GB 0
PC 1.40

CS CamScanner

Compound 6

DR.SAIRA BANO/DR.IQBAL/PMD-3M-80-8/CDCL3
Dept90

AVANCE NEO
500MHz
LAB#118

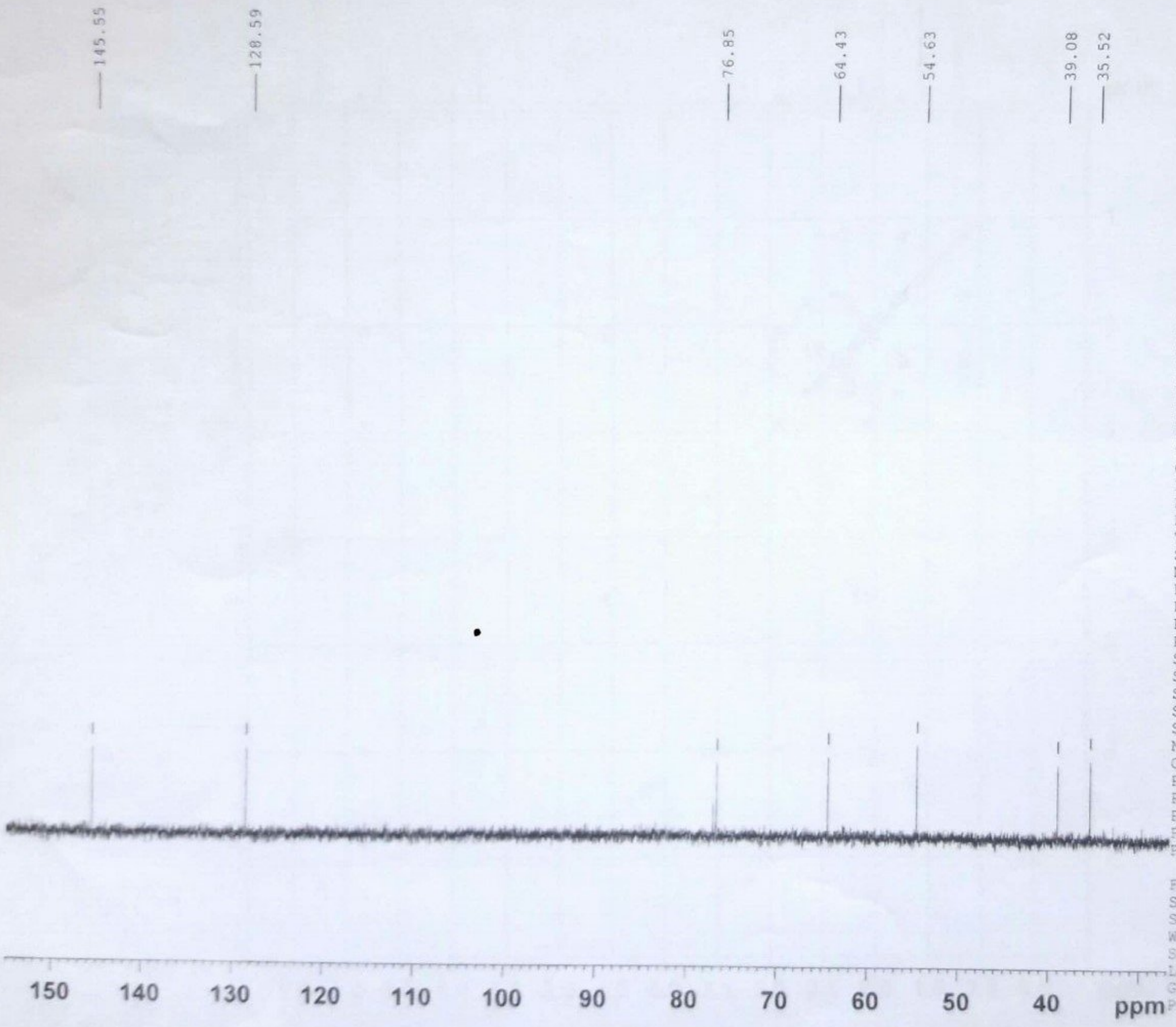

Current Data Parameters

NAME	nov17-20
EXPNO	8
PROCNO	1

F2 - Acquisition Parameters

Date_	20201119
Time	14.34 h
INSTRUM	AVNeo_500
PROBHD	Z8281_0183_(TX
PULPROG	deptsp90
TD	32768
SOLVENT	CDCl3
NS	3905
DS	8
SWH	25000.000 Hz
FIDRES	1.525879 Hz
AQ	0.6553600 sec
RG	101
DW	20.000 usec
DE	12.00 usec
TE	300.0 K
CNST2	145.0000000
D1	1.50000000 sec
D2	0.00344828 sec
D12	0.00002000 sec
TD0	4
SFO1	125.7948829 MHz
NUC1	13C
P1	12.00 usec
P13	2000.00 usec
PLW0	0 W
PLW1	229.03999329 W
SPNAM[5]	Crp60comp.4
SPOAL5	0.500
SPOFFS5	0 Hz
SPW5	50.39199829 W
SFO2	500.2320009 MHz
NUC2	1H
CPDPRG[2	waltz65
P3	8.00 usec
P4	16.00 usec
PCPD2	80.00 usec
PLW2	18.45899963 W
PLW12	0.18459000 W

F2 - Processing parameters

SI	32768
SF	125.7829343 MHz
WDW	EM
SSB	0
LB	1.00 Hz
GB	0
PC	1.40

Compound 6

DR.SAIRA BANO/DR.IQBAL/PMD-3M-80-8/CDCL3
HMBC

AVANCE NEO
500MHz
LAB#118

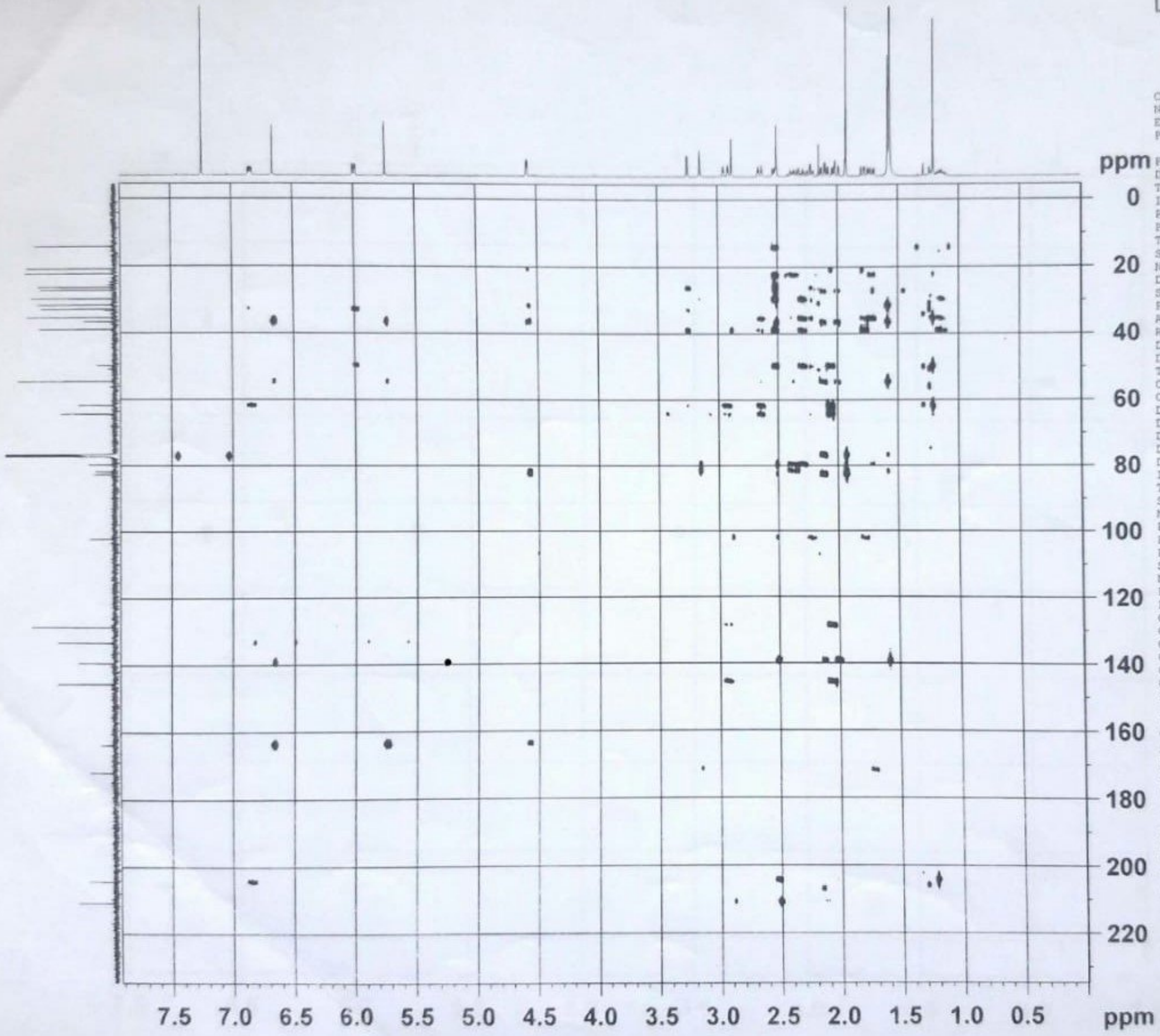


```
Current Data Parameters
NAME              nov17-20
EXPNO                    5
PROCNO                   1

F2 - Acquisition Parameters
Date_             20201118
Time                7.29 h
INSTRUM          AVNeo_500
PROBHD     Z8281_0183 (TX
PULPROG       hmbcgplpndqf
TD                    2048
SOLVENT              CDCl3
NS                      64
DS                      16
SWH             3968.254 Hz
FIDRES          3.875248 Hz
AQ             0.2580480 sec
RG                     101
DW               126.000 usec
DE                  6.50 usec
TE                 300.0 K
CNST2          145.0000000
CNST13          10.0000000
D0              0.00000300 sec
D1              2.00000000 sec
D2              0.00344828 sec
D6              0.05000000 sec
D16             0.00020000 sec
IN0             0.00001660 sec
TDav                     1
SFO1          500.2320009 MHz
NUC1                    1H
P1                    8.00 usec
P2                   16.00 usec
PLW1           18.45899963 W
SFO2          125.7973986 MHz
NUC2                   13C
P3                   12.00 usec
PLW2          229.03999329 W
GPNAM[1]         SMSQ10.100
GPZ1                 50.00 %
GPNAM[2]         SMSQ10.100
GPZ2                 30.00 %
GPNAM[3]         SMSQ10.100
GPZ3                 40.10 %
P16                1000.00 usec

F1 - Acquisition parameters
TD                     256
SFO1             125.7974 MHz
FIDRES          235.316269 Hz
SW                 239.436 ppm
FnMODE                  QF

F2 - Processing parameters
SI                    2048
SF             500.2300221 MHz
WDW                   SINE
SSB      0
LB       0 Hz
GB       0
PC                    1.40

F1 - Processing parameters
SI                    1024
MC2                     QF
SF             125.7829343 MHz
WDW                   SINE
SSB      0
LB       0 Hz
GB       0
```

Compound 6

DR.SAIRA BANO/DR.IQBAL/PMD-3M-80-8/CDCL3
NOESY

AVANCE NEO
500MHz
LAB#118

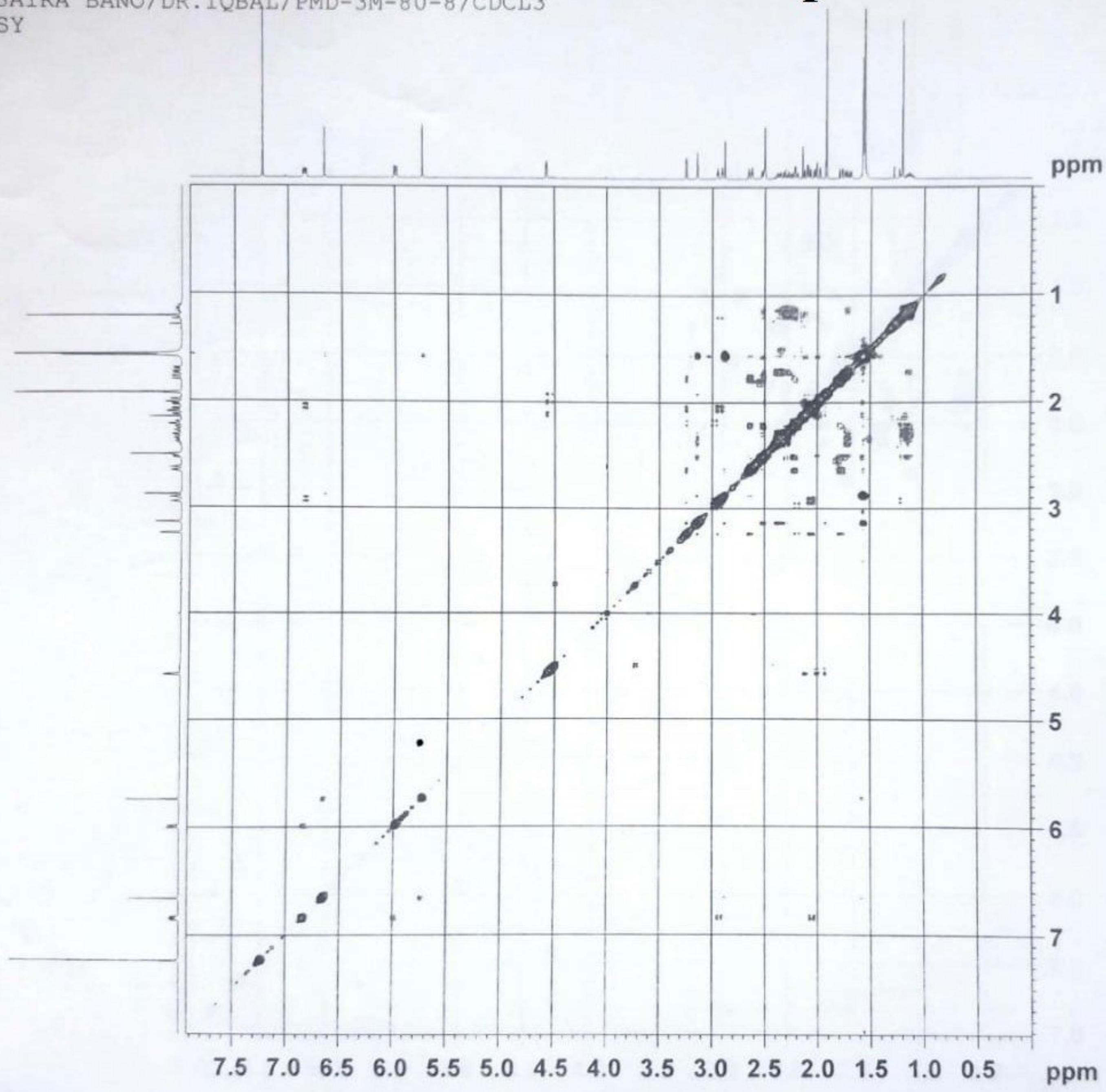


```
Current Data Parameters
NAME              nov17-20
EXPNO                    3
PROCNO                   1

F2 - Acquisition Parameters
Date_             20201117
Time                 14.47 h
INSTRUM          AVNeo_500
PROBHD     Z8281_0183 (TX
PULPROG         noesygpph
TD                    2048
SOLVENT              CDCl3
NS                      16
DS                       8
SWH               3968.254 Hz
FIDRES            3.875248 Hz
AQ               0.2580480 sec
RG                     101
DW                 126.000 usec
DE                    6.50 usec
TE                   300.0 K
D0              0.00011581 sec
D1              2.00000000 sec
D8              0.80000001 sec
D16             0.00020000 sec
IN0             0.00025200 sec
TDav                     1
SFO1           500.2320009 MHz
NUC1                    1H
P1                    8.00 usec
P2                   16.00 usec
PLW1           18.45899963 W
GPNAM[1]        SMSQ10.100
GPZ1                 40.00 %
P16                1000.00 usec

F1 - Acquisition parameters
TD                     256
SFO1               500.232 MHz
FIDRES           31.001984 Hz
SW                   7.933 ppm
FnMODE                TPPI

F2 - Processing parameters
SI                    1024
SF             500.2300221 MHz
WDW                  QSINE
SSB                      2
LB         0 Hz
GB         0
PC                    1.00

F1 - Processing parameters
SI                    1024
MC2                   TPPI
SF             500.2300221 MHz
WDW                  QSINE
SSB                      2
LB         0 Hz
GB         0
```

CS CamScanner

DR.SAIRA BANO/DR.IQBAL/PMD-3M-80-8/CDCL3
HSQC

Compound 6

AVANCE NEO
500MHz
LAB#118

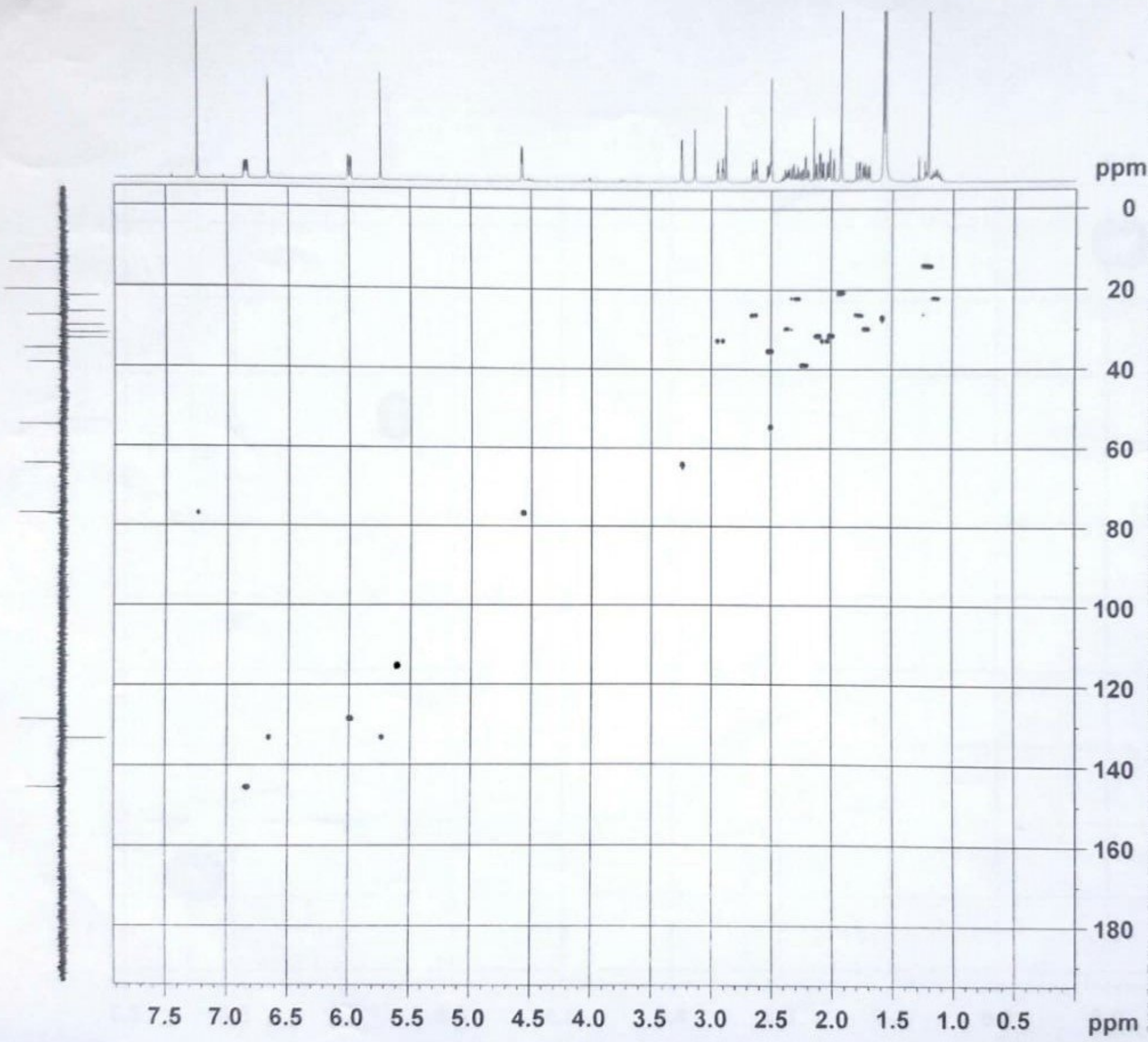

Current Data Parameters

NAME	nov17-20
EXPNO	4
PROCNO	1

F2 - Acquisition Parameters

Date_	20201117
Time	20.55 h
INSTRUM	AVNeo_500
PROBHD	Z8281_0183_(TX
PULPROG	hsqcedetgp
TD	1024
SOLVENT	CDCl3
NS	32
DS	8
SWH	3968.254 Hz
FIDRES	7.750496 Hz
AQ	0.1290240 sec
RG	101
DW	126.000 usec
DE	6.50 usec
TE	300.0 K
CNST2	145.0000000
D0	0.00000300 sec
D1	1.50000000 sec
D4	0.00172414 sec
D11	0.03000000 sec
D13	0.00000400 sec
D16	0.00020000 sec
D21	0.00345000 sec
IN0	0.00001990 sec
TDav	1
ZGOPTNS	
SFO1	500.2320009 MHz
NUC1	1H
P1	8.00 usec
P2	16.00 usec
P28	1000.00 usec
PLW1	18.45899963 W
SFO2	125.7948829 MHz
NUC2	13C
CPDPRG[2	garp
P3	12.00 usec
P4	24.00 usec
PCPD2	70.00 usec
PLW2	229.03999329 W
PLW12	6.73089981 W
GPNAM[1]	SMSQ10.100
GPZ1	80.00 %
GPNAM[2]	SMSQ10.100
GPZ2	20.10 %
P16	1000.00 usec

F1 - Acquisition parameters

TD	256
SFO1	125.7949 MHz
FIDRES	196.293976 Hz
SW	199.735 ppm
FnMODE	Echo-Antiecho

F2 - Processing parameters

SI	1024
SF	500.2300221 MHz
WDW	QSINE
SSB	2
LB	0 Hz
GB	0
PC	1.40

F1 - Processing parameters

SI	1024
MC2	echo-antiecho
SF	125.7829343 MHz
WDW	QSINE
SSB	2
LB	0 Hz
GB	0

CS CamScanner

Compound 6

DR.SAIRA BANO/DR.IQBAL/PMD-3M-80-8/CDCL3
COSY

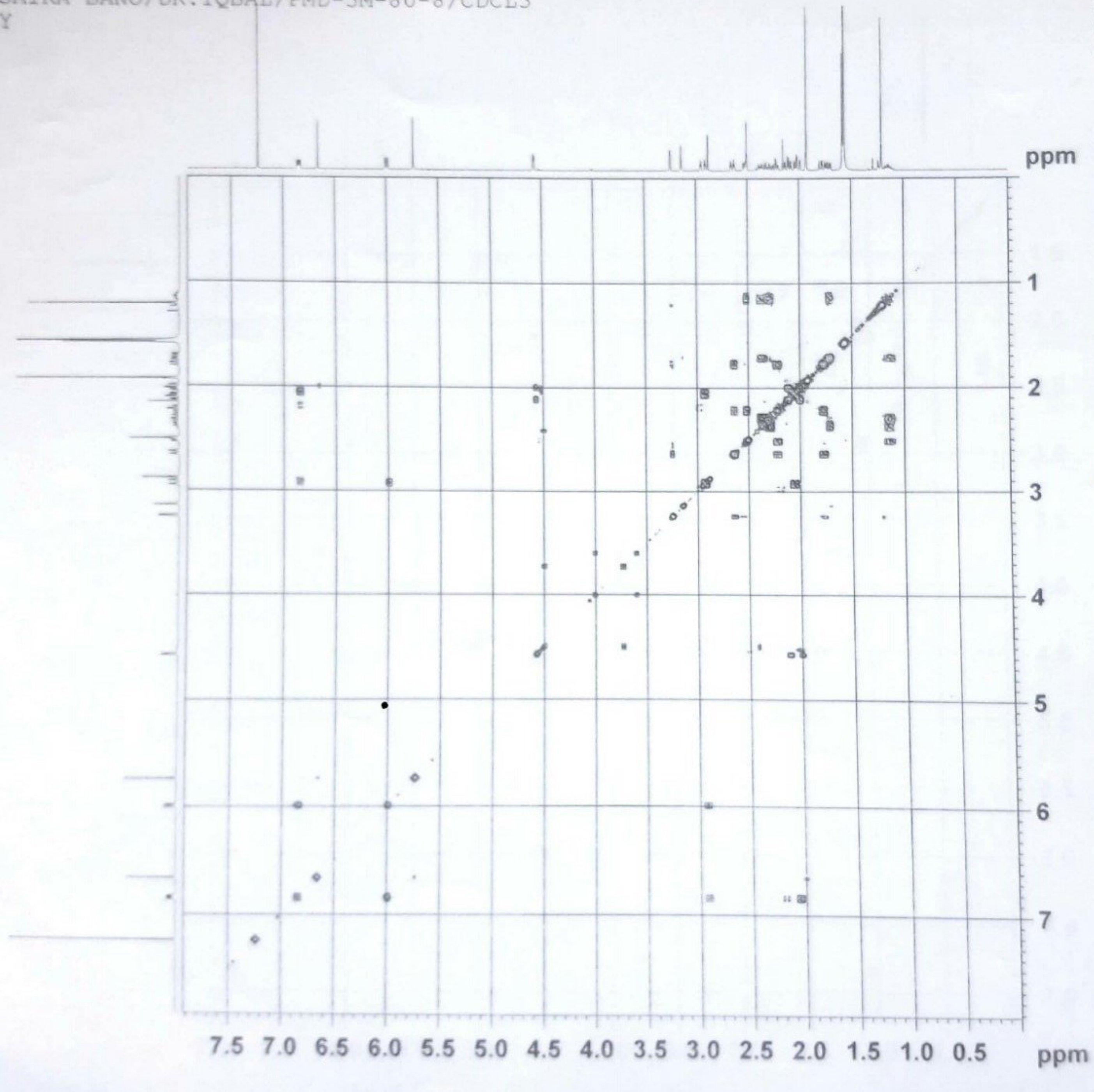

AVANCE NEO
500MHz
LAB#118

Current Data Parameters

NAME	nov17-20
EXPNO	2
PROCNO	1

F2 - Acquisition Parameters

Date_	20201117
Time	11.17 h
INSTRUM	AVNeo_500
PROBHD	Z8281_0183_(TX
PULPROG	cosygpqf
TD	2048
SOLVENT	CDCl3
NS	8
DS	8
SWH	3968.254 Hz
FIDRES	3.875248 Hz
AQ	0.2580480 sec
RG	101
DW	126.000 usec
DE	6.50 usec
TE	300.0 K
D0	0.00000300 sec
D1	1.50000000 sec
D13	0.00000400 sec
D16	0.00020000 sec
IN0	0.00025200 sec
TDav	1
SFO1	500.2320009 MHz
NUC1	1H
P0	8.00 usec
P1	8.00 usec
PLW1	18.45899963 W
GPNAM[1]	SMSQ10.100
GPZ1	10.00 %
P16	1000.00 usec

F1 - Acquisition parameters

TD	256
SFO1	500.232 MHz
FIDRES	31.001984 Hz
SW	7.933 ppm
FnMODE	QF

F2 - Processing parameters

SI	1024
SF	500.2300221 MHz
WDW	QSINE
SSB	0
LB	0 Hz
GB	0
PC	1.40

F1 - Processing parameters

SI	1024
MC2	QF
SF	500.2300221 MHz
WDW	QSINE
SSB	0
LB	0 Hz
GB	0

CS CamScanner

Compound 8

Window Display Report

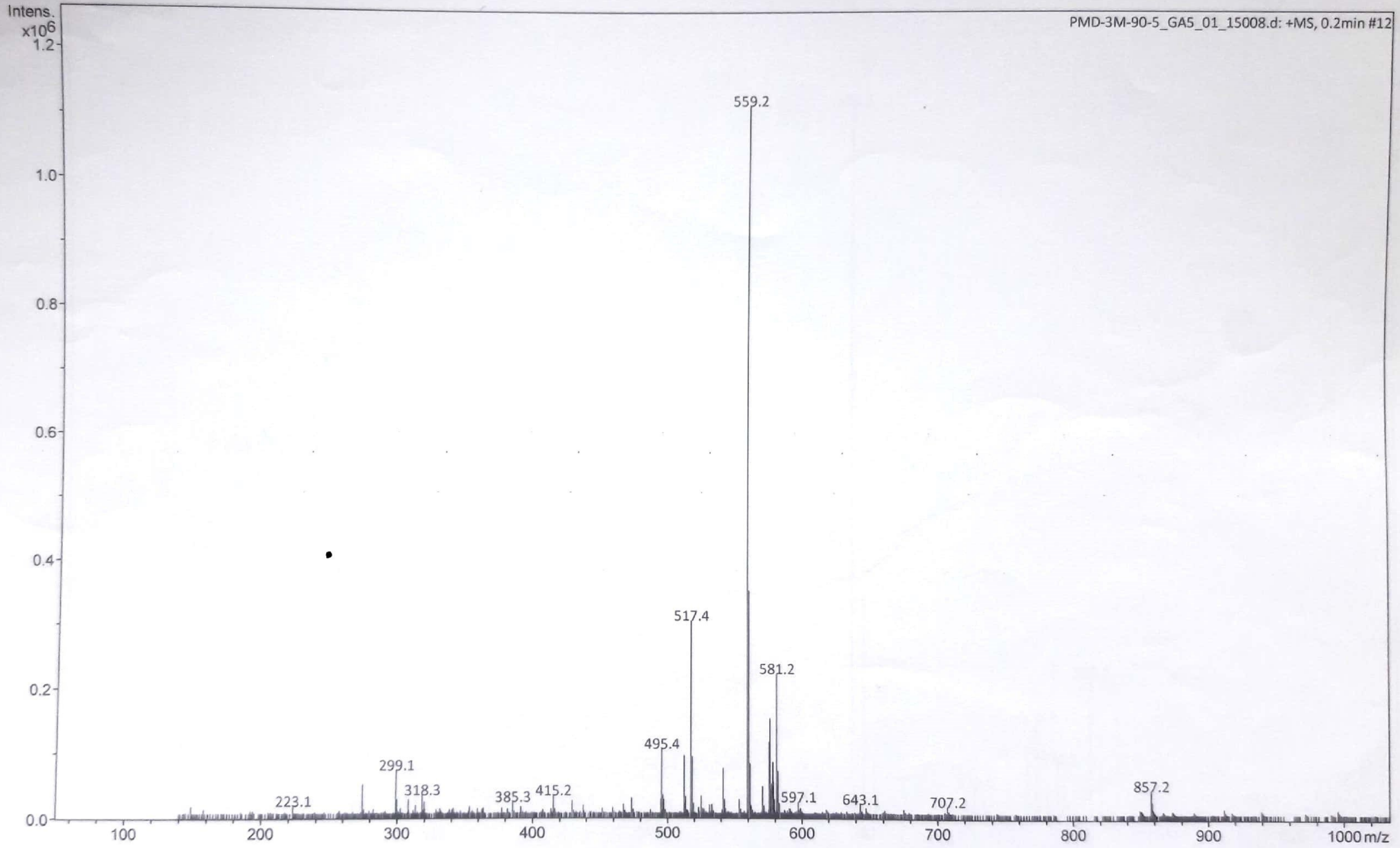

Compound 8

ower formula: C_{21}

Upper formula: C_{53}

C 21-53

Generate

Help

Note: for m < 2000 the elements C, H, N, and O are considered implicitly.

Adducts, pos. M+H

Collect adducts

Adducts, neg. M-H

Measured m/z 559.1811 Tolerance: 2 ppm Charge: 1

Meas. m/z	#	Ion Formula	m/z	err [ppm]	mSigma	# mSigma	Score	rdb	e⁻ Conf	N-Rule
559.1811	2	C26H19N14O2	559.1810	-0.1	13.7	2	79.21	24.5	even	ok
	1	C28H31O12	559.1810	-0.1	0.9	1	100.00	13.5	even	ok
	3	C41H23N2O	559.1805	-1.0	73.4	3	11.41	31.5	even	ok

☐ Automatically locate monoisotopic peak — Maximum number of formulae: 500

☑ Check rings plus double bonds — Minimum: -0.5 — Maximum: 40

Electron configuration: even

☑ Filter H/C element ratio — Minimum H/C: 0 — Maximum H/C: 3

☑ Estimate carbon number — ☑ Generate immediately

Copy to SmartFormula Parameters

Show Pattern

Compound 8

DR.SAIRA BANO/DR.IQBAL/PMD-3M-90-5/CD3OD
1H

AVANCE NEO
500MHz
LAB#118

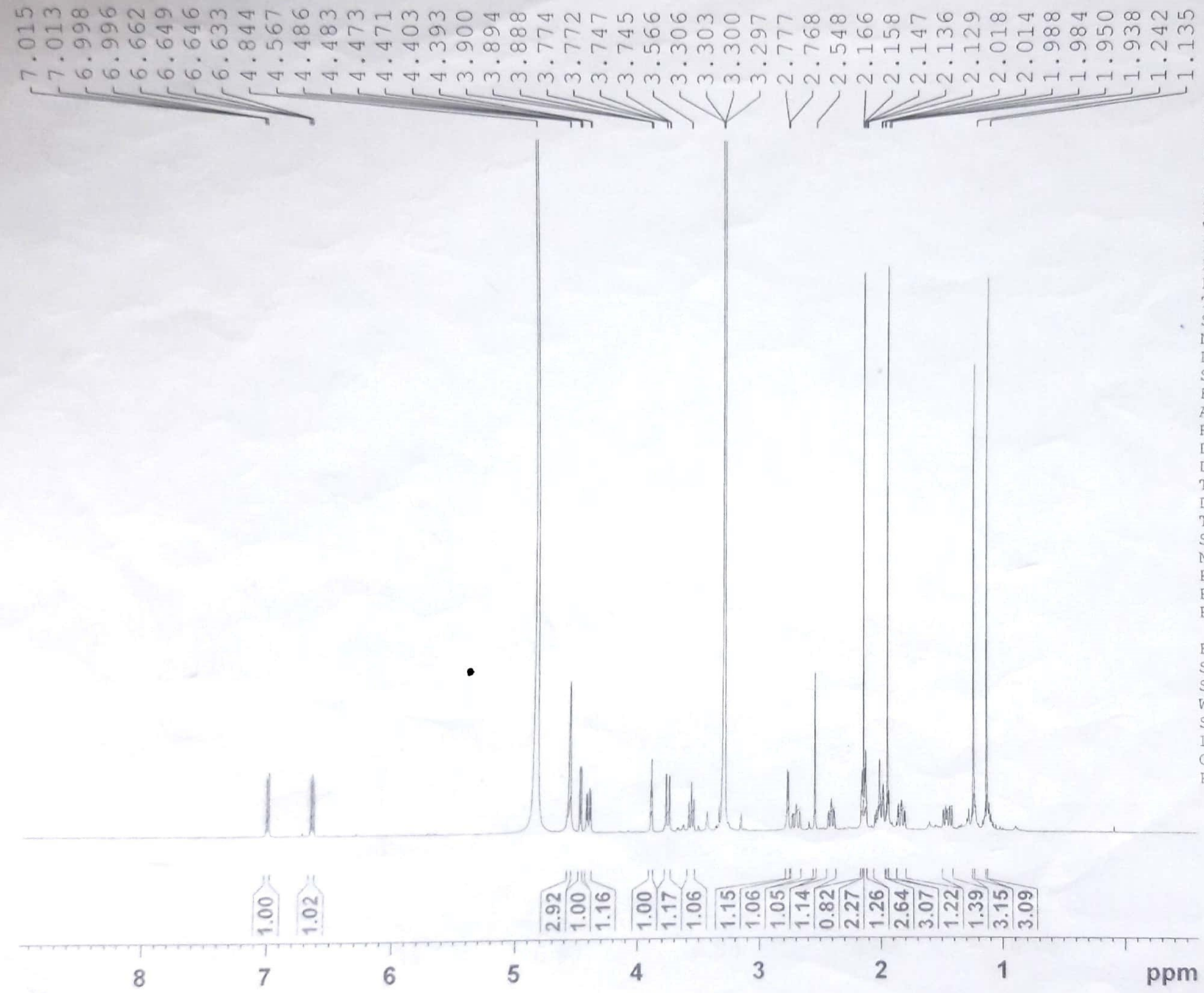

Current Data Parameters
NAME sep10-20
EXPNO 1
PROCNO 1

F2 - Acquisition Parameters
Date_ 20200910
Time 10.21 h
INSTRUM AVNeo_500
PROBHD Z8281_0183_(TX
PULPROG zg30
TD 65536
SOLVENT MeOD
NS 128
DS 0
SWH 10000.000 Hz
FIDRES 0.305176 Hz
AQ 3.2767999 sec
RG 101
DW 50.000 usec
DE 11.14 usec
TE 300.0 K
D1 1.50000000 sec
TD0 1
SFO1 500.2340018 MHz
NUC1 1H
P0 2.67 usec
P1 8.00 usec
PLW1 18.45899963 W

F2 - Processing parameters
SI 32768
SF 500.2300145 MHz
WDW EM
SSB 0
LB 0.30 Hz
GB 0
PC 1.00
