# Supplementary material for: Anti-leishmanial physalins—Phytochemical investigation, in vitro evaluation against clinical and MIL-resistant L. tropica strains and in silico studies
Source: PLoS One. 2022 Nov 28;17(11):e0274543. doi: 10.1371/journal.pone.0274543 (PMC9704608; doi:10.1371/journal.pone.0274543)

checkCIF/PLATON report

Structure factors have been supplied for datablock(s) shelx

THIS REPORT IS FOR GUIDANCE ONLY. IF USED AS PART OF A REVIEW PROCEDURE FOR PUBLICATION, IT SHOULD NOT REPLACE THE EXPERTISE OF AN EXPERIENCED CRYSTALLOGRAPHIC REFEREE.

No syntax errors found. CIF dictionary Interpreting this report

Datablock: shelx

```
Bond precision:  C-C = 0.0071 A          Wavelength=1.54178

Cell:              a=9.7870(4)     b=15.0700(7)     c=16.3581(7)
                   alpha=90        beta=90          gamma=90
Temperature:       100 K

                 Calculated                Reported
Volume           2412.66(18)               2412.66(18)
Space group      P 21 21 21                P 21 21 21
Hall group       P 2ac 2ab                 P 2ac 2ab
Moiety formula   C28 H31 N O9, H2 O        ?
Sum formula      C28 H33 N O10             C28 H33 N O10
Mr               543.55                    543.55
Dx,g cm-3        1.496                     1.496
Z                4                         4
Mu (mm-1)        0.954                     0.954
F000             1152.0                    1152.0
F000'            1155.98
h,k,lmax         11,18,19                  11,18,19
Nref             4412[ 2508]               4364
Tmin,Tmax        0.934,0.972
Tmin'            0.424

Correction method= Not given

Data completeness= 1.74/0.99      Theta(max)= 68.135

R(reflections)= 0.0551( 3700)     wR2(reflections)= 0.1573( 4364)

S = 1.017                    Npar= 375
```

The following ALERTS were generated. Each ALERT has the format
test-name_ALERT_alert-type_alert-level.
Click on the hyperlinks for more details of the test.

Alert level C

```
PLAT089_ALERT_3_C Poor Data / Parameter Ratio (Zmax < 18) ........       6.64 Note
PLAT340_ALERT_3_C Low Bond Precision on  C-C Bonds ...............    0.00713 Ang.
PLAT354_ALERT_3_C Short  O-H (X0.82,N0.98A)  O5       - Ho4      .       0.71 Ang.
PLAT911_ALERT_3_C Missing FCF Refl Between Thmin & STh/L=    0.600         17 Report
PLAT975_ALERT_2_C Check Calcd Resid. Dens.  0.92A   From O1              0.45 eA-3
```

Alert level G

```
PLAT002_ALERT_2_G Number of Distance or Angle Restraints on AtSite          3 Note
PLAT063_ALERT_4_G Crystal Size Possibly too Large for Beam Size ..       0.90 mm
PLAT172_ALERT_4_G The CIF-Embedded .res File Contains DFIX Records          2 Report
PLAT720_ALERT_4_G Number of Unusual/Non-Standard Labels ..........          5 Note
PLAT791_ALERT_4_G Model has Chirality at C6          (Sohnke SpGr)          R Verify
PLAT791_ALERT_4_G Model has Chirality at C8          (Sohnke SpGr)          R Verify
PLAT791_ALERT_4_G Model has Chirality at C9          (Sohnke SpGr)          S Verify
PLAT791_ALERT_4_G Model has Chirality at C10         (Sohnke SpGr)          R Verify
PLAT791_ALERT_4_G Model has Chirality at C13         (Sohnke SpGr)          S Verify
PLAT791_ALERT_4_G Model has Chirality at C16         (Sohnke SpGr)          R Verify
PLAT791_ALERT_4_G Model has Chirality at C17         (Sohnke SpGr)          R Verify
PLAT791_ALERT_4_G Model has Chirality at C20         (Sohnke SpGr)          S Verify
PLAT791_ALERT_4_G Model has Chirality at C22         (Sohnke SpGr)          R Verify
PLAT791_ALERT_4_G Model has Chirality at C24         (Sohnke SpGr)          S Verify
PLAT791_ALERT_4_G Model has Chirality at C25         (Sohnke SpGr)          S Verify
PLAT860_ALERT_3_G Number of Least-Squares Restraints .............          3 Note
PLAT883_ALERT_1_G No Info/Value for _atom_sites_solution_primary .     Please Do !
PLAT909_ALERT_3_G Percentage of I>2sig(I) Data at Theta(Max) Still        70% Note
PLAT910_ALERT_3_G Missing # of FCF Reflection(s) Below Theta(Min).          1 Note
PLAT978_ALERT_2_G Number C-C Bonds with Positive Residual Density.          2 Info
```

```
   0 ALERT level A = Most likely a serious problem - resolve or explain
   0 ALERT level B = A potentially serious problem, consider carefully
   5 ALERT level C = Check. Ensure it is not caused by an omission or oversight
  20 ALERT level G = General information/check it is not something unexpected

   1 ALERT type 1 CIF construction/syntax error, inconsistent or missing data
   3 ALERT type 2 Indicator that the structure model may be wrong or deficient
   7 ALERT type 3 Indicator that the structure quality may be low
  14 ALERT type 4 Improvement, methodology, query or suggestion
   0 ALERT type 5 Informative message, check
```

Validation response form

Please find below a validation response form (VRF) that can be filled in and pasted into your CIF.

```
# start Validation Reply Form
_vrf_PLAT089_shelx
;
PROBLEM: Poor Data / Parameter Ratio (Zmax < 18) ........       6.64 Note
RESPONSE: ...
;
_vrf_PLAT340_shelx
;
PROBLEM: Low Bond Precision on  C-C Bonds ...............    0.00713 Ang.
RESPONSE: ...
;
_vrf_PLAT354_shelx
;
PROBLEM: Short  O-H (X0.82,N0.98A)  O5       - Ho4      .       0.71 Ang.
```

```
RESPONSE: ...
;
_vrf_PLAT911_shelx
;
PROBLEM: Missing FCF Refl Between Thmin & STh/L=    0.600         17 Report
RESPONSE: ...
;
_vrf_PLAT975_shelx
;
PROBLEM: Check Calcd Resid. Dens.  0.92A   From O1              0.45 eA-3
RESPONSE: ...
;
# end Validation Reply Form
```

It is advisable to attempt to resolve as many as possible of the alerts in all categories. Often the minor alerts point to easily fixed oversights, errors and omissions in your CIF or refinement strategy, so attention to these fine details can be worthwhile. In order to resolve some of the more serious problems it may be necessary to carry out additional measurements or structure refinements. However, the purpose of your study may justify the reported deviations and the more serious of these should normally be commented upon in the discussion or experimental section of a paper or in the "special_details" fields of the CIF. checkCIF was carefully designed to identify outliers and unusual parameters, but every test has its limitations and alerts that are not important in a particular case may appear. Conversely, the absence of alerts does not guarantee there are no aspects of the results needing attention. It is up to the individual to critically assess their own results and, if necessary, seek expert advice.

Publication of your CIF in IUCr journals

A basic structural check has been run on your CIF. These basic checks will be run on all CIFs submitted for publication in IUCr journals (*Acta Crystallographica*, *Journal of Applied Crystallography*, *Journal of Synchrotron Radiation*); however, if you intend to submit to *Acta Crystallographica Section C* or *E* or *IUCrData*, you should make sure that full publication checks are run on the final version of your CIF prior to submission.

Publication of your CIF in other journals

Please refer to the *Notes for Authors* of the relevant journal for any special instructions relating to CIF submission.

PLATON version of 18/09/2020; check.def file version of 20/08/2020

Datablock shelx - ellipsoid plot

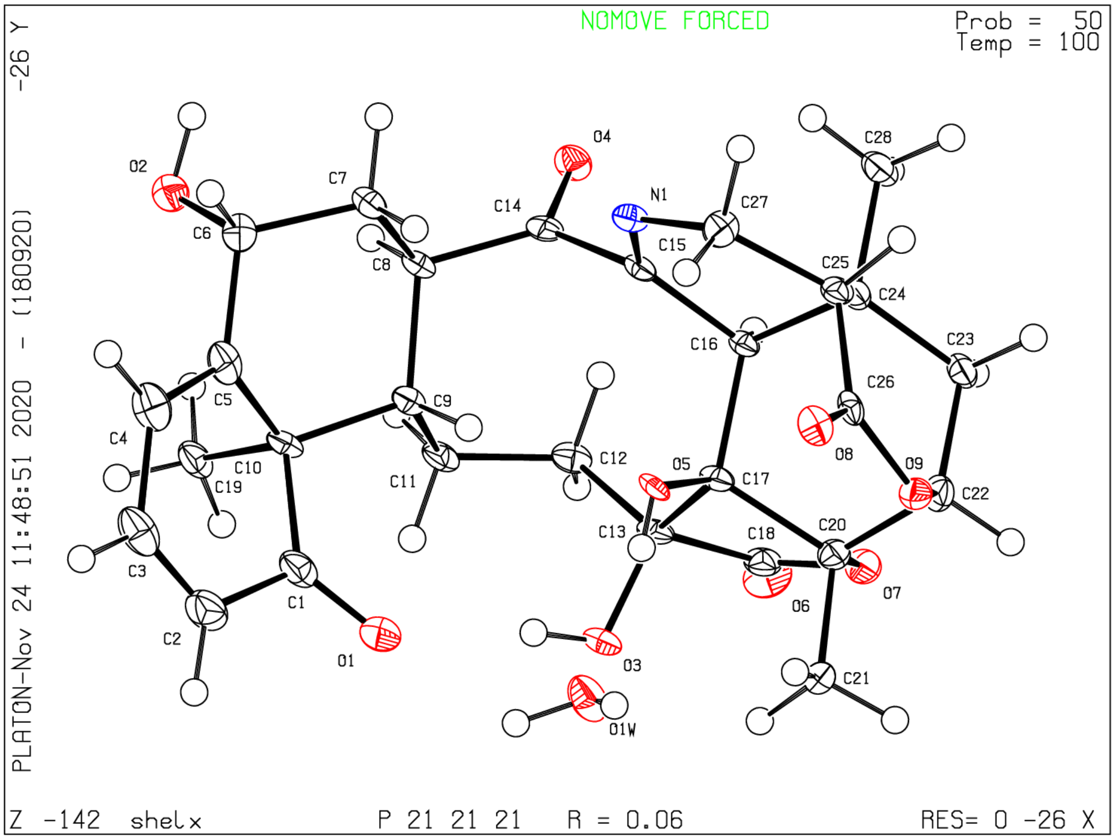

Supplement: S2 File — (PDF) [file pone.0274543.s027.pdf]
