# Supplementary material for: Anti-leishmanial physalins—Phytochemical investigation, in vitro evaluation against clinical and MIL-resistant L. tropica strains and in silico studies
Source: PLoS One. 2022 Nov 28;17(11):e0274543. doi: 10.1371/journal.pone.0274543 (PMC9704608; doi:10.1371/journal.pone.0274543)

checkCIF/PLATON report

Structure factors have been supplied for datablock(s) shelx

THIS REPORT IS FOR GUIDANCE ONLY. IF USED AS PART OF A REVIEW PROCEDURE FOR PUBLICATION, IT SHOULD NOT REPLACE THE EXPERTISE OF AN EXPERIENCED CRYSTALLOGRAPHIC REFEREE.

No syntax errors found. CIF dictionary Interpreting this report

Datablock: shelx

```
Bond precision:  C-C = 0.0078 A          Wavelength=1.54178

Cell:            a=7.4821(5)    b=11.7099(7)       c=15.2299(11)
                 alpha=90       beta=95.803(5)     gamma=90
Temperature:     100 K

                Calculated                 Reported
Volume          1327.52(15)                1327.52(15)
Space group     P 21                       P 21
Hall group      P 2yb                      P 2yb
Moiety formula  C28 H30 O12, C H4 O, H2 O  ?
Sum formula     C29 H36 O14                C29 H36 O14
Mr              608.58                     608.58
Dx,g cm-3       1.523                      1.522
Z               2                          2
Mu (mm-1)       1.038                      1.038
F000            644.0                      644.0
F000'           646.36
h,k,lmax        9,14,18                    9,14,18
Nref            4849[ 2553]                4824
Tmin,Tmax       0.851,0.990
Tmin'           0.756

Correction method= Not given

Data completeness= 1.89/0.99      Theta(max)= 68.237

R(reflections)= 0.0521( 3703)     wR2(reflections)= 0.1300( 4824)

S = 1.020                  Npar= 406
```

The following ALERTS were generated. Each ALERT has the format
test-name_ALERT_alert-type_alert-level.
Click on the hyperlinks for more details of the test.

Alert level C

```
RINTA01_ALERT_3_C  The value of Rint is greater than 0.12
            Rint given   0.134
PLAT057_ALERT_3_C Correction for Absorption Required   RT(exp) ...       1.16 Do !
PLAT089_ALERT_3_C Poor Data / Parameter Ratio (Zmax < 18) ........       6.28 Note
PLAT223_ALERT_4_C Solv./Anion  Resd 3  H   Ueq(max)/Ueq(min) Range        5.5 Ratio
PLAT340_ALERT_3_C Low Bond Precision on  C-C Bonds ...............    0.00781 Ang.
PLAT355_ALERT_3_C Long   O-H (X0.82,N0.98A)  O1W      - H2W1     .       1.04 Ang.
PLAT480_ALERT_4_C Long H...A H-Bond Reported H6       ..O1       .       2.61 Ang.
PLAT480_ALERT_4_C Long H...A H-Bond Reported H21A     ..O2       .       2.62 Ang.
PLAT480_ALERT_4_C Long H...A H-Bond Reported H23A     ..O8       .       2.61 Ang.
```

Alert level G

```
PLAT002_ALERT_2_G Number of Distance or Angle Restraints on AtSite          2 Note
PLAT007_ALERT_5_G Number of Unrefined Donor-H Atoms ..............          2 Report
PLAT020_ALERT_3_G The Value of Rint is Greater Than 0.12 .........      0.134 Report
PLAT172_ALERT_4_G The CIF-Embedded .res File Contains DFIX Records          1 Report
PLAT395_ALERT_2_G Deviating  X-O-Y    Angle From 120 for O2             109.7 Degree
PLAT398_ALERT_2_G Deviating  C-O-C    Angle From 120 for O7             109.1 Degree
PLAT720_ALERT_4_G Number of Unusual/Non-Standard Labels ..........          5 Note
PLAT791_ALERT_4_G Model has Chirality at C2          (Sohnke SpGr)          S Verify
PLAT791_ALERT_4_G Model has Chirality at C5          (Sohnke SpGr)          R Verify
PLAT791_ALERT_4_G Model has Chirality at C6          (Sohnke SpGr)          R Verify
PLAT791_ALERT_4_G Model has Chirality at C8          (Sohnke SpGr)          R Verify
PLAT791_ALERT_4_G Model has Chirality at C9          (Sohnke SpGr)          S Verify
PLAT791_ALERT_4_G Model has Chirality at C10         (Sohnke SpGr)          R Verify
PLAT791_ALERT_4_G Model has Chirality at C13         (Sohnke SpGr)          S Verify
PLAT791_ALERT_4_G Model has Chirality at C14         (Sohnke SpGr)          R Verify
PLAT791_ALERT_4_G Model has Chirality at C16         (Sohnke SpGr)          S Verify
PLAT791_ALERT_4_G Model has Chirality at C17         (Sohnke SpGr)          R Verify
PLAT791_ALERT_4_G Model has Chirality at C20         (Sohnke SpGr)          S Verify
PLAT791_ALERT_4_G Model has Chirality at C22         (Sohnke SpGr)          R Verify
PLAT791_ALERT_4_G Model has Chirality at C24         (Sohnke SpGr)          R Verify
PLAT791_ALERT_4_G Model has Chirality at C25         (Sohnke SpGr)          S Verify
PLAT860_ALERT_3_G Number of Least-Squares Restraints .............          2 Note
PLAT883_ALERT_1_G No Info/Value for _atom_sites_solution_primary .     Please Do !
PLAT910_ALERT_3_G Missing # of FCF Reflection(s) Below Theta(Min).          1 Note
PLAT912_ALERT_4_G Missing # of FCF Reflections Above STh/L=  0.600          1 Note
PLAT965_ALERT_2_G The SHELXL WEIGHT Optimisation has not Converged     Please Check
PLAT978_ALERT_2_G Number C-C Bonds with Positive Residual Density.          0 Info
```

```
   0 ALERT level A = Most likely a serious problem - resolve or explain
   0 ALERT level B = A potentially serious problem, consider carefully
   9 ALERT level C = Check. Ensure it is not caused by an omission or oversight
  27 ALERT level G = General information/check it is not something unexpected

   1 ALERT type 1 CIF construction/syntax error, inconsistent or missing data
   5 ALERT type 2 Indicator that the structure model may be wrong or deficient
   8 ALERT type 3 Indicator that the structure quality may be low
  21 ALERT type 4 Improvement, methodology, query or suggestion
   1 ALERT type 5 Informative message, check
```

Validation response form

Please find below a validation response form (VRF) that can be filled in and pasted into your CIF.

```
# start Validation Reply Form
_vrf_RINTA01_shelx
;
PROBLEM: The value of Rint is greater than 0.12
RESPONSE: ...
;
_vrf_PLAT057_shelx
;
PROBLEM: Correction for Absorption Required   RT(exp) ...       1.16 Do !
RESPONSE: ...
;
_vrf_PLAT089_shelx
;
PROBLEM: Poor Data / Parameter Ratio (Zmax < 18) ........       6.28 Note
RESPONSE: ...
;
_vrf_PLAT223_shelx
;
PROBLEM: Solv./Anion  Resd 3  H   Ueq(max)/Ueq(min) Range        5.5 Ratio
RESPONSE: ...
;
_vrf_PLAT340_shelx
;
PROBLEM: Low Bond Precision on  C-C Bonds ...............    0.00781 Ang.
RESPONSE: ...
;
_vrf_PLAT355_shelx
;
PROBLEM: Long   O-H (X0.82,N0.98A)  O1W      - H2W1     .       1.04 Ang.
RESPONSE: ...
;
_vrf_PLAT480_shelx
;
PROBLEM: Long H...A H-Bond Reported H6       ..O1       .       2.61 Ang.
RESPONSE: ...
;
# end Validation Reply Form
```

It is advisable to attempt to resolve as many as possible of the alerts in all categories. Often the minor alerts point to easily fixed oversights, errors and omissions in your CIF or refinement strategy, so attention to these fine details can be worthwhile. In order to resolve some of the more serious problems it may be necessary to carry out additional measurements or structure refinements. However, the purpose of your study may justify the reported deviations and the more serious of these should normally be commented upon in the discussion or experimental section of a paper or in the "special_details" fields of the CIF. checkCIF was carefully designed to identify outliers and unusual parameters, but every test has its limitations and alerts that are not important in a particular case may appear. Conversely, the absence of alerts does not guarantee there are no aspects of the results needing attention. It is up to the individual to critically assess their own results and, if necessary, seek expert advice.

Publication of your CIF in IUCr journals

A basic structural check has been run on your CIF. These basic checks will be run on all CIFs submitted for publication in IUCr journals (*Acta Crystallographica*, *Journal of Applied Crystallography*, *Journal of Synchrotron Radiation*); however, if you intend to submit to *Acta Crystallographica Section C* or *E* or *IUCrData*, you should make sure that full publication checks are run on the final version of your CIF prior to submission.

Publication of your CIF in other journals

Please refer to the *Notes for Authors* of the relevant journal for any special instructions relating to CIF submission.

PLATON version of 18/09/2020; check.def file version of 20/08/2020

Datablock shelx - ellipsoid plot

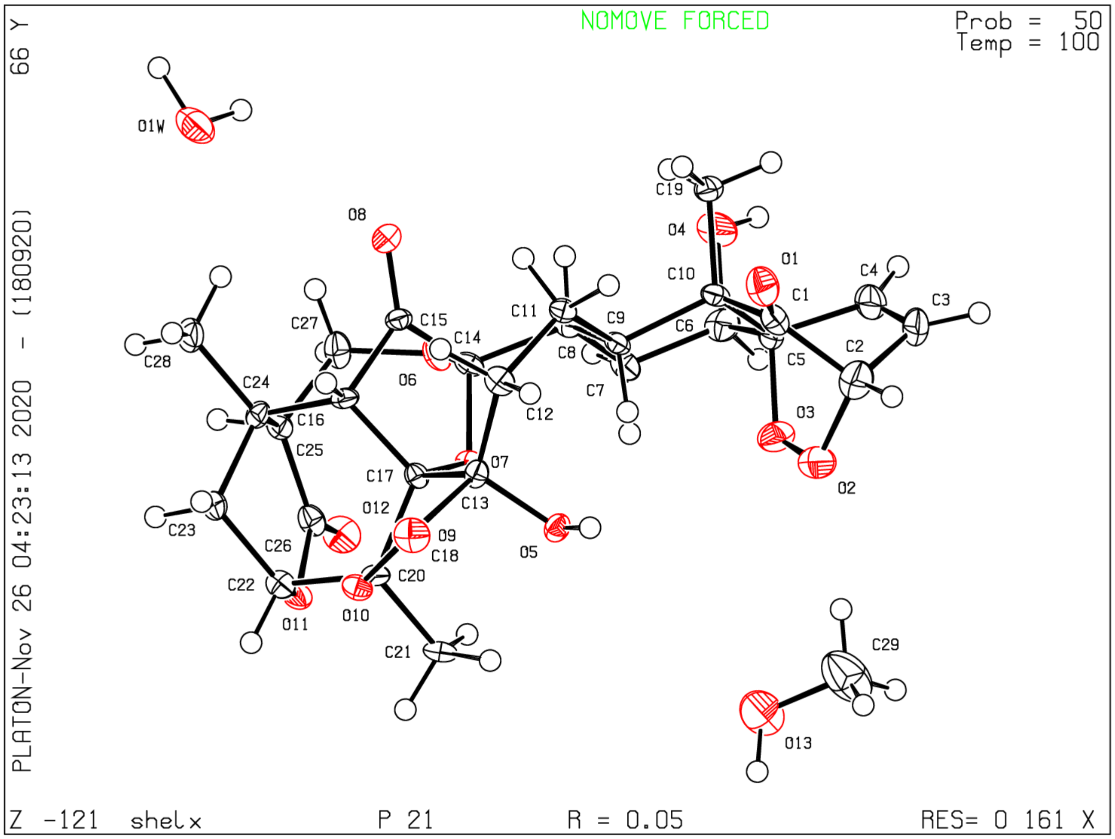

Supplement: S3 File — (PDF) [file pone.0274543.s028.pdf]
