# Supplementary material for: Anti-leishmanial physalins—Phytochemical investigation, in vitro evaluation against clinical and MIL-resistant L. tropica strains and in silico studies
Source: PLoS One. 2022 Nov 28;17(11):e0274543. doi: 10.1371/journal.pone.0274543 (PMC9704608; doi:10.1371/journal.pone.0274543)
Supplement: Attachment — Submitted filename: Physalins-Plos One Word file August 14-2022.docx [file pone.0274543.s029.docx]

**PLOS ONE**

### Anti-leishmanial Physalins - Phytochemcial Investigation, In vitro Evaluation against clinical and MIL Resistant L. tropica strains and In silico Studies

--Manuscript Draft--

| **Manuscript Number:** | PONE-D-22-16415R1 |
| --- | --- |
| **Article Type:** | Research Article |
| **Full Title:** | Anti-leishmanial Physalins - Phytochemcial Investigation, In vitro Evaluation against clinical and MIL Resistant L. tropica strains and In silico Studies |
| **Short Title:** | Anti-leishmanial Physalins from Physalis minima: In vitro Evaluation and In silico Studies |
| **Corresponding Author:** | Sammer Yousuf, Ph. D.  University of Karachi International Center for Chemical and Biological Sciences Karachi, PAKISTAN |
| **Keywords:** | Physalis minima, physalins, leishmaniasis, Leishmania major, Leishmania tropica, |
| **Abstract:** | Cutaneous leishmaniasis (CL) is a major health problem in over 98 countries of the world, including Pakistan. The current treatments are associated with a number of adverse effects and availability problem of drugs. Therefore, there is an urgent need of easily available and cost effective treatments of cutaneous leishmaniasis (CL) in Pakistan. The bioassay-guided fractionation and purification of crude extract of Physalis minima has led to the isolation of a new aminophysalin B ( 1 ), and eight known physalins, physalin B ( 2 ), 5ß,6ß-epoxyphysalin B ( 3 ), 5α-ethoxy-6ß- hydroxy-5,6-dihydrophysalin B ( 4 ), physalin H ( 5 ), 5ß,6ß-epoxyphysalin C ( 6 ), and physalin G ( 7 ), K ( 8 ), and D ( 9 ). It is worth noting that compound 1 is the second member of aminophysalin series, whereas compound 6 was fully characterized for the first time. The structures of compounds 1 - 9 were elucidated by spectroscopic techniques compounds 1 and 8 were also deduced by single- crystal X-ray diffraction studies. Anti-leishmanial activity of isolated physlains 1-9 was evaluated against Leishmania major and Leishmania tropica promastegotes .  Compounds 2 , 3 , and 5 - 7 (IC 50 = 9.59 ± 0.27- 23.76 ± 1.10 μM) showed several fold more potent activity against L. tropca than tested drug miltefosine (IC 50  = 42.75 ± 1.03 μm) and pentamidine (IC 50 = 27.20 ± 0.01 μM). Whereas  compounds 2 , 3 and 5 (IC 50 = 3.04 ± 1.12-3.76 ± 0.85 μM) were found to be potent anti-leishmanial agents against L. major , several fold more active than tested standard miltefosine (IC 50 = 25.55 ± 1.03 μM) and pentamidine (IC 50 = 27.20 ±  0.015 μM ) . Compounds 4 (IC 50 = 74.65 ± 0.81 µM) and 7 (IC 50 = 39.44 ±  0.65 µM) also showed potent antileishmanial ativity against the miltefosine- unresponsive Leishmania tropica strain (MIL resistant) (miltefosine IC 50 = 169.55 ±  0.78 µM). Molecular docking and predictive binding studies indicated that these inhibitors may act via targeting important enzymes of various metabolic pathways of the parasites. |
| **Order of Authors:** | Saira Bano, Ph. D. |
|  | Memona Bibi, M. Phil. |
|  | Saba Farooq, Ph. D. |
|  | Humaira Zafar, Ph. D. |
|  | Muniza Shaikh, Ph. D. |
|  | Behram Khan Khoso, Ph. D. |
|  | M. Iqbal Choudhary, D. Sc. |
|  | Sammer Yousuf, Ph. D. |
| **Response to Reviewers:** | The detailed response file has already been uploaded. |
| **Additional Information:** |  |
| **Question** | **Response** |

| **Financial Disclosure**  Enter a financial disclosure statement that describes the sources of funding for the work included in this submission. Review the [submission guidelines](http://journals.plos.org/plosone/s/submission-guidelines#loc-financial-disclosure-statement) for detailed requirements. View published research articles from [*PLOS ONE*](http://journals.plos.org/plosone/) for specific examples.  This statement is required for submission and **will appear in the published article** if the submission is accepted. Please make sure it is accurate. | “Sammer Yousuf and M. Iqbal Choudhary, researcher leading to these results has, in part, received funding from UK Research and Innovation via the Global Challenges Research Fund under grant agreement ‘A Global Network for Neglected Tropical Diseases’, grant number MR/P027989/1.”  “The funders had no role in study design, data collection and analysis, decision to publish, or preparation of the manuscript.” |
| --- | --- |
| **Unfunded studies**  Enter: *The author(s) received no specific funding for this work.*  **Funded studies**  Enter a statement with the following details:   - Initials of the authors who received each award - Grant numbers awarded to each author - The full name of each funder - URL of each funder website - Did the sponsors or funders play any role in the study design, data collection and analysis, decision to publish, or preparation of the manuscript? - **NO** - Include this sentence at the end of your statement: *The funders had no role in study design, data collection and analysis, decision to publish, or preparation of the manuscript.* - **YES** - Specify the role(s) played. |  |
| * typeset |  |
| **Competing Interests**  Use the instructions below to enter a competing interest statement for this submission. On behalf of all authors, disclose any [competing interests](http://journals.plos.org/plosone/s/competing-interests) that could be perceived to bias this work—acknowledging all financial support and any other relevant financial or non- financial competing interests.  This statement is required for submission | The authors have declared that no competing interests exist. |

| and **will appear in the published article** if the submission is accepted. Please make sure it is accurate and that any funding sources listed in your Funding Information later in the submission form are also declared in your Financial Disclosure statement.  View published research articles from  [*PLOS ONE*](http://journals.plos.org/plosone/) for specific examples. |  |
| --- | --- |
| **NO authors have competing interests**  Enter: *The authors have declared that no competing interests exist.*  **Authors with competing interests**  Enter competing interest details beginning with this statement:  *I have read the journal's policy and the authors of this manuscript have the following competing interests: [insert competing interests here]* |  |
| * typeset |  |
| **Ethics Statement**  Enter an ethics statement for this submission. This statement is required if the study involved:   - Human participants - Human specimens or tissue - Vertebrate animals or cephalopods - Vertebrate embryos or tissues - Field research   Write "N/A" if the submission does not require an ethics statement.  General guidance is provided below. Consult the [submission guidelines](http://journals.plos.org/plosone/s/submission-guidelines#loc-guidelines-for-specific-study-types) for detailed instructions. **Make sure that all information entered here is included in the Methods section of the manuscript.** | Nil |

| **Format for specific study types**  **Human Subject Research (involving human participants and/or tissue)**   - Give the name of the institutional review board or ethics committee that approved the study - Include the approval number and/or a statement indicating approval of this research - Indicate the form of consent obtained (written/oral) or the reason that consent was not obtained (e.g. the data were analyzed anonymously)   **Animal Research (involving vertebrate animals, embryos or tissues)**   - Provide the name of the Institutional Animal Care and Use Committee (IACUC) or other relevant ethics board that reviewed the study protocol, and indicate whether they approved this research or granted a formal waiver of ethical approval - Include an approval number if one was obtained - If the study involved *non-human primates*, add *additional details* about animal welfare and steps taken to ameliorate suffering - If anesthesia, euthanasia, or any kind of animal sacrifice is part of the study, include briefly which substances and/or methods were applied   **Field Research**  Include the following details if this study involves the collection of plant, animal, or other materials from a natural setting:   - Field permit number - Name of the institution or relevant body that granted permission |  |
| --- | --- |
|  |  |
| **Data Availability**  Authors are required to make all data underlying the findings described fully available, without restriction, and from the time of publication. PLOS allows rare exceptions to address legal and ethical concerns. See the [PLOS Data Policy](http://journals.plos.org/plosone/s/data-availability) and [FAQ](http://journals.plos.org/plosone/s/data-availability#loc-faqs-for-data-policy) for detailed information. | Yes - all data are fully available without restriction |

| A Data Availability Statement describing where the data can be found is required at submission. Your answers to this question constitute the Data Availability Statement and **will be published in the article**, if accepted. |  |
| --- | --- |
| **Important:** Stating ‘data available on request from the author’ is not sufficient. If your data are only available upon request, select ‘No’ for the first question and explain your exceptional situation in the text box. |  |
| Do the authors confirm that all data underlying the findings described in their manuscript are fully available without restriction? |  |
| **Describe where the data may be found in full sentences. If you are copying our sample text, replace any instances of XXX with the appropriate details.**   - If the data are **held or will be held in a public repository**, include URLs, accession numbers or DOIs. If this information will only be available after acceptance, indicate this by ticking the box below. For example: *All XXX files are available from the XXX database (accession number(s) XXX, XXX.*). - If the data are all contained **within the manuscript and/or Supporting Information files**, enter the following: *All relevant data are within the manuscript and its Supporting Information files.* - If neither of these applies but you are able to provide **details of access elsewhere**, with or without limitations, please do so. For example:   *Data cannot be shared publicly because of [XXX]. Data are available from the XXX Institutional Data Access / Ethics Committee (contact via XXX) for researchers who meet the criteria for access to confidential data.*  *The data underlying the results presented in the study are available from (include the name of the third party* | All relevant data are within the manuscript and its Supporting Information files. |

| *and contact information or URL).*   - This text is appropriate if the data are owned by a third party and authors do not have permission to share the data.   * typeset |  |
| --- | --- |
| Additional data availability information: | Tick here if your circumstances are not covered by the questions above and you need the journal’s help to make your data available. |

<https://www.editorialmanager.com/pone/download.aspx?id=31654354&guid=adad1d48-a1ca-4444-9d22-3459c97a39f7&scheme=1>Manuscript	[Click here to access/download;Manuscript;Manuscript Final- Clean_copy.docx](https://www.editorialmanager.com/pone/download.aspx?id=31654354&guid=adad1d48-a1ca-4444-9d22-3459c97a39f7&scheme=1)

## 1 Anti-leishmanial physalins - phytochemical investigation, *in vitro*

2 **evaluation against clinical and MIL-resistant *L. tropica* strains and**
3 ***in silico* studies**

###### 4 Saira Bano^1^, Memoona Bibi^1^, Saba Farooq^2^, Humaira Zafar^2^, Muniza Shaikh^2^, Behram

5 **Khan Khoso**^3^**, SammerYousuf***^1^**, M. Iqbal Choudhary^*^**^1,^ 2, 4
6 ^1^H.E.J. Research Institute of Chemistry, International Center for Chemical and Biological
7 Sciences, University of Karachi, Karachi-75270, Pakistan
8 ^2^ Dr. Panjwani Center for Molecular Medicine and Drug Research, International Center for
9 Chemical and Biological Sciences, University of Karachi, Karachi-75270, Pakistan
10 ^3^Jinnah Post-Graduate Medical Center, Sarwar Shaheed Rd, Karachi Cantonment, Karachi-
11 75510, Pakistan
12 ^4^Department of Biochemistry, Faculty of Science, King Abdulaziz University, Jeddah-21412,
13 Saudi Arabia

14

15

16

17

18

19

20

21

22

23

24 ***Corresponding author:** [dr.sammer.yousuf@gmail.com](mailto:dr.sammer.yousuf@gmail.com) (SY)
25 ***Additional corresponding author:** [iqbal.choudhary@iccs.edu](mailto:iqbal.choudhary@iccs.edu) (MIC)

# 26 Abstract

27 Cutaneous leishmaniasis (CL) is a major health problem in over 98 countries of the world,
28 including Pakistan. The current treatments are associated with a number of adverse effects and
29 availability problem of drugs. Therefore, there is an urgent need for easily available and cost
30 effective treatments of cutaneous leishmaniasis (CL) in Pakistan. The bioassay-guided
31 fractionation and purification of crude extract of *Physalis minima* has led to the isolation of a
32 new aminophysalin B (**1**), and eight known physalins, physalin B (**2**), 5ß,6ß-epoxyphysalin B
33 (**3**), 5α-ethoxy-6ß-hydroxy-5,6-dihydrophysalin B (**4**), physalin H (**5**), 5ß,6ß-epoxyphysalin C
34 (**6**), and physalin G (**7**), K (**8**), and D (**9**). It is worth noting that compound **1** is the second
35 member of aminophysalin series, whereas compound **6** was fully characterized for the first
36 time. The structures of compounds **1**-**9** were elucidated by spectroscopic techniques Whereas,
37 the structural assignments of compounds **1** and **8** were also supported by single-crystal X-ray
38 diffraction studies. The anti-leishmanial activity of isolated physlains **1-9** was evaluated against
39 *Leishmania major* and *Leishmania tropica* promastigotes*.* Compounds **2**, **3**, and **5**-**7** (IC**50 =**
40 9.59 ± 0.27- 23.76 ± 1.10 μM) showed several-fold more potent activity against *L. tropica* than
41 tested drug miltefosine (IC**50 =** 42.75 ± 1.03 μm) and pentamidine (IC50 = 27.20 ± 0.01 μM).
42 Whereas compounds **2**, **3** and **5** (IC**50 =** 3.04 ± 1.12-3.76 ± 0.85 μM) were found to be potent
43 anti-leishmanial agents against *L. major*, severalfolds more active than tested standard
44 miltefosine (IC**50 =** 25.55 ± 1.03 μM) and pentamidine (IC**50 =** 27.20 ± 0.015 μM**)**. Compounds
45 **4** (IC50 = 74.65 ± 0.81 µM) and **7** (IC50 = 39.44 ± 0.65 µM) also showed potent antileishmanial
46 activity against the miltefosine-unresponsive *Leishmania tropica* strain (MIL resistant)
47 (miltefosine IC50 = 169.55 ± 0.78 µM). Molecular docking and predictive binding studies
48 indicated that these inhibitors may act *via* targeting important enzymes of various metabolic
49 pathways of the parasites.
50 **Key Words:** *Physalis minima,* physalins, leishmaniasis*, Leishmania major*, *Leishmania*
51 *tropica,*

# 52 Introduction

53 Cutaneous leishmaniasis (CL), a neglected tropical disease (NTD), infects 700,000 to 1.2
54 million poorest people in over 98 countries of Africa, Asia, the Middle East, and America annually.
55 Poverty, migration, poor hygiene, and malnutrition are the major risk factors associated with
56 endemicity of various forms of leishmaniasis in the developing world [1-3]. About 70 types of
57 sand flies are known to be responsible to transmit the *Leishmania* parasite geographically
58 distributed Old (Asia, Africa, and the Middle East) and New world *i.e.* western hemisphere
59 countries. Broadly they belong to the phlebotomine and *Lutzomyi*a genera. The diagnosis of
60 leishmaniasis, both parasitic (direct) and immunological (indirect), is often challenging in
61 low-resource countries [1]. Pakistan is among the endemic countries with CL cases in almost
62 every part of the country. In 2020, as per WHO Global Leishmaniasis Surveillance Report,
63 Afghanistan, Algeria, Brazil, Colombia, Iraq, Pakistan, and Syria, are the seven countries, each
64 reported >6,000 CL cases, representing >80% of cases globally [4]. CL is not a life-threatening
65 infection but cause extreme psychological trauma, and stigma as infected people pushed to be
66 excluded from public life.
67 The available therapeutic options are now losing effectiveness due to the emergence of
68 resistance [5, 6]. Furthermore, these available drugs are expensive, and some have toxic effects
69 on liver and kidneys [7]. Medicinal plants have immense potential to yield compounds that are
70 capable of treating many ailments. In recent days, pharmaceutical industries have reformed
71 their attention towards plant-based medicine and formulation because of their least toxicity
72 and more efficacy [8-10]. *Physalis minima* Linn is a member of the *Solanaceae* family, commonly
73 known as native gooseberry, and reported for anti-cancer, anti-diabetic, anti-oxidant, anti-
74 ulcer, analgesic, antipyretic, inhibition of amylase, lipase, and alpha-glucosidase enzymes, as
75 well as anti-gonorrhoeal, and anti-inflammatory properties [11-15]. Previous research on *P.*
76 *minima* led to the isolation of bioactive withanolides and physalins, which showed potent anti-
77 inflammatory and cytotoxic activities [16-19]. We have reported the *in vitro* anti-leishmanial
78 activity of the *Physalis minima* plant extracts and its pure constituents physalin B (**2**), 5ß,6ß-
79 epoxyphysalin B (**3**), and physalin H (**5**) against promastigotes of *L. major* for the first time
80 [20-22]. The presented study deals with the isolation, structure determination, and anti-
81 leishmanial effect of a new aminophysalin B (**1**) along with eight known physalins, physalin
82 B (**2**), 5ß,6ß-epoxyphysalin B (**3**), 5α-ethoxy-6ß-hydroxy-5,6-dihydrophysalin B (**4**), physalin
83 H (**5**), 5ß,6ß-epoxyphysalin C (**6**), and physalin G (**7**), K (**8**), and D (**9**) *in vitro* against
84 promastegotes of *L. tropica*. Compounds **1, 4**, **6**, **7, 8,** and **9** were also evaluated against
85 promastigotes of *L. major* but no significant activity was observed. Moreover, the effect of
86 compounds **2-9** were also evaluated for their potential against the MIL-resistant *Leishmania*
87 *tropica*. These steroidal lactones **1**-**9** were also subjected to in-silico studies to predict their
88 mechanisms of action.

# 89 Materials and Methods

## 90 General

91 Precoated TLC plates (silica gel, 20×20, 0.25 mm thick PF254, Merck, Germany) were used for
92 thin layer chromatography, and stained by spraying with Dragendroff’s and vanillin reagents.
93 Column chromatography was carried out on silica gel (70–230 mesh, Merck, Germany) [23].
94 Recycling preparative separation was performed on an HPLC JAI LC-908W (Japan) instrument,
95 equipped with GAIGEL-SIL, D-60-10, CHCl3-MeOH as the mobile phase, with UV detection
96 at 254 nm. JMS-600H mass spectrometer (JEOL, Japan) was used to record the mass spectra
97 (EI- and HREI-MS) in *m/z* (rel. %). Electrospray ionization mass spectra (ESI-MS) were
98 recorded on Amazon speed ion trap low-resolution mass spectrometer (Bruker
99 Daltonics, Germany) and high-resolution electrospray ionization mass spectra (HRESI-MS)
100 were	measured	on	MaXis	II	ESI–QTOF	Ultra	High-Resolution	Mass
101 Spectrometer (Bruker Daltonics, Germany). Ultraviolet (UV) experiments were performed
102 in methanol on Hitachi U-3200 spectrophotometer (Hitachi, Japan). Infrared (IR) analysis was
103 performed	in	KBr	on	FT-IR-8900	(Shimadzu,	Japan),	and	Bruker	VECTOR	22
104 spectrophotometers (Bruker, France). UV Spectra (nm) were measured in methanol and
105 chloroform on Evolution 300 UV visible spectrophotometer. FT-IR-8900 spectrophotometer
106 was used to record IR spectra (cm^-1^) (JASCO, Japan). Optical rotations were measured in
107 methanol with a digital polarimeter JASCO P-2000 (JASCO, Japan) by using a 10 cm cell tube.
108 ^1^H- and ^13^C-NMR spectra were recorded on Avance NMR spectrometer at 500 and 75–150
109 MHz in CDCl3 or CD3OD, respectively (Bruker, Zurich, Switzerland). Standard pulse
110 sequences were used for DEPT, and 2D-NMR experiments. The ^1^H-NMR, ^13^C-NMR, COSY,
111 HMBC, HMQC, and NOESY spectra were recorded in deuterated chloroform and methanol
112 on Bruker Avance spectrometers (Bruker, Zurich, Switzerland). X-Ray crystallography data
113 were collected on Bruker D8 venture diffractometer. Cu Kα radiation (1.54178 Å) was used
114 for X-ray diffraction data collection. SAINT program was employed for data integration and
115 reduction; Direct method was utilized for structure solution with full-matrix least square
116 the method with aid of SHELXTL (SHELX2016/6).,

## 117 Plant Material

118 Fresh plants (50 kg.) of *Physalin minima* (L. Var. indica), were collected from Karachi,
119 Pakistan, and identified by a plant taxonomists, Department of Botany, University of Karachi.
120 A voucher specimen (G. H. 68261) was deposited at the herbarium.
121 **Extraction and Isolation**
122 The air-dried plants (11 kg) were extracted with EtOH (100 L) for 10 days. After evaporation
123 of the solvent, a brownish residue (1.7 kg) was obtained that was allowed to be suspended in
124 distilled H2O (5 L), and defatted with petroleum ether (10 L) [24]. The defatted aqueous extract
125 was further extracted with CH2Cl2 (35 L). The CH2Cl2 extract was concentrated under reduced
126 pressure to obtain a greenish dried powder (85 g) that was subjected to column chromatography
127 and eluted with Pet ether: CH2Cl2, CH2Cl2, CH2Cl2: MeOH (10:90…90:10, 100, 99:1-97:3) to
128 afford four main fractions **A** (2.14 **g**), **B** (1.54 g), **C** (1.14 g), and **D** (24 g), respectively. The
129 repeated column chromatography of fraction **A** (2.14 **g**) afforded one major fraction **a** (300 mg,
130 petroleum: acetone 80: 20) which was further purified on recycling reverse phase HPLC to
131 obtain purified compound **2** (10 mg, CHCl3: MeOH-99:1, Flow rate: 4 mL/min, RT=16 min)
132 and **3** (60 mg, CHCl3: MeOH-99:1, Flow rate: 4 mL /min, RT = 18min). Fraction **B** (1.54 **g**)
133 yielded subfraction **b** (500 mg, petroleum: acetone 70: 30) which was further subjected to
134 recycling reverse phase HPLC to obtain compound **5** (10 mg, CHCl3: MeOH-99:1, Flow rate:
135 4 mL/min, RT=16 min). Fraction **C** (1.14 **g**) afforded one major subfraction **c** (petroleum:
136 acetone 80: 20) which yielded and purified compound **6** (5 mg, CHCl3: MeOH-99:1, Flow rate:
137 4 mL/min, RT=16 min) *via* recycling reverse phase HPLC. Fraction **D** (24 **g**) was subjected to
138 column chromatography and three major subfractions were obtained. Subtraction **d** (1.41)
139 (petroleum ether: acetone-55:45), **e** (1.0) (Petroleum ether: acetone-45:55) and **f** (20 g)
140 (petroleum: acetone-20:80) which were purified by using recycling reverse phase HPLC to
141 obtain compounds **1** (3 mg, CHCl3: MeOH-97:3, Flow rate: 3 mL/min, RT= 24 min) and **4** (4
142 mg, CHCl3: MeOH-96:4, Flow rate: 4 mL/min, RT= 24 min), **7** (10mg, CHCl3: MeOH-97:3,
143 Flow rate: 3 mL/min, RT= 30 min), **8** (6 mg, CHCl3: MeOH-97:3, Flow rate: 3 mL/min, RT=
144 24 min), and **9** (6 mg, CHCl3: MeOH-97:3, Flow rate: 3 mL/min, RT= 32 min.

### 145 Aminophysalin B (1)

| 146 | Colorless solid (3 mg): [α] D= -65 (c = 0 .01, MeOH), **UV** (MeOH) λmax nm (log∈): 312 (3.2). |
| --- | --- |
| 147 | **IR** (KBr) νmax cm^-1^: 3463 (OH), 1732 (C=O), 1656 (C=C-C=O), **^1^H-NMR** (CDCl3, 500 MHz): |
| 148 | Table 1. **^13^C-NMR** (CDCl3, 175 MHz): Table 1. **HRESI-MS** *m/z*: 526.2059 |
| 149 | [M+H]^+^(C24H31NO9+H requires 526.2072). |
| 150 | **Single-Crystal X-Ray Diffraction Data:** Empirical formula= C28 H31 NO9. H2O, Mr = 543.55, |
| 151 | Crystal system: orthorhombic, space group: P212121, Unit cell dimensions: **a** = 9.7870(4) Å, **b** |
| 152 | = 9.7870(4) Å, **c** = 16.3581(7) Å, Volume: 2412.66(18) Å^3^, Z=4, ρcalc = 1.496 Mg/m3, F(000) |
| 153 | 1152, Crystal size: 0.900 x 0.060 x 0.030 mm, θ range for data collection: 5.266 to 68.135 deg. |
| 154 | Total 15,452 reflections were collected, out of which 4,364 were found unique (Rint= 0.0945). |
| 155 | Final R indices were R1 = 0.0551for [I>2σ (I)], wR2 = 0.1446, R indices were R1 = 0.0699, |
| 156 | wR2 = 0.1547 for all data, largest diff. peak and hole: 0.434 and -0.369 e. Å^-3^, CCDC No. |
| 157 | 2177422. |
| 158 | **Physalin B** (**2**) |
| 159 | Colorless solid (10 mg): [α] D= -257 (c = 0 .05, MeOH), **UV** (MeOH) λmax nm (log ∈): 230 |
| 160 | (3.6). **IR** (KBr) νmax cm^-1^ : 3410 (OH), 1748 (lactone), 1653 (C=C-C=O), **^1^H-NMR** (CDCl3, |
| 161 | 500 MHz): 1.19 (s, H3-28), 1.25 (s , H3-19), 1.95 (s, H3-21), 4.35 (t, overlapped *J*22,23 = 2.5 Hz, |
| 162 | H-22), 3.75 (d, *J*27,27 = 12.5 Hz, H2-27), 4.50 (dd overlapped, *J*27,27 = 13.5 Hz, *J*27,25 = 4.5, H2- |
| 163 | 27), 5.91 (dd, *J*2,3 = 10.0 Hz, *J*2, 4ß = 2 Hz, H-2), 6.77 (ddd, *J*3,2 = 10.0 Hz, *J*3,4a = 5.0 Hz, *J*3,4ß = |
| 164 | 2.5, H-3), 5.56 (d, *J*6,7 = 6.0 Hz, H-6), EIMS *m/z* (rel.int. %): 510 (M^+^), 492 (100.0), 477 (21.7), |
| 165 | 464 (48.0), 173 (46.2), 159 (70.9), 145 (26.1), 91 (26.6), 43 (18.1). |
| 166 | **5*β*, 6*β*-Epoxyphysalin B** (**3**) |
| 167 | White solid (60 mg), [α] D= -108 (*c* = 0 .05, MeOH), **UV** (MeOH) λmax nm (log ∈) : 230 (3.5). |
| 168 | **IR** (KBr) νmax cm^-1^ : 3422 (OH), 1778 (lactone), 1656 (C=C-C=O), **^1^H-NMR** (CDCl3, 500 |
| 169 | MHz): 1.24 (s , H3-28), 1.29 (s, H3-19), 1.93 (s, H3-21), 3.25 (d, *J*6α, 7β = 3.0 Hz, H-6), 4.52 (t, |

| 170 | overlapped *J*22,23 = 1.5 Hz, H-22), 3.74 (d, *J*27,27 = 13.5 Hz, H-27), 4.50 (dd overlapped, *J*27,27 = | | | | | |
| --- | --- | --- | --- | --- | --- | --- |
| 171 | 14.0 Hz, *J*27,25 = 4.5 Hz, H-27), 5.99 (dd, *J*2,3 = 10.5 Hz, *J*2, 4ß = 2.0 Hz, H-2), 6.84 (ddd, *J*3,2 = | | | | | |
| 172 | 10.0 Hz, *J*3,4α= 5.0 Hz, *J*3,4ß = 2.5 Hz, H-3), EI-MS *m/z* (rel. int. %): 526 (6.7, M^+^), 498 (100.0), | | | | | |
| 173 | 480 (15.7), 360 (16.6), 159 (17. 0), 133 (20.6), 91 (14.6), 55 (15.0). | | | | | |
| 174 | **5*α*-Ethoxy-6ß-hydroxy-5,6-dihydrophysalin B** (**4**) | | | | | |
| 175 | Colorless solid (4 mg): [α] D = -58 (*c* = 0 .05, MeOH), **UV** (MeOH) λmax nm (log ∈) : 230 (3.2). | | | | | |
| 176 | **IR** (KBr) νmax cm^-1^ : 3405 (OH), 1787 (lactone), 1667 (C=C-C=O), **^1^H-NMR** (CDCl3, 500 | | | | | |
| 177 | MHz): 0.98 (t, *J =* 7 Hz, CH3CH2O), 1.24 (s, H3-28), 1.50 (s, H3-19), 1.98 (s, H3-21), 4.00 (m, | | | | | |
| 178 | H-6), 4.54 (t, overlapped *J*22,23 = 1.5, H-22), 3.76 (d, *J*27,27 = 13.5 Hz, H-27),4.49 (dd, *J*27,25 = | | | | | |
| 179 | 4.5 Hz H-27), 5.85 (dd, *J*2,3 = 10.0 Hz, *J*2, 4ß = 2.5 Hz , H-2), 6.57 (ddd, *J*3,2 = 10.0 Hz, *J*3,4α = | | | | | |
| 180 | 5.0 Hz, *J*3,4ß = 2.0 Hz, H-3), EI-MS *m/z* (rel. int. %): 572 (7.4, M^+^), 544 (10.8), 526 (24.8), 508 | | | | | |
| 181 | (60.1), 454 (30.3), 171 (79.3), 147 (77.1), 133 (100.0), 109 (89.9), 91 (64.3), 55 (75.6). | | | | | |
| 182 | **Physalin H** (**5**) | | | | | |
| 183 | Colorless solid (10 mg): [α] D = -260 (*c* = 0 .01, MeOH), **UV** (MeOH) λmax nm (log ∈) : 230 | | | | | |
| 184 | (3.8). **IR** (KBr) νmax cm^-1^ : 3426 (OH), 1778 (lactone), 1673 (C=C-C=O), **^1^H-NMR** (CDCl3, | | | | | |
| 185 | 500 MHz):1.35 (s, H3-19), 1.25 (s, H3-28), 1.99 (s, H3-21), 4.10 (d, *J*6α, 7β = 3.0 Hz, H-6), 4.54 | | | | | |
| 186 | (t, overlapped *J*22,23 = 2.5 Hz, H-22), 3.75 (d, *J*27,27 = 12.5 Hz, H2-27), 4.50 (dd, overlapped, | | | | | |
| 187 | *J*27,27 = 13.5 Hz, *J*27,25 = 4.5 Hz, H2-27), 5.94 (dd, *J*2,3 = 10.0 Hz, *J*2, 4ß = 2.0 Hz, H-2), 6.71 (ddd, | | | | | |
| 188 | *J*3,2 | = 10.0 Hz, | *J*3,4α= | 5.0 Hz, | *J*3,4ß | = 2.5 Hz, H-4) **HRESI-MS** m/z: 563.1674 [M+H]^+^ |
| 189 | (C28H31ClO10+H requires 563.1679). | | | | | |
| 190 | **5*β*, 6*β*-Epoxyphysalin C** (**6**) | | | | | |
| 191 | White solid (5 mg): [α] D= -168 (c = 0 .01, MeOH), **UV** (MeOH) λmax nm (log ∈) : 230 (3.1). | | | | | |
| 192 | **IR** (KBr) νmax cm^-1^: 3427 (OH), 1782 (lactone), 1656 (C=C-C=O), **^1^H-NMR** (CH3OD, 500 | | | | | |

| 193 | MHz): Table 1. **^13^C-NMR** (CH3OD, | | 175 MHz): Table 1. **HRESI-MS** *m/z*: 527.1907 |
| --- | --- | --- | --- |
| 194 | [C29H30O10+H; 527.1912] |  |  |
| 195 | **Physalin G** (**7**) |  |  |

196	White solid (10 mg): [α] D= -92 (*c* = 0 .01, MeOH), **UV** (MeOH) λmax nm (log ∈) : 312 (5.8).**IR**

| 197 | (KBr) | νmax | cm-1 | : 3418 (OH), 1776 (lactone), 1622 (C=C-C=O), **^1^H-NMR** | (CDCl3, | 500 |
| --- | --- | --- | --- | --- | --- | --- |
| 198 | MHz):1.25 (s, H3-28), 1.93 (s, H3-19), 1.51 (s, H3-21), 4.49 (m, H-6), 4.52 (m, H-22), 3.74 | | | | | |
| 199 | (*J*27,27 = 13.0 Hz, H2-27), 4.50 (m, H2-27), 5.93 (d, *J*2,3 = 5.5 Hz, H-2), 6.82 (dd, *J*3,2 = 9.5 Hz, | | | | | |
| 200 | *J*3,4 = 5.5 Hz, H-4), 6.03 (d, *J*4,3 = 6.0 Hz), **EIMS** *m/z* **(**rel. int. %): 526 (2.8, M^+^), 508 (55.1), | | | | | |
| 201 | 482 (79.8), 454 (40.1), 185 (38.9), 159 (27.6), 159 (98.6), 134 (67.1) 109 (43.5), 91 (27.0), 55 | | | | | |
| 202 | (32.4). | | | | | |
| 203 | **Physalin K** (**8**) | | | | | |
| 204 | Colorless solid (6 mg): [α] D= -266 (c = 0 .01, MeOH), **UV** (MeOH) λmax nm (log ∈) : 229 | | | | | |
| 205 | (3.0). **IR**(KBr) ν max cm^-1^ : 3407 (OH), 1775 (lactone), 1630 (C=C-C=O), **^1^H-NMR** (CDCl3, | | | | | |
| 206 | 500 MHz):1.12 (s , H3-28), 1.20 (s, H3-19), 1.90 (s, H3-21), 3.90 (d, *J*6α, 7β = 3.0, H-6), 4.55 (m, | | | | | |
| 207 | H-22), 3.76 (d, *J*27,27 = 13.5 Hz, H2-27), 4.49 (dd, *J*22,23α = 13.5 Hz, *J*22,23β = 5.0 Hz, H-22), 6.65 | | | | | |
| 208 | (dd, *J*3,2 = 6.5 Hz, *J*3,4 = 8.0 Hz, H-3), 7.00 (dd, *J*4,2= 1.0 Hz, *J*4,3= 8.5 Hz, H-4), **HRESI-MS** | | | | | |
| 209 | *m/z*: 559.1800 [C28H30O12+H; 559.1810]. | | | | | |
| 210 | **Single-Crystal X-Ray Diffraction Data:** Empirical formula= C28 H30 O12. H2O. CH3OH, Mr | | | | | |
| 211 | = 358.46, Crystal system: Monoclinic, space group: P21, Unit cell dimensions: **a** = 7.4821(5)Å, | | | | | |
| 212 | **b** = 11.7099(7)Å, **c** = 15.2299(11)Å, Volume: 1327.52(15)Å^3^, Z=2, ρcalc = 1.522 Mg/m3, | | | | | |
| 213 | F(000) 776, Crystal size: 0.270 x 0.130 x 0.010 mm, θ range for data collection: 4.773 to 68.237 | | | | | |
| 214 | deg. A total of 17,151 reflections collected, out of which 4,824 reflections were judged | | | | | |
| 215 | observed (Rint = 0.1337), Final R indices were, R1 = 0.0521for [I>2σ (I)], wR2 = 0.1156, R | | | | | |
| 216 | indices were R1 = 0.0816, wR2 = 0.1300 for all data, largest diff. peak and hole: 0.349and -- | | | | | |
| 217 | 0.269e. Å^-3^, CCDC No. 2177423. | | | | | |

### 218 Physalin D (9)

219 Colorless solid (6 mg): [α] D= +78.4 (c = 0 .01, MeOH), **UV** (MeOH) λmax nm (log ∈) : 230

220	(3.2).**IR** (KBr) νmax cm^-1^: 3377 (OH), 1788 (lactone), 1653(C=C-C=O),**^1^H-NMR** (CDCl3, 500

221	MHz):1.25 (s, H3-28),1.29 (s, H3-28), 1.98 (s, H3-28), 3.76 (m, H-6), 4.53 (m, H-22), 3.74 (m,

222	H2-27), 4.50 (d, *J*27,25= 5.0 Hz, H2-27), 6.65 (ddd, *J*3,2 = 10.5, Hz, *J*3,4a = 4.5 Hz, *J*3,4ß = 2.5, H-

223	3), 5.90 (dd, *J*2,3 = 10.5 Hz, *J*2, 4ß = 2.5 Hz, H-2), **HRESI-MS** [M+H]^+^ *m/z*: 545.2013

224	[C28H32O11+H; 545.2004].

## 225 Promastigote Growth Inhibition Assay

226 For the estimation of particular concertation at which any compounds caused 50%
227 inhibition (IC50) of resistant cell proliferation concerning untreated controls, the MTT assay
228 was employed [25]. The effects of compounds against MIL-resistant line, the promastigotes
229 in their log phase were used in 96-well plates. 1 × 10^6^ resistant promastigotes were dispensed
230 and incubated with tested compounds at a range concentration of 200-10 μM at 27°C for 72 h.
231 After incubation of 72 h, MTT dye (3-(4,5-dimethylthiazol-2-yl)-2,5-diphenyl-2H-tetrazolium
232 bromide) was added and further incubate for 3-4 h. amphotericin B was used as the positive
233 control, untreated promastigotes were used as negative control moreover DMSO control was
234 also added in the study as a stock solution of the compounds that were prepared in DMSO. All the
235 experiments were performed in triplicates. After completion of the experiment the absorbance by
236 Multiskan ascent plate reader at 452 nm and percent inhibition was calculated by the following
237 formula
238 Cytotoxic concentration (%) = [100- (Absorbance of test)/ (Absorbance of solvent control)

239	×100] %

### 240 MIL Resistant Line

241 Promastigote cultures *Leishmania tropica* (Clinical isolates) were maintained at 26 °C in
242 RPMI-1640 medium containing 15% FBS and 1% penicillin-streptomycin mixture.
243 Generation of miltefosine-unresponsive strain was carried under high MIL pressure by *in vitro*
244 passage with a stepwise increase in the MIL concentration. At each step, parasites were cultured
245 and passaged every 3–4 days at an initial concentration of 5x10^5^ promastigotes/mL in order to
246 achieve stable growth as compared to the wild-type isolate. Growth rates were measured
247 for resistant populations and compared with the WT strain. Parasites were counted at an initial
248 the concentration of 5x10^5^ parasites/mL and growth was measured daily using a Neubauer
249 chamber until the population reached the stationary phase. Furthermore, the fluorescence
250 microscopic investigations via DAPI stain were also carried out to supplement the study [26,

251	27].

### 252 Cytotoxicity against 3T3 Cell Lines

253 The cytotoxicity of the test compounds against cancer and normal cell lines was evaluated
254 through	MTT	(3-[4,	5-dimethylthiazole-2-yl]-2,	5-diphenyl-tetrazolium	bromide]
255 colorimetric assay in 96-well flat-bottomed microplates [28]. The 3T3 (mouse fibroblast) were
256 cultured Dulbecco’s Modified Eagle Medium, supplemented with 5% of fetal bovine serum
257 (FBS), 100 IU/mL of penicillin and 100 µg/mL of streptomycin in 75 cm2 flasks, and then
258 incubated in a 5% CO2 incubator at 37ºC. The growing cells were counted by using a
259 hemocytometer, and diluted with the particular medium. The 5x10^4^ cells/mL of the cell culture
260 was prepared, and a sample (100 mL) was introduced into each well. The medium was removed after
261 overnight incubation and 200 µ L of fresh medium was added with different concentrations of
262 compounds (1-30 µM). After 48 hours, 200 µL MTT (0.5 mg/mL) was added, and the
263 incubation was further continued for 4 hrs. After that time, 100 µL of DMSO was added to
264 each well. The absorbance was measured using a microplate reader (Spectra Max Plus,
265 Molecular Devices, CA, USA) at 570 nm for the extent of MTT reduction to formazan within
266 the cells. The cytotoxicity was recorded as IC50 against cells by using SoftMax Pro software.
267 Doxorubicin and cycloheximide were used as a positive control. The experiments were run in
268 triplicate.

## 269 Docking Studies

270 In order to predict the tentative binding affinities and the possible mode of inhibition *via* these
271 compounds, molecular docking studies against various leishmanial metabolic enzymes that
272 have a role in the growth and survival of the parasite were conducted.

### 273 Protein Preparation

274 For docking studies, the selected protein targets *i. e.,* pteridine reductase (PDB:1E92), pyruvate
275 kinase (PDB:3PP7), glyceraldehyde-3-phosphate dehydrogenase (PDB:1I32), phosphoglucose
276 isomerase	(PDB:1T10),	dihydroortate	dehydrogenase	(PDB:4EF8),	fructose-1,6-
277 bisphosphatase (PDB:5OFU), SAM-dependent methyltransferase (PDB:1XTP), mitochondrial
278 fumarate hydratase (PDB: 6MSO), *N*-myristoyl transferase (PDB: 6QDB), and MAP kinase
279 (PDB: 3UIB), were prepared by *Protein Preparation Wizard* in Maestro Schrödinger 12 [29,
280 30]. During preparation, the missing hydrogens were added, and partial charges were assigned
281 using OPLS-3e force field. Hydrogens and heavy atoms were optimized by restrained
282 minimization.

### 283 Ligand Preparation and Database Generation

284 The 2D structures of compounds were converted to 3D structures, *via* the Ligprep module in
285 Maestro Schrödinger 12 [31]. Ligprep was used to correct the protonation, and ionization states
286 of the compounds, and assigned proper bond orders. Afterward, the tautomeric and ionization
287 states were created for each ligand.

### 288 Receptor Grid Generation and Molecular Docking

289 The grid box was defined by selecting the co-crystallized molecules in the binding site of
290 abovementioned selected protein targets to keep the center of each docked ligand with the same
291 dimensions of the binding box. Rigid receptor docking protocol was run in standard precision (SP)
292 mode of Glide based on OPLS-3e force field [32-34]. During the process of docking, the
293 protein was fixed, while ligands were flexible.
294 The molecular mechanics-generalized Born surface area (MM-GBSA) method in Prime was used
295 for rescoring the docked pose of ligand in the binding site of the selected target protein [35]. These
296 poses were taken as inputs for the energy minimization of the protein-ligand complexes
297 (Ecomplex), free protein (Eprotein), and free ligands (Eligand). The binding free energy ∆Gbind was
298 determined according to the following equation:

299	𝛥𝐺𝑏𝑖𝑛𝑑 = 𝐸𝑐𝑜𝑚𝑝𝑙𝑒𝑥 (𝑚𝑖𝑛𝑖𝑚𝑖𝑧𝑒𝑑) − 𝐸𝑙𝑖𝑔𝑎𝑛𝑑 (𝑚𝑖𝑛𝑖𝑚𝑖𝑧𝑒𝑑) − 𝐸𝑟𝑒𝑐𝑒𝑝𝑡𝑜𝑟 (𝑚𝑖𝑛𝑖𝑚𝑖𝑧𝑒𝑑)

### 300 Statistical Analysis

301 Three replicates were used in each experiment unless otherwise stated. All results were
302 presented as means standard deviations. A one-way ANOVA was used to analyze statistically
303 differences at a *P*-value of 0.05 (95% confidence interval) in conjugation with the Dunnett test using
304 Graph pad prism version 9.4.1 (California. San Diego)

# 305 Results

## 306 Isolation and Structural Characterization

307 The whole plant of *P. minima* Linn. yielded nine compounds **1-9**, including a new
308 aminophysalin (**1**)**.** These compounds were characterized by spectroscopic techniques (Fig 1)
309 Compound **1** was obtained as a colorless crystalline solid. The ESI-MS of 1 exhibited the
310 [M+H]^+^ at *m/z*: 526.2059 [C28H32NO9+H; 526.3072]. The IR spectrum showed a strong
311 absorption at 3463 cm^-1^, indicating the presence of OH groups. The absorption band at 1732
312 cm^-1^ is a characteristic absorption of physalin, which appeared due to a five-membered lactone ring.
313 Furthermore, the IR absorption at 1656 cm^-1^ was assigned to an α,β-unsaturated ketone [36-
314 38]. The^1^H-NMR spectrum (Table 1) of compound **1** showed three characteristic methyl
315 singlets, resonated at δ 1.54, 1.85, and 1.04 and assigned to H-19, H-21, and H-28 methyl
316 respectively. The appearance of three mutually coupled olefin carbon signals appeared at δ 6.00

317	(d, *J*2,3 = 10.0 Hz), 6.98 (dd, *J3*,4= 9.5 Hz, *J*3,4 = 6.0 Hz) and 6.12 (dd, *J*4,3= 5.5 Hz) indicated a

318 2-ene-1-one system in ring A. The presence of nitrogen-containing bridge between C-15, and
319 C-27 (*i.e* entirely different from other physalins) was inferred from the appearance of a pair of

320	characteristic signals δ 4.17 (dd, *J*27,25 = 20.5 Hz, *J*27,27= 10.0 Hz) and δ 4.46 (*J*27,27 = 21) for

321 H2-27 [39]. An overlapped signal also appeared at δ 4.49 indicating the hydroxylation at
322 C-6 carbon [40]. ^13^C-NMR spectra (Table 1) showed 28 carbon signals, assigned to eleven
323 quaternary, nine methine, five methylene, and three methyl carbons. There were four carbon
324 signals with chemical shift above δ 170, and assigned to two ketone carbonyls [δc 210.7 (C-1),
325 δc 208.8 (C-14)] and two ester carbonyls [δc 171.3 (C-18), δc 173.0 (C-26)]. The presence of
326 an additional signal at δ 164.9 (C-15), and a downfield shift of C-27 at δ 47.7 suggesting the
327 nitrogen-containing bridge between C-15 and C-27. The presence of carbonyl carbon at C-14
328 was supported by key HMBC correlations of δ 3.13 (H-8) and 2.52 (H-16) with δc 208.8 (C-
329 14) and 164.9 (C-15), respectively (Fig 2). The HMBC correlation of H2-27 (δ 4.46, 4.17) with
330 164.9 (C-15) supported the nitrogen-containing bridge. COSY correlation between H-25 and
331 H-27 was also supporting the proposed structure. The OH at C-6 was deduced based on
332 HMBC correlations of H-4 (δ 6.12) with C-6 (δ 70.4) (Fig 2).
333 The structure of compound **1** was unambiguously deduced based on single-crystal X-ray
334 diffraction analysis (Figs 4 and 5). The ORTEP diagram depicted that the molecule consists of
335 seven	fused	rings,	*i.e.*	rings	A	(C1-C5/C10),	B	(C5-C10),	C(C8/C9/C11-17),	D

336	(C13/C17/C18/C20/O9/O10), E (C16/C17/C20/C22-C24), F (C22-C26/O-9), and G (C-15/C-

337 16/C-24/C27-N1). Rings A and B transfused with each other through C5/C10 linkage and
338 exists in half chair and chair conformations, respectively. Ring C (C8/C9/C11-17) appeared in
339 twisted-boat-chair conformation with the cleavage of ether linkage between rings D and E as
340 appeared	in	other	reported	physalins.	Tetrahydropyridine	moiety	*i.e.*	ring	G
341 (C15/C16/C24/C27N1) is completely different from reported physalin [36-37]. The result
342 showed that C6-OH and 28-CH3 were on the same side and assigned to be β-oriented. Whereas
343 C13-OH, C17-OH, and C21-CH3 were deduced to be α-oriented (Fig 4). In the crystal lattice
344 molecules found to be linked to form a three-dimensional network (Fig. 5). Previously reported,
345 aminophysalin A [39] was the first physalin having an unusual structural feature in with a
346 nitrogen atom. ,Based on spectroscopic compound **1** was s found to be the second member
347 of aminophysalin series.
348 Compounds **2-5** were identified as previously reported physalin B (**2**) [41, 42], 5β, 6β-
349 epoxyphysalin B (**3**) [42], 5α-ethoxy-6ß-hydroxy-5,6-dihydrophysalin B (**4**) [39, 43], and
350 physalin H (**5**) [44] by comparison of the reported spectroscopic data. These compounds had
351 first reported from *P. angulata*, *P. alkekengi,* and *P. minima*, respectively.
352 Compound **6** was isolated here first time from *Physalis minima.* Its structure was previously
353 reported only based on a tentative assignment based on the fragmentation pattern
354 studied through UHPLC-QTOF-MS/MS analysis *Physalis alkekengi L. var. franchetii* (Mast.)
355 [38]. Therefore, this is the first detailed report of the isolation and validated structure
356 characterization of 5ß,6ß-epoxyphysalin C (**6**). Compound **6** was obtained as a white solid. The
357 ESI-MS [M+H]^+^m/z: 527.1907 [C28H32NO9+H; 527.1912]. The IR spectrum showed strong
358 absorption at 3427, 1782, and 1656 indicating the presence of OH groups, five-membered
359 lactone ring and α,β-unsaturated ketone, respectively. The ^1^H-NMR spectrum (Table 1)
360 showed three characteristic methyl singlets at δ1.21, 1.92, and 1.58, and assigned to H3-19, H3-
361 21, and H3-28 methyl respectively. Two mutually coupled olefin proton signals appeared at δ

362	5.99 (dd, *J*2,4 = 10.0 Hz, *J*2,3 = 2.5 Hz) and δ 6.83 (ddd, *J*2,3 = 3.0 Hz, *J*3,4 = 6.0 Hz, *J*3,4 = 10.0

363 Hz) indicated a 2-ene-1-one system in ring A. A one-proton downfield broad singlet at δ 3.24
364 (d, *J*=3.0 Hz) was assigned to the C-6 methine proton, geminal to oxy group. The assigned C-
365 6 proton at δ 3.24 was further supported by HMBC correlations with C-8 (δ 39.1) and C-7 (δ
366 26.4) (Fig. 2). HMBC correlation of H-3 (δ 6.83) with C-5 (δ 61.8) supported the presence of
367 an epoxide between C-5 and C-6. The low *J* value (*J*= 3.0 Hz) and comparison of the chemical
368 shift with a known physalin (5β,6β-epoxyphysalin B) suggested α-orientation of H-6 [32]. Two
369 characteristic signlets at δ 6.64 (s) and 5.72 (s) indicating the absence of oxygen-containing
370 a bridge between C-14 and C-27. An olefin at C-27 was inferred by HMBC correlations of H2-

371	27 (δ 6.64, δ 5.72) with C-26 (δ 163.7) and C-24 (δ 36.6). The ^13^C-NMR data (Table 1) showed

372 28 carbon signals assigned to twelve quaternary, seven methines, six methylene, and three
373 methyl carbons. The signal at δ 64.4 was assigned to C-5 and indicated the epoxidation at C-6.
374 Two additional quaternary signals at δ 102.1 and 139.3 were assigned to C-14 and C-24,
375 respectively. NOESY correlation between the H-9 (δ 2.51) and H-6 (δ 3.24) indicated a β
376 epoxide (Fig 3). Compound **6** was identified as 5β, 6β-epoxyphysalin C based on
377 spectroscopic analysis.
378 Compounds **7** and **9** were identified as known physalins G (**7**) and physalin D (**9**) through
379 comparison of their reported spectroscopic data. [40, 45]
380 The first report for the isolation of physalin K (**8**) was from *Physalis minima* in 1980 [40].
381 However, the structure was extensively revised later based on spectroscopic techniques in
382 1995 [44]. We have studied the structural parameters of physalin K (**8**) based on single-
383 crystal X-ray diffraction data and found it in good agreement with the revised structure.
384 Like other physalins, the molecular skeleton was found to have a rigid framework, consisting of eight
385 condensed rings. Two six-membered rings *i.e.* ring A (C1/C2/C5/C10/O2/O3) and B (C2-
386 C5/O2/O3) linked together through epi-dioxy linkage at C2 and C5 adopts screw boat
387 conformation. Ring C (C5-C10) is transfused with ring A through C5/C10 bonds and exists in
388 chair conformation. The eight-membered ring D (O7/C8/C9/C11-C13/C17/C14) adopts a
389 boat-chair conformation, connected with two *spiro*-fused rings E (C13/C17/C18/C20/O9/O10)
390 and F (C14-C17/O7) through O7/C13/C14/C17- /O7, while dimethyl substituted six-membered
391 ring G (C-16/C-17/C-22---C-24) is connected with two lactone ring moieties of H (C14-

392	C16/C24-C27/O6) and I (C22-C26/O11/O12) along C16/C23-C25 linkage (Figs 6 and 7).

## 393 Anti-Leishmanial Activity

394 To evaluate the anti-leishmania activity, *Physalis minima* extracts (Ethanol/water, Pet
395 ether, Dichloromethane, and Ethyl acetate), active fractions (A-D), and pure physalins **1**-**9** were
396 incubated in various concentrations with axenic cultures of *L. tropica* and *L. major* in
397 comparison to three commercially available drugs, amphotericin B, pentamidine, and
398 miltefosine (Tables 2 and 3).	The IC50 values (Table 3) indicated that chloro substituted
399 physalin H (**5**) was the most active member (IC**50 =** 9.59 ± 0.27 μM) of the series, followed by

400	physalins B (**2**, IC**50 =**13.33 ± 0.098 μM), physalins G (**7**, IC**50 =**19.49 ± 0.02 μM), and 5ß,6ß-

401 epoxyphysalin C (6, IC**50 =** 23.76 ± 1.10 μM) against *L. tropica* and showed a more potent
402 activity than the standards pentamidine (IC**50 =** 27.20 ± 0.01 μM) and miltefosine (IC**50 =** 42.75
403 ± 1.03 μM). The anti-leishmanial activity of physalins B (**2**), 5ß,6ß-epoxyphysalin B (**3**) and
404 physalin H (**5**) was found in agreement with previous data reported by us [23] against *L. major,*
405 in comparison to amphotericin B. In the current study, the comparison of the anti-leishmanial activity
406 of physalins B (**2**), 5ß,6ß-epoxyphysalin B (**3**), and physalin H (**5**) against *L. major* include
407 comparison with pentamidine and miltefosine. The results indicate that growth inhibition
408 potential of physalins B (**2,** IC**50 =** 3.04 ± 1.12 μM), 5ß,6ß-epoxyphysalin B (**3,** IC**50 =** 3.76 ±
409 0.85 μM) and physalin H (**5,** IC**50 =** 3.34 ± 0.64 μM). These were several-fold more potent than
410 the standard drugs pentamidine (IC**50 =** 27.20 ± 0.015 μM) and miltefosine (IC**50 =** 25.55 ± 1.03
411 μM). Compounds **1**, **4,** and **8** appeared to be inactive against both *L. major* and *L. tropica* strains
412 (Table 3). Compounds **3**, **5**, and **6** showed low selectivity index (SI) values of 0.19, 0.119, and
413 0.007, respectively against *L. major* tested on a 3T3 cell line. However, compound **2** showed SI
414 value of 3.28. All these compounds showed <1 SI index against *L. tropica* cultured on 3T3
415 cells. Moreover, on the human fibroblast cell line (BJ), compounds **2**, **3**, **5**, and **6** showed ≥1
416 SI values of 3.5, 7.34, 3.11, and 1.19 against *L. major,* respectively. While compounds **3**, **5**,
417 and **6** also showed SI values of 1.48, 1.08, and 4.20 against *L. tropica*. The SI value higher than
418 1 indicated that these compounds are theoretically more effective and safe drugs. However,
419 compound 2 demonstrated a SI value of 0.8 on *L. tropica*. (Tables 5-8)
420 420

## 421 Anti-Leishmanial Activity against MIL-resistant *L*. *tropica*

422 Furthermore, compounds **2-9** were also evaluated for their potential against the Miltefosine-
423 unresponsive strain (MIL resistant) *Leishmania tropica*. Compounds **4** (IC50 = 74.65 ± 0.81
424 µM) and **7** (IC50 = 39.44 ± 0.65 µM) were found to be more potent antileishmanial agents than the
425 standard Miltefosine (IC50 = 169.55 ± 0.78 µM). It is interesting to be noted that compound **4**
426 found to be inactive against clinical isolates of *L*. *major* and *L*. *tropica* (Table 4).

## 427 Cytotoxicity of Physalin against BJ (Human Fibroblast) Cell Lines

428 Cytotoxicity *Physalis minima* extracts (Ethanol/water, Pet ether, Dichloromethane, and Ethyl
429 acetate), active fractions (A-D), and pure physalins **2**-**9** were evaluated against normal
430 fibroblast (3T3) and BJ (human fibroblast) cells lines, and all tested compounds were found to
431 be non-cytotoxic in nature (Tables 5 and 6).

## 432 Docking Studies

433 Compounds with significant *in vitro* anti-leishmanial activities proceeded for docking
434 studies against various therapeutically important enzymes involved in different metabolic
435 pathways. Among various targets, compounds (**2**, **3**, **5**, **6**, **7**, and **9**) showed the best binding
436 interaction with phosphoglucose isomerase and *N*-myrositoyltransferase. The docking scores
437 were in the range of -4.3 to -5.7 Kcal/mol (Fig 8), while the binding energies were in the range
438 of -11 to -75 Kcal/mol (Fig 9).

# 439 Discussion:

440 In the last few decades, extensive research on medicinal plants has contributed to increased
441 of natural products against health disorders globally. In continuation of our work to explore the
442 biological properties of medicinal plants, our group has previously reported the *in vitro* anti-
443 leishmanial effect of physalins B (**2**), 5ß,6ß-epoxyphysalin B (**3**) and physalin H (**5**), and other
444 isolated physalins from *Physalis minima* against promastigotes of *L. major* [21, 22], in
445 comparison to amphotericin B. Amphotericin B is the drug of choice for the treatment of
446 various parasitic and fungal infections, including leishmaniasis. However, the clinical use of
447 amphotericin B is limited because of systematic toxicities, and lack of accessibility and
448 affordability [47]	The	recommended	least	toxic,	non-conventional	amphotericin	B
449 formulations, such as Fungizone®, AmBisome^®^, Abelcet^®^, and Amphocil^®^ are expensive, and,
450 therefore, out of the reach of the poor patients [48, 49]. Unfortunately, the first line pentavalent
451 antimonials are also not available in many leishmanial endemic countries. Therefore,
452 alternatives such as pentamidine, and most recently miltefosine are the recommended US-FDA
453 approved drugs, prescribed by dermatologists for cutaneous leishmaniasis. Therefore, in
454 the current study of the inhibition abilities of isolated physalins towards promastigotes of *L. major*
455 and *L. tropica* were studied *in vitro,* and compared with amphotericin B, pentamidine, and
456 miltefosine. The physalins B (**2**), 5ß,6ß-epoxyphysalin B (**3**), and chloro substituted Physalin
457 H (**5**) were previously reported by us as promising candidates with significant abilities to
458 reduce the growth of promastigotes of *L. major,* in comparison to amphoterin B [21]. The
459 published results were further validated in the present study and we further conclude that in
460 addition to amphotericin B, these tested compounds also have several-fold more inhibitions
461 potential against promastigotes of *L. major* than pentamidine (IC**50 =** 27.20 ± 0.015 μM), and
462 miltefosine (IC**50 =** 25.55 ± 1.03 μM). In the current study, the test compound also showed potently
463 anti-leishmanial abilities against *L. tropica* promastigotes *in vitro,* and appeared to be several
464 fold more active than tested standards pentamidine (IC**50 =** 27.20 ± 0.01 μM), and miltefosine
465 (IC**50 =** 42.75 ± 1.03 μM). The activity of some selected compounds was also evaluated against
466 the lab generated a MIL-resistant line. Compound **2** showed SI value of >1 while compounds **3**,
467 **5**, and **6** showed <1 SI against *L. major* on 3T3 cell line. Compounds **3**, **5**, and **6** showed >1 SI
468 values against both *L. major* and *L. tropica* cultured on BJ cell line. However, compound **2**
469 demonstrated >1 SI value only against *L. tropica*.
470 The alkylphosphocholine drug, miltefosine possesses its potential against several parasitic
471 species and different cancer cells, along with activity against various pathogenic fungi and
472 bacteria [50]. Knowledge of experimental MIL resistance in Leishmania is limited to defects
473 in drug internalization (defective inward translocation of MIL) and increased drug efflux [51].
474 A major potential drawback in the use of miltefosine for the treatment of leishmaniasis could be
475 the relatively rapid generation of drug resistance in vitro [52]. According to Temmy Sunyoto
476 et al, 2018 miltefosine is considered a breakthrough in the treatment, making it feasible to
477 eliminate a regional disease, unfortunately, the acquisition of resistance is of major concern.
478 Mechanisms that are responsible for the resistance acquisition in *Leishmaina* parasite against
479 miltefosine includes a reduction in drug uptake, increased efflux, and alteration in permeability
480 of the plasma membrane [53-57]. Due to this type of data and research highlights, there is a
481 the necessity to find out the alternative therapeutic options for leishmaniasis. During the study
482 presented the resistant strain was generated and libraries of compounds were evaluated against
483 the developed line. *L. tropica* MIL-unresponsive / resistant parasites were generated by using
484 the step-wise selection of drug-miltefosine up to the concentration of 196 μM. No marked
485 difference in growth patterns was also analyzed between WT and MIL-resistant strains.
486 Compounds **4** and **7** were observed as significantly potent as compared to the miltefosine,
487 revealing the potential of these natural compounds against drug –unresponsive strains.
488 However, these results of the current study demonstrated the promising anti-leishmanial
489 the potential of physalins against *L. major* and *L. tropica* promastigotes that need to be further
490 validated by *in vivo* studies as an anti-leishmanial agent for oral use.
491 Targeting the metabolic and biochemical pathways of leishmania is one of the most appropriate
492 therapeutic strategy. Following this rationale, compounds **2**, **3**, **5-7**, and **9** with significant *in-*
493 *vitro* antileishmanial activity was validated through docking studies against various metabolic
494 enzymes, to predict their mechanism of action. For this purpose, various enzymes from
495 major	metabolic	pathways	were	selected	including	glycolysis	**(**pyruvate	kinase,
496 glyceraldehyde-3-phosphate dehydrogenase, phosphoglucose isomerase), folate pathway
497 (pteridine	reductase),	gluconeogenesis	(fructose-1,6-bisphosphatase	cytosolic	and
498 mitochondrial fumarate hydratase), pyrimidine biosynthesis (dihydroorotate dehydrogenase),
499 lipid metabolism (*N*-myrositoyl transferase), posttranslational modification (SAM-dependent
500 methyltransferase), and cell signaling (glycogen synthase kinase, MAP kinase). Docking
501 scores were observed in the range of -2 to -7 Kcal, while the binding energies were in the range
502 of -11 to -75 Kcal/mol. The results were then compared with the docking scores and binding
503 energies of antileishmanial clinical drugs including amphotericin B, pentamidine, and
504 miltefosine, and were found to be comparable with them. The results further indicated that the
505 compounds might affect the glycolytic and lipid metabolic pathways by targeting
506 phosphoglucose isomerase and *N*-myristoyl transferase, respectively.

# 507 Conclusion

508 Leishmaniasis, a neglected tropical disease is a rapidly growing infection in more than 98
509 countries of the world. The presented study concluded with the identification of natural steroidal
510 lactones i.e. physalins as potential candidates to be explored further against cutaneous
511 leishmaniasis. The complex structures of all natural compounds **1-9** were elucidated *via*
512 combined use of MS, IR, and NMR spectroscopic techniques. The assigned structures of
513 compounds **1**, and **8** were further supported by single-crystal X-ray diffraction studies. Anti-
514 leishmanial activities of isolated physlains were evaluated against *L. major* and *L. tropica .* The
515 literature survey revealed that all of the physalins possess anti-leishmanial activity against *L.*
516 *tropica* (clinical isolate of Pakistan), reported for the first time. The promising results
517 of compounds 4 and 7 against the miltefosine-unresponsive *Leishmania tropica* strain (MIL
518 resistant) concluded the anti-leishmanial potential of tested compounds against resistant strain.
519 The compounds were also able to interact with therapeutically relevant enzymes of leishmania
520 including phosphoglucose isomerase, and *N*-myrositoyltransferase.

# 521 References

522 1. Mann S, Frasca K, Scherrer S, Henao-Martínez AF, Newman S, Ramanan P, Suarez
523 JA. A Review of Leishmaniasis: Current Knowledge and Future Directions. Curr Trop

524	Med Rep. 2021 Mar;17:1-12.

525 2. CDC.	Epidemiology	and	risk	factors.	[Available	from:	https://
526 [www.cdc.gov/parasites/leishmaniasis/epi.html](http://www.cdc.gov/parasites/leishmaniasis/epi.html)].
527 3. WHO.	Leishmaniasis	[Available	from:	https:/[/www.who.int/newsroom/fa](http://www.who.int/newsroom/fact-)c[t-](http://www.who.int/newsroom/fact-)
528 sheets/detail/leishmaniasis].
529 4. Ruiz-Postigo JA, Jain S, Maia-Elkhoury AMAN, Valadas S, Warusavithana S, Osman
530 M, Lin Z, Beshah A, Yajima A, Gasimov E. Global leishmaniasis surveillance: 2019–
531 2020, a baseline for the 2030 roadmap. Weekly Epidemiological Record, 2021;96(35):

532	401+.

533 5. Haldar AK, Sen P, Roy S. Use of antimony in the treatment of leishmaniasis: current
534 status and future directions. Mol Biol Int. 2011;2011:571242.
535 6. Zahedifard F, Rafati S. Prospects for antimicrobial peptide-based immunotherapy
536 approaches in *Leishmania* control. Expert Rev Anti Infect Ther. 2018;16(6):461-469.
537 7. Sundar S, Chakravarty J. Antimony toxicity. Int J Environ Res Public Health. 2010

538	Dec;7(12):4267-77

539	8. Choudhary M, Kumar V, Malhotra H, Singh S. Medicinal plants with potential anti-

540	arthritic activity. J Intercult Ethnopharmacol. 2015;4(2):147-79.

541	9. Petrovska BB. Historical review of medicinal plants' usage. Pharmacogn Rev.

542	2012;6(11):1-5.

543	10. Šantić Ž, Pravdić N, Bevanda M, Galić K. The historical use of medicinal plants in

544	traditional and scientific medicine. Psychiatr Danub. 2017;29:787-792.

545	11. Vs A, Kp M, Krishna JG. Preliminary pharmacognostical and phytochemical

546	evaluation of *Physalis minima* Linn. (Ṭankārī). *J Pharmacogn Phytochem. 2018;7:*

547	1664-1669.

548	12. Patel T, Shah K, Jiwan K, Shrivastava N. Study on the antibacterial potential of physalis

549	minima linn. Indian J Pharm Sci. 2011;73(1):111-5.

550	13. Khan MA, Khan H, Khan S, Mahmood T, Khan PM, Jabar A. Anti-inflammatory,

551	analgesic and antipyretic activities of *Physalis minima* Linn. J Enzyme Inhib Med

552	Chem. 2009;24(3):632-7.

553	14. K. S., A. S. Screening of anti-diabetic activity of *Physalis Minima* Fruits. Int. J. Pharma

554	Bio Sci. (2020):11.

555 15. Chothani DL, Vaghasiya HUA. phyto-pharmacological overview on *Physalis minima*
556 Linn. Indian J Nat Prod Resour. 2012:3(4), 477-482.
557 16. Sun JL, Jiang YJ, Cheng L. Two new physalin derivatives from *Physalis alkekengi* L.
558 var. franchetii (Mast.) Makino. Nat Prod Res. 2021;35(2):203-206.
559 17. Zhang M, Jiang B, He X, Cao S, Ding L, Kang N, Chen L, Qiu F. New cytotoxic
560 withanolides from *Physalis minima*. Fitoterapia. 2020;146:104728.
561 18. Wu J, Zhang T, Yu M, Jia H, Zhang H, Xu Q, Gu Y, Zou Z. Anti-inflammatory
562 Withanolides from Physalis minima. ACS Omega. 2020;5(21):12148-12153.
563 19. Le Canh VC, Le Ba V, Thi Hai Yen P, Le Thi L, Thi Thuy Hoai P, Huu Dat TT, Thao
564 DT, Bach LG, Kim YH, Tuan Anh HL. Identification of potential cytotoxic inhibitors
565 from *Physalis minima*. Nat Prod Res. 2021; 35(12):2082-2085.
566 20. Choudhary	MI,	Yousuf	S,	Samreen,	Ahmed	S	Atta-ur-Rahman. New
567 leishmanicidalphysalins from *Physalis minima* , Nat Prod Res. 2007; 21:877-883.
568 21. Choudhary MI, Yousaf S, Ahmed S; Samreen, Yasmeen K; Atta-ur-Rahman.
569 Antileishmanial physalins from *Physalis minima*. Chem Biodivers. 2005;2(9):1164-73.
570 22. Choudhary MI, Yousaf S, Ahmed S; Samreen, Yasmeen K, Atta-ur-Rahman.
571 Discovery of leishmanicidal agents from medicinal plants. Pure Appl Chem. 2008;

572	80(8):1783-1790.

573	23. Silver J, Let Us Teach Proper Thin Layer Chromatography Technique! J Chem Educ. 2020;97

574	(12): 4217-4219.

575 24. Sasidharan S, Chen Y, Saravanan D, Sundram KM, Yoga Latha L. Extraction, isolation
576 and characterization of bioactive compounds from plants' extracts. Afr J Tradit
577 Complement Altern Med. 2011;8 :1-10.
578 25. .Choudhary MI, Rizvi F, Siddiqui H, Yousuf S, Zafar H, Shaikh M. Microwave assisted
579 Biology-Oriented Drug Synthesis (BIODS) of new N,N′-disubstituted benzylamine
580 analogous of 4-aminoantipyrine against leishmaniasis – In vitro assay and in silico-
581 predicted molecular interactions with key metabolic targets. Bioorganic Chemistry,

582	2022;120: 105621

583 26. Hendrickx S, Inocencio da Luz RA, Bhandari V, Kuypers K, Shaw CD, Lonchamp J,
584 et al. Experimental induction of paromomycin resistance in antimony-resistant strains
585 of L. donovani: outcome dependent on in vitro selection protocol. PLoS neglected
586 tropical diseases. 2012;6: e1664.
587 27. Turner KG, Vacchina P, Robles-Murguia M, Wadsworth M, McDowell MA, Morales
588 MA Fitness and Phenotypic Characterization of Miltefosine-Resistant Leishmania
589 major. PLoS Negl Trop Dis 2015;9: e0003948.
590 28. Dimas, K, Demetzos, C, Marsellos, M, Sotiriadou, R, Malamas, M, Kokkinopoulos, D.
591 Cytotoxic activity of labdane type diterpenes against human leukemic cell lines in vitro.

592	Planta. Med. 1998, 64, 208-211. DOI: 10.1055/s-2006-957410

593 29. Schrödinger Release 2020-1: Protein Preparation Wizard; Epik, Schrödinger, LLC,
594 New York, NY, 2020; Impact, Schrödinger, LLC, New York, NY, 2020; Prime,
595 Schrödinger, LLC, New York, NY, 2020.
596 30. Sastry GM, Adzhigirey M. Day T. Annabhimoju R. Sherman W. Protein and ligand
597 preparation: parameters, protocols, and influence on virtual screening enrichments.

598	*J Comput Aided Mol Des*. 2013; 27(3):221-234.

599 31. Schrödinger Release 2020-1: LigPrep, Schrödinger, LLC, New York, NY, 2020.
600 32. Schrödinger Release 2020-1: Glide, Schrödinger, LLC, New York, NY, 2020.
601 33. Halgren TA, Murphy RB, Friesner RA, Beard HS, Frye LL, Pollard WT, Banks JL.
602 Glide: a new approach for rapid, accurate docking and scoring. 2. Enrichment factors
603 in database screening. J Med Chem. 2004; 47(7):1750-1759.
604 34. Friesner RA, Banks JL, Murphy RB, Halgren TA, Klicic JJ, Mainz DT, Shaw DE.
605 Glide: a new approach for rapid, accurate docking and scoring. 1. Method and
606 assessment of docking accuracy. J Med Chem. (2004);47(7):1739-1749.
607 35. Schrödinger Release 2020-1: Prime, Schrödinger, LLC, New York, NY, 2020
608 36. YANG Yan-Jun, SHA Cong-Wei, CHEN Mei-Guo, HU Ling. Extraction and crystal
609 structure of physalin B. *Chinese J Struc Chem*. 2014;33(5): 795-800.
610 37. Mangwala Kimpende P, Lusakibanza M, Mesia K, Tona L, Tits M, Angenot L,
611 Frédérich M, Van Meervelt L. Isolation, pharmacological activity and structure
612 determination of physalin B and 5β,6β-epoxyphysalin B isolated from Congolese
613 *Physalis angulata* L. *Acta Crystallogr C*. 2013 Dec 15; 69:1557-62.
614 38. Huang C, Xu Q, Chen C, Song C, Xu Y, Xiang Y, Feng Y, Ouyang H, Zhang Y, Jiang
615 H. The rapid discovery and identification of physalins in the calyx of Physalis alkekengi
616 L.var.franchetii (Mast.) Makino using ultra-high performance liquid chromatography-
617 quadrupole time of flight tandem mass spectrometry together with a novel three-step
618 data mining strategy. J Chromatogr A. 2014 Sep 26;1361:139-52.
619 39. Men RZ, Li N, Ding WJ, Hu ZJ, Ma ZJ, Cheng L. Unprecedent aminophysalin from
620 *Physalis angulata*. Steroids. 2014;88:60-5.
621 40. Row LR, Reddy KS, Sarma N, Matsuura T, & Nakashima R. New physalins from
622 *Physalis angulata* and *Physalis lancifolia*. Structure and reactions of physalins D, I, G

623	and K. Phytochemistry, 1980; 19:1175-1181.

624	41. Kawai M, Makino B, Taga T, Miwa Y, Yamamoto T, Furuta T, Yamamura H, Butsugan

625	Y, Ogawa K, Hayashi M. Crystal structures of 5α,6α-epoxy and 2,3-dihydro derivatives

626	of Physalin B, a 13,14-Seco-16,24-cyclosteroid, and Their 1H NMR Spectral Analysis.

627	Bull Chem Soc Jpn. 1994:67, 222-226.

628 42. Glotter E, Kirson I, Abraham A, Sethi PD, Subramanian SS. Steroidal constituents of
629 *Physalis minima* (solanaceae). J Chem Soc Perkin 1. 1975;(14):1370-4.
630 43. Kawai M, Makino B, Yamamura H, Butsugan Y. (1996). Upon ‘physalin L’ isolated
631 from Physalis minima. Phytochemistry, 43, 661-663.
632 44. Bunsho Makino, Masao Kawai, Toichi Ogura, Masaki Nakanishi, Hatsuo Yamamura,
633 and Yasuo Butsugan, Structural Revision of Physalin H Isolated from *Physalis*
634 *angulate*. Journal of Natural Products 1995;58(11):1668-1674,
635 45. Januário AH, Filho ER, Pietro RC, Kashima S, Sato DN, França SC. Antimycobacterial
636 physalins from *Physalis angulata* L. (Solanaceae). Phytother Res. 2002:16(5):445-8.
637 46. Makino B, Kawai M, Iwata Y, Yamamura H, Butsugan Y, Ogawa K, Hayashi M.
638 Physalins Possessing an Endoperoxy Structure from *Physalis alkekengi* var. *francheti*.
639 Structural Revision of Physalin K. Bull Chem Soc Jpn. 1995; 68:219-226.
640 47. de Menezes JP, Guedes CE, Petersen AL, Fraga DB, Veras PS. Advances in
641 development of new treatment for leishmaniasis. *Biomed Res Int.* 2015;2015:815023.
642 48. Clemons KV, Stevens DA. Comparative efficacies of four amphotericin B
643 formulations--Fungizone, amphotec (Amphocil), AmBisome, and Abelcet--against
644 systemic murine aspergillosis*. Antimicrob Agents Chemother.* 2004;48(3):1047-50.
645 49. Cuddihy G, Wasan EK, Di Y, Wasan KM. The development of oral amphotericin B to
646 treat systemic fungal and parasitic infections: has the myth been finally realized?

647	*Pharmaceutics*. 2019;11(3):99.

648 50. Dorlo TP, Balasegaram M, Beijnen JH, de Vries PJ. Miltefosine: a review of its
649 pharmacology and therapeutic efficacy in the treatment of leishmaniasis. The Journal
650 of antimicrobial chemotherapy. 2012;67: 2576–97.
651 51. Perez-Victoria FJ, Sanchez-Canete MP, Seifert K, Croft SL, Sundar S, Castanys S, et
652 al. Mechanisms of experimental resistance of Leishmania to miltefosine: Implications
653 for clinical use. Drug Resist Updat. 2006;9 :26–39.
654 52. Varela-M RE, Villa-Pulgarin JA, Yepes E, Müller I, Modolell M, Muñoz DL, et al. In
655 Vitro and In Vivo Efficacy of Ether Lipid Edelfosine against Leishmania spp. and SbV-
656 Resistant Parasites. PLoS Negl Trop Dis 2012;6: e1612.
657 53. Perez-Victoria FJ, Castanys S, Gamarro F. Leishmania donovani resistance to
658 miltefosine involves a defective inward translocation of the drug. Antimicrob Agents

659	Chemother 2003;47: 2397–2403.

660 54. Seifert K, Matu S, Pe´rez-Victoria FJ, Castanys S, Gamarro F, Croft SL.
661 Characterisation	of	Leishmania	donovani	promastigotes	resistant	to
662 hexadecylphosphocholine (miltefosine). Int J Antimicrob Agents 2003;22: 380–387.
663 55. Kulshrestha A, Sharma V, Singh R, Salotra P. Comparative transcript expression
664 analysis of miltefosine-sensitive and miltefosine-resistant Leishmania donovani.

665	Parasitol Res 2014;113:1171–1184.

666	56. Sanchez-Cañete MP, Carvalho L, Victoria-Pe´rez FJ, Gamarro F, Castanys S. Low

667	plasma membrane expression of the miltefosine transport complex renders Leishmania

668	braziliensis refractory to the drug. Antimicrob Agents Chemother 2009;53: 305–313.

| 669 | 31. |
| --- | --- |
| 670 | 57. Mondelaers A, Sanchez-Cañete MP, Hendrickx S, Eberhardt E, Garcia-Hernandez R, |
| 671 | Lachaud L, et al. Genomic and molecular characterization of miltefosine resistance in |
| 672 | Leishmania infantum strains with either natural or acquired resistance through |
| 673  674 | experimental selection of intracellular amastigotes. PLoS One 2016; 11: e0154101. |
| 675  676 | **Table 1:** ^1^H-NMR ^13^C-NMR chemical shift data of compounds **1** and **6** (δ in ppm, *J* in |
| 677 | Hz) |

678

| **C. No.** | **_1_a** | **_1_b** | **_6_a** | **_6_b** |
| --- | --- | --- | --- | --- |
| **1** | - | 208.8 | - | 204.4 |
| **2** | 6.00 (d, *J* 2,3 = 10.0) | 119.8 | 5.99 (dd, *J*2,3 = 10.0, *J*2,4 = 2.5) | 145.5 |
| **3** | 6.98 (dd, *J* 3,4 = 9.5, *J* 3,4 = 6.0) | 140.9 | 6.83 (ddd, *J*3,2 = 10.0, *J*3,4β = 2.5, *J*3,4α = 6.0) | 128.6 |
|  | 6.12 (d, *J* 3,4 = 5.5) | 125.5 | 2.93, m; 2.04, m | 32.9 |
| **5** | - | 155.6 | - | 61.8 |
| **6** | 4.49, m | 70.4 | 3.24 (d, *J*3,4 = 2.5) | 64.6 |
| **7** | 2.10, m; 2.53, m | 29.4 | 2.35, m; 1.71, m | 29.8 |
| **8** | 3.13, m | 39.4 | 2.22, m | 39.1 |
| **9** | 2.57, m | 34.3 | 2.51, m | 35.5 |

| **10** | - | 52.0 | - | 49.8 |
| --- | --- | --- | --- | --- |
| **11** | 2.35, m; 1.65, m | 28.2 | 2.26, m; 1.14, m | 22.4 |
| **12** | 2.54, m; 2.04, m | 29.5 | 2.64, m; 1.76, m | 26.4 |
| **13** | - | 79.4 | - | 79.7 |
| **14** | - | 210.7 | - | 102.1 |
| **15** | - | 171.5 | - | 210.8 |
| **16** | 2.52, m | 56.5 | 2.50, m | 54.6 |
| **17** | - | 83.3 | - | 81.8 |
| **18** | - | 173.0 | - | 171.8 |
| **19** | 1.54, s | 23.0 | 1.21, s | 14.3 |
| **20** | - | 80.0 | - | 82.7 |
| **21** | 1.85, s | 21.7 | 1.92, s | 20.9 |
| **22** | 4.50, m | 77.3 | 4.58, (dd, *J*22,23 = 4.5, *J*22,23 = 2) | 76.8 |
| **23** | 2.05, m; 1.97, m | 31.6 | 1.99, m; 2.11, m | 31.7 |
| **24** | - | 29.1 | - | 36.6 |
| **25** | 2.36, m | 42.2 | - | 139.3 |
| **26** | - | 164.9 | - | 163.7 |
| **27** | 4.17 (dd, *J*27,27 = 10.0, *J*27,25 = 20.5)  4.46 (*J*27,27 = 21) | 47.7 | 4.20 (6.64, s; 5.72, s) | 133.3 |
| **28** | 1.04, s | 28.2 | 1.58, s | 27.3 |

683

| 679 | **^a^** 500 MHz, CDCl3 |
| --- | --- |
| 680 |  |
| 681 | **Table 2: Anti-Leishmanial Activity of Extracts and Fraction against** |
| 682 | ***L. major* and *L. tropica.*** |

|  |  | *L. major* | | | | *L. tropica* | | | |
| --- | --- | --- | --- | --- | --- | --- | --- | --- | --- |
|  |  | **IC50**  **(*μ*g/ml**  **± SD)** | **p-value** Amphote ricin | **p-value** Pentami dine | **p-value** Miltefo sine | **IC50**  **(*μ*g/ml**  **± SD)** | **p-value** Amphote ricin | **p-value** Pentami dine | **p-value** Miltefo sine |
| Extra cts | Ethanol/water | >100 | - |  |  | >100 | - |  |  |
|  | Pet ether | >100 | - |  |  | >100 | - |  |  |
|  | Dichlorom ethane | 19.01±  0.7 | <0.0001 | <0.0001 | <0.000  1 | 19.53±  1.3 | <0.0001 | <0.0001 | 0.0021 |
|  | Ethyl acetate | >100 | - |  |  | >100 | - |  |  |

|  | A | 6.67±0.  1 | <0.0001 | <0.0001 | <0.000  1 | 10.16±  2.0 | <0.0001 | 0.0568 | <0.000  1 |
| --- | --- | --- | --- | --- | --- | --- | --- | --- | --- |
| Fracti | B | 9.95±1.  9 | <0.0001 | 0.8898 | 0.5084 | 6.41±0.  074 | <0.0001 | 0.0001 | <0.000  1 |
| ons |  |  |  |  |  |  |  |  |  |
|  | C | 6.30±0.  018 | <0.0001 | <0.0001 | <0.000  1 | 6.34±0.  045 | <0.0001 | <0.0001 | <0.000  1 |
|  | D | 6.37±0.  015 | <0.0001 | <0.0001 | <0.000  1 | 6.37±0.  015 | <0.0001 | 0.0001 | <0.000  1 |
|  | Amphoteri | 3.14 ± |  |  |  | 3.16 ± |  |  |  |
|  | cin | 0.04 |  |  |  | 0.04 |  |  |  |
| Stand ard drugs |  |  |  |  |  |  |  |  |  |
|  | Pentamidin e | 9.25 ± 0.005 |  |  |  | 9.25 ± 0.005 |  |  |  |
|  | Miltefosine | 10.4 ± 0.42 |  |  |  | 17.4 ± 0.42 |  |  |  |

684

685

686

687

### 688 Table 3: Anti-Leishmanial Activity of Compounds 1-9 against

689	***L. major* and *L. tropica.***

690

691	**Table**

692

### 693 Table 3: Anti-Leishmanial Activity of Compounds 1-9 against

694	***L. major* and *L. tropica.***

695

| Compounds | *L. major* | | | | *L. tropica* | | | |
| --- | --- | --- | --- | --- | --- | --- | --- | --- |
|  | **IC50 (*μ*M± SD)** | **p-value**  Amphotericin | **p-value**  Pentamidine | **p-value**  Miltefosine | **IC50 (*μ*M± SD)** | **p-value**  Amphotericin | **p-value**  Pentamidine | **p-value**  Miltefosine |
| 1 | Inactive | - | - | - | Inactive | - | - | - |
| 2 | 3.04 ± 1.12 | 0.9001 | <0.0001 | <0.0001 | 13.33 ± 0.098 | <0.0001 | <0.0001 | <0.0001 |
| 3 | 3.76 ± 0.85 | 0.9997 | <0.0001 | <0.0001 | 18.53 ± 0.28 | <0.0001 | <0.0001 | <0.0001 |
| 4 | Inactive | 0.9999 | <0.0001 | <0.0001 | Inactive | <0.0001 | <0.0001 | <0.0001 |
| 5 | 3.34 ± 0.64 | <0.0001 | <0.0001 | <0.0001 | 9.59 ± 0.27 | <0.0001 | 0.0009 | <0.0001 |
| 6 | 84.79 ± 1.56 | <0.0001 | <0.0001 | <0.0001 | 23.76 ± 1.10 | <0.0001 | <0.0001 | <0.0001 |
| 7 | 52.28 ± 1.18 | 0.9001 | <0.0001 | <0.0001 | 19.49 ± 0.02 | <0.0001 | <0.0001 | <0.0001 |
| 8 | Inactive | - | - | - | Inactive | - | - | - |
| 9 | Inactive | - | - | - | 55.24± 0.01 | <0.0001 | <0.0001 | <0.0001 |
| **Amphotericin** | 3.39 ± 0.043 |  |  |  | 3.42 ± 0.04 |  |  |  |
| **Pentamidine** | 27.20 ± 0.015 |  |  |  | 27.20 ± 0.01 |  |  |  |
| **Miltefosine** | 25.55 ± 1.03 |  |  |  | 42.75 ± ± 1.03 |  |  |  |

696

31

### 697 4: Anti-Leishmanial Activity of Compounds 2-9 against the MIL-

698	**Resistant *L. tropica*.**

|  |  | **MIL Resistant**  ***L. tropica*** |
| --- | --- | --- |
|  | **Compounds** | **IC50**  **(*μ*M± SD)** |
| **Pure Compounds** | **2** | inactive |
|  | **3** | inactive |
|  | **4** | 74.65 ± 0.81 |
|  | **5** | inactive |
|  | **6** | inactive |
|  | **7** | 39.44 ± 0.65 |
|  | **8** | Inactive |
|  | **9** | inactive |
| **Standard drugs** | Miltefosine | 169.55 ± 0.78 |

699

700

701

702

703

704

705

706

707

708

709

710

711

712

713

32

### 714 Table 5: Selectivity Index of Pure Compounds (BJ Fibroblast Cell line)

|  | **Samples** | **BJ cell line CC50 (*μ*M± SD)** | ***L. major***  **IC50 (*μ*M± SD)** | ***L. tropica***  **IC50 (*μ*M± SD)** | ***L. major***  **SI (CC50/ IC50)** | ***L. tropica***  **SI (CC50/ IC50)** |
| --- | --- | --- | --- | --- | --- | --- |
| **Extracts** | Ethanol/water | >100 | >100 | >100 | - | - |
|  | Pet ether | >100 | >100 | >100 | - | - |
|  | Dichloromethane | 26.5±1.1 | 19.01±0.7 | 19.53±1.3 | 1.39 | 1.35 |
|  | Ethyl acetate | >100 | >100 | >100 | - | - |
| **Fractions** | Fraction-A | 10.4 ± 1.0 | 6.67±0.1 | 10.16±2.0 | 1.49 | 1.02 |
|  | Fraction-B | >100 | 9.95±1.9 | 6.41±0.074 | - | - |
|  | Fraction-C | >100 | 6.30±0.018 | 6.34±0.045 | - | - |
|  | Fraction-D | >100 | 6.37±0.015 | 6.37±0.015 | - | - |

715

### 716 Table 6: Selectivity Index of Crude Fractions (BJ Fibroblast Cell line)

717

718

719

| **Samples** | **BJ cell line CC50 (*μ*M± SD)** | ***L. major***  **IC50 (*μ*M± SD)** | ***L. tropica***  **IC50 (*μ*M± SD)** | ***L. major***  **SI (CC50/ IC50)** | ***L. tropica***  **SI (CC50/ IC50)** |
| --- | --- | --- | --- | --- | --- |
| **1** | Non-cytotoxic | Inactive | Inactive | - |  |
| **2** | 10.8 ± 1.5 | 3.04 ± 1.12 | 13.33 ± 0.098 | 3.5 | 0.8 |
| **3** | 27.6 | 3.76 ± 0.85 | 18.53 ± 0.28 | 7.34 | 1.48 |
| **4** | 14.2 ± 0.8 | Inactive | Inactive | - | - |
| **5** | 10.4 ± 1.0 | 3.34 ± 0.64 | 9.59 ± 0.27 | 3.11 | 1.08 |
| **6** | >100 | 84.79 ± 1.56 | 23.76 ± 1.10 | 1.19 | 4.20 |
| **7** | >100 | 52.28 ± 1.18 | 19.49 ± 0.02 | - | - |
| **8** | >100 | Inactive | Inactive | - | - |
| **9** | >100 | Inactive | 55.24± 0.01 | - | - |
| **Amphotericin** | 16.059 | 3.39 ± 0.043 | 3.42 ± 0.04 | 4.37 | 4.69 |

720

721

722

723

724

725

726

### 727 Table 7: Selectivity Index of Pure Compounds (3T3 Mouse Fibroblast Cell line)

| **Samples** | **3T3 Cell line CC50 (*μ*M± SD)** | ***L. major***  **IC50 (*μ*M± SD)** | ***L. tropica***  **IC50 (*μ*M± SD)** | ***L. major***  **SI (CC50/ IC50)** | ***L. tropica***  **SI (CC50/ IC50)** |
| --- | --- | --- | --- | --- | --- |
| **1** | - | Inactive | Inactive | - | - |
| **2** | 10 ± 0.4 | 3.04 ± 1.12 | 13.33 ± 0.098 | 3.28 | 0.007 |
| **3** | 0.72 ± 0.5 | 3.76 ± 0.85 | 18.53 ± 0.28 | 0.19 | 0.038 |
| **4** | 19.6± 0.8 | Inactive | Inactive | - | - |
| **5** | 0.4 ± 0.07 | 3.34 ± 0.64 | 9.59 ± 0.27 | 0.119 | 0.041 |
| **6** | 0.6 ± 0.04 | 84.79 ± 1.56 | 23.76 ± 1.10 | 0.007 | 0.025 |
| **7** | Non-cytotoxic | 52.28 ± 1.18 | 19.49 ± 0.02 | - | - |
| **8** | Non-cytotoxic | Inactive | Inactive | - | - |
| **9** | Non-cytotoxic | Inactive | 55.24± 0.01 | - | - |

728

729

730

731

732

### 733 Table 8: Selectivity Index of Pure Compounds (3T3 Mouse Fibroblast Cell line)

|  | **Samples** | **3T3 cell line CC50 (*μ*M± SD)** | ***L. major***  **IC50 (*μ*M± SD)** | ***L. tropica***  **IC50 (*μ*M± SD)** | ***L. major***  **SI (CC50/ IC50)** | ***L. tropica***  **SI (CC50/ IC50)** |
| --- | --- | --- | --- | --- | --- | --- |
| **Extracts** | Ethanol/water | >100 | >100 | >100 | - | - |
|  | Pet ether | >100 | >100 | >100 | - | - |
|  | Dichloromethane | 4.5 ± 0.5 | 19.01±0.7 | 19.53±1.3 | 0.23 | 0.23 |
|  | Ethyl acetate | >100 | >100 | >100 | - | - |
| **Fractions** | Fraction-A | 12.2 ± 1.5 | 6.67±0.1 | 10.16±2.0 | 7.30 | 1.20 |
|  | Fraction-B | 4.8 ± 1.6 | 9.95±1.9 | 6.41±0.074 | 0.48 | 0.74 |
|  | Fraction-C | 2.7 ± 0.3 | 6.30±0.018 | 6.34±0.045 | 0.42 | 0.42 |
|  | Fraction-D | 3.3 ± 0.3 | 6.37±0.015 | 6.37±0.015 | 0.51 | 0.51 |

734

735

### 736 Cytotoxicity of Extracts and Fraction against 3T3 (Mouse Fibroblast)

737	**and BJ (Human Fibroblast) Cell Lines.**

738

|  |  |  | --- |
| --- | --- | --- | --- |

739

740

37

<https://www.editorialmanager.com/pone/download.aspx?id=31654358&guid=5f1f42a5-7d6f-469b-bf7e-951aeaf4299e&scheme=1>Figure	[Click here to access/download;Figure;Figures_revised.docx](https://www.editorialmanager.com/pone/download.aspx?id=31654358&guid=5f1f42a5-7d6f-469b-bf7e-951aeaf4299e&scheme=1)

**Fig. 1**: The structures of compounds **1–9** isolated from *Physalis minima.*

**Fig. 2**. Key COSY (	) and HMBC (	) correlations in compounds **1** and **6**.

**Fig. 3**. Key NOESY Correlations in Compounds **6**.

**Fig. 4**: ORTEP view of compound **1** at 50% probability level.

**Fig. 5** Crystal packing diagram of compound **1**.

**Fig. 6:** *ORTEP* view of compound **8** at 50% probability level.

**Fig. 7:** Crystal packing diagram of compound **8.**

**Fig. 8.** Docking scores of selected compounds (**2**, **3**, **5**, **6**, **7**, and **9**) against leishmanial phosphoglucose isomerase (carbohydrate metabolism).

**Fig. 9.** Docking scores of selected compounds **2**, **3**, **5**, **6**, **7**, and **9** against leishmanial *N*- myristoyl transferase (lipid metabolism).

Supporting Information

#### [Click here to access/download](https://www.editorialmanager.com/pone/download.aspx?id=31654337&guid=86cebdd5-6773-4853-9a7f-41e3b297043b&scheme=1)

[**Supporting Information** checkcif-comp. 1- Aminophysalin B.pdf](https://www.editorialmanager.com/pone/download.aspx?id=31654337&guid=86cebdd5-6773-4853-9a7f-41e3b297043b&scheme=1)

Supporting Information

#### [Click here to access/download](https://www.editorialmanager.com/pone/download.aspx?id=31654338&guid=9d6a0094-8032-4c0f-a4ae-651b2e428bd3&scheme=1)

[**Supporting Information** checkcif-comp. 7- Physalin K.pdf](https://www.editorialmanager.com/pone/download.aspx?id=31654338&guid=9d6a0094-8032-4c0f-a4ae-651b2e428bd3&scheme=1)

Supporting Information

#### [Click here to access/download](https://www.editorialmanager.com/pone/download.aspx?id=31654339&guid=2f4ea485-619b-4f2f-9a5e-c72a13b7dde6&scheme=1)

[**Supporting Information** pmd_3m_55_ir_0m.cif](https://www.editorialmanager.com/pone/download.aspx?id=31654339&guid=2f4ea485-619b-4f2f-9a5e-c72a13b7dde6&scheme=1)

Supporting Information

[Click here to access/download](https://www.editorialmanager.com/pone/download.aspx?id=31654340&guid=11436d2c-e1e5-445a-8ab2-e8f9e8237ad1&scheme=1)

# [Supporting Information a.cif](https://www.editorialmanager.com/pone/download.aspx?id=31654340&guid=11436d2c-e1e5-445a-8ab2-e8f9e8237ad1&scheme=1)

Supporting Information

#### [Click here to access/download](https://www.editorialmanager.com/pone/download.aspx?id=31629667&guid=b698283b-3e3b-4ce8-9533-998f0fb0c03e&scheme=1)

[**Supporting Information** Supplementary file for Mass and NMR.pdf](https://www.editorialmanager.com/pone/download.aspx?id=31629667&guid=b698283b-3e3b-4ce8-9533-998f0fb0c03e&scheme=1)

Revised Manuscript with Track Changes

###### 1 Anti-leishmanial p~~P~~hysalins - ~~Phytochemcial~~ phytochemcial

2	**i~~I~~nvestigation, *~~I~~in vitro* e~~E~~valuation against clinical and MIL**

3	**r~~R~~esistant *L. tropica* strains and *~~I~~in silico* ~~Studies~~ studies**

4 **Saira Bano**^1^**, Memoona Bibi**^1^**, Saba Farooq^2^, Humaira Zafar**^2^**, Muniza Shaikh**^2^**, Behram**
5 **Khan Khoso**^3^**, SammerYousuf***^1^**, M. Iqbal Choudhary^*^**^1,^ 2, 4
6 ^1^H.E.J. Research Institute of Chemistry, International Center for Chemical and Biological
7 Sciences, University of Karachi, Karachi-75270, Pakistan
8 ^2^ Dr. Panjwani Center for Molecular Medicine and Drug Research, International Center for
9 Chemical and Biological Sciences, University of Karachi, Karachi-75270, Pakistan
10 ^34^Jinnah Post-Graduate Medical Center, Sarwar Shaheed Rd, Karachi Cantonment, Karachi-
11 75510, Pakistan
12 ^43^Department of Biochemistry, Faculty of Science, King Abdulaziz University, Jeddah-21412,
13 Saudi Arabia

14

15

16

17	18

**Formatted:** Tab stops: 5.27", Left

19

20

21

22

23

24 ***Corresponding author:** ~~Prof. Dr. Sammer Yousuf,~~ [dr.sammer.yousuf@gmail.com](mailto:dr.sammer.yousuf@gmail.com) (SY)
25 ***Additional corresponding author:** ~~Prof. Dr. M. Iqbal Choudhary,~~ [iqbal.choudhary@iccs.edu](mailto:iqbal.choudhary@iccs.edu) (MIC)

**Formatted:** Font: No underline

**Formatted:** No underline

26	**Abstract**

27 Cutaneous leishmaniasis (CL)~~, caused by the parasitic protozoa~~ *~~L. major~~* ~~and~~ *~~L. tropica~~*~~,~~ is a
28 major health problem in over 98~~97~~ countries of the world, including Pakistan. The current
29 treatments ~~of leishmaniasis in Pakistan are based on imported repurposed drugs, which are not~~
30 ~~easily accessible. Furthermore, many of them~~ are associated with a number of adverse effects
31 and availability problem of drugs. ~~The development of drug resistance makes the situation~~
32 ~~more difficult to be handle.~~ Therefore, there is an urgent need of easily available and cost
33 effective treatments of cutaneous leishmaniasis (CL) in Pakistan. ~~In continuation of our work~~
34 ~~on medicinal plants,~~ Tthe bioassay-guided fractionation and purification of crude extract of ~~the~~
35 ~~freshly collected~~ *Physalis minima* has led to the isolation of a new aminophysalin B (**1**), ~~along~~
36 ~~with~~and eight known physalins, physalin B (**2**), 5ß,6ß-epoxyphysalin B (**3**), 5α-ethoxy-6ß-
37 hydroxy-5,6-dihydrophysalin B (**4**), physalin H (**5**), 5ß,6ß-epoxyphysalin C (**6**), and physalin
38 G (**7**), K (**8**), and D (**9**). It is worth noting that ~~aminophysalin B~~

compound **1** is the second

**Formatted:** Font: Bold

39 member of aminophysalin series ~~first reported in 2014~~, whereas ~~5ß, 6ß-epoxyphysalin~~
40 ~~C~~compound (**6**) was fully characterized for the first time~~previously reported only with tentative~~
41 ~~assignment based on the fragmentation pattern~~ *~~via~~* ~~UHPLC-QTOF-MS/MS analysis.~~
42 ~~Therefore, it is the first report of the isolation and validated structural characterization of 5ß,6ß-~~
43 ~~epoxyphysalin C (~~**~~6~~**~~)~~. The structures of compounds **1**-**9** were elucidated ~~through mass, UV, IR,~~
44 ~~and NMR~~ by spectroscopic techniques~~.~~ Whereas, the structural assignments of ~~The structures~~
45 ~~of~~ compounds **1** and **8** were also supported ~~deduced~~ by single-crystal X-ray diffraction studies.
46 Anti-leishmanial activity of isolated physlains

**1-9** was evaluated against *Leishmania major*

**Formatted:** Font: Bold

47 and *Leishmania tropica* promastegotes ~~(clinical isolates from Pakistani patients)~~*.* ~~Careful~~
48 ~~literature survey revealed that we have investigated the anti-leishmanial potential of all tested~~
49 ~~physalins~~ **~~1~~**~~-~~**~~9~~** ~~against~~ *~~L. tropica~~* ~~promastigotes for the first as~~ *~~L. tropica~~* ~~identified as the main~~
50 ~~cause of CL endemic in Pakistan.~~ Compounds **2**, **3**, and **5**-**7** (IC**50 =** 9.59 ± 0.27- 23.76 ± 1.10
51 μM) ~~appeared to be~~showed several fold more potent

activity against *L. tropca* than tested drug

**Formatted:** Font: Italic

52 miltefosine (IC**50 =** 42.75 ± 1.03 μ~~Μ~~m)**~~,~~** ~~the only orally available drug against cutaneous~~
53 ~~leishmaniasis~~ and pentamidine (IC50 = 27.20 ± 0.01 μM). Whereas compounds **2**, **3** and **5** (IC**50**

54	**=** 3.04 ± 1.12-3.76 ± 0.85 μM) were found to be potent anti-leishmanial agents against *L. major*,

55 several fold more active than tested standard miltefosine (IC**50 =** 25.55 ± 1.03 μM ~~27.20 ± 0.015~~
56 ~~μM~~) and pentamidine (IC**50 =** 27.20 ± 0.015 μM ~~25.55 ± 1.03 μM~~**)**. ~~Compounds~~ **~~2-9~~** ~~were~~
57 ~~checked for their anti-leishmanial potential against the Miltefosine MIL resistant~~ *~~Leishmania~~*
58 *~~topica~~* ~~strain.~~ Compounds **4** (IC50 = 74.65 ± 0.81 µM) and **7** (IC50 = 39.44 ± 0.65 µM) ~~were~~
59 ~~found to be~~also showed potent antileishmanial a~~gents~~tivity against the m~~M~~iltefosine-
60 unresponsive *Leishmania tropica* strain (MIL resistant) (~~M~~miltefosine IC50 = 169.55 ± 0.78
61 µM). Molecular docking and predictive binding studies indicated that these inhibitors may act
62 *via* targeting important enzymes of various metabolic pathways of the parasites~~, include ng~~
63 ~~phosphoglucose isomerase, and~~ *~~N~~*~~-myrositoyltransferase.~~.
64 **Key Words:** *Physalis minima,* physalins, leishmaniasis*, Leishmania major*, *Leishmania*
65 *tropica,* ~~3T3 (mouse fibroblast), BJ (human fibroblast)~~

##### 66 Introduction

67 Cutaneous leishmaniasis (CL), a neglected tropical disease (NTD), infects 700,000 to 1.2
68 million poorest people in over 98~~0~~ countries of Africa, Asia, Middle East and America
69 annually. Poverty, migration, poor hygiene, and malnutrition are the major risk factors
70 associated with endemicity of various forms of leishmaniasis in developing world [1-3]. About
71 70 types of sand flies are known to be responsible to transmit the *Leishmania* parasite in
72 geographically distributed ~~o~~Old (Asia, Africa, and Middle East) and ~~n~~New world *i.e.* western
73 hemisphere countries. Broadly they belong to p~~P~~hlebotomine and Lutzomyia genera. The
74 diagnosis of leishmaniasis, both parasitic (direct) and immunological (indirect), are often
75 challenging in low-resource countries [1]. Pakistan is among the endemic countries with CL
76 cases in almost every part of the country. In 2020, ~~A~~as per WHO Global Leishmaniasis
77 Surveillance Report, Afghanistan, Algeria, Brazil, Colombia, Iraq, Pakistan and Syria, are the
78 seven countries, each reported ~~represented~~ >6,000 CL cases, representing >80% of cases
79 globally [4]. CL is not a life-threatening infection but cause extreme psychological trauma, and
80 stigma as infected people pushed to be excluded from public life.
81 The available therapeutic options are now losing effectiveness due to the emergence of
82 resistance [5, 6]. Furthermore, these available drugs are expensive, and some have toxic effects
83 on liver and kidneys [7]. Medicinal plants have immense potential to yield compounds are
84 capable of treating many ailments. In recent days, pharmaceutical industries have reformed
85 their attention towards the plant-based medicine and formulation because of their least toxicity
86 and more efficacy [8-10]. *Physalis minima* Linn is a member of *Solanaceae* family, commonly

**Formatted:** Font: Italic

87 known as native gooseberry, and reported for anti-cancer, anti-diabetic, anti-oxidant, anti-
88 ulcer, analgesic, antipyretic, inhibition of amylase, lipase and alphaglucosidase enzymes, as
89 well as anti-gonorrhoeal, and anti-inflammatory properties [11-15]. Previous research on *P.*
90 *minima* led to the isolation of bioactive withanolides and physalins, which showed potent anti-
91 inflammatory and cytotoxic activities [16-19]. We have reported the *in vitro* anti-leishmanial
92 activity of the *Physalis minima* plant extracts and its pure constituents physalin B (**2**), 5ß,6ß-
93 epoxyphysalin B (**3**), and physalin H (**5**) against promastigotes of *L. major* for the first time
94 [20-22]. The presented study deals with the isolation, structure determination, and anti-
95 leishmanial effect of a new aminophysalin B (**1**) along with eight known physalins, physalin
96 B (**2**), 5ß,6ß-epoxyphysalin B (**3**), 5α-ethoxy-6ß-hydroxy-5,6-dihydrophysalin B (**4**), physalin
97 H (**5**), 5ß,6ß-epoxyphysalin C (**6**), and physalin G (**7**), K (**8**), and D (**9**) *in vitro* against
98 promastegotes of *L. tropica*. Compounds **1, 4**, **6**, **7, 8** and **9** were also evaluated against
99 promastegotes of *L. major* but no significant activity was observed. Moreover, the effect of
100

compounds **2-9** were also evaluated for their potential against the MIL resistant *Leishmania*

**Formatted:** Font: Bold

**Formatted:** Font: Italic

101 *topica*. These steroidal lactones **1**-**9** were also subjected to in-silico studies to predict their
102 mechanisms of action.

##### 103 Materials and Methods~~Experimental~~

104	**General**

105 Precoated TLC plates (silica gel, 20×20, 0.25 mm thick PF254, Merck, Germany) were used for
106 thin layer chromatography, and stained by spraying with Dragendroff’s and vanillin reagents.
107 Column chromatography was carried out on silica gel (70–230 mesh, Merck, Germany) [23].
108 Recycling preparative separation was performed on an HPLC JAI LC-908W (Japan) instrument,
109 equipped with GAIGEL-SIL, D-60-10, CHCl3-MeOH as the mobile phase, with UV detection
110 at 254 nm. JMS-600H mass spectrometer (JEOL, Japan) was used to record the mass spectra
111 (EI- and HREI-MS) in *m/z* (rel. %). Electrospray ionization mass spectra (ESI-MS) were
112 recorded on Amazon speed ion trap low-resolution mass spectrometer (Bruker
113 Daltonics, Germany) and high resolution electrospray ionization mass spectra (HRESI-MS)
114 were	measured	on	MaXis	II	ESI–QTOF	Ultra	High-Resolution	Mass
115 Spectrometer (Bruker Daltonics, Germany).Ultraviolet (UV) experiments were performed
116 in methanol on Hitachi U-3200 spectrophotometer (Hitachi, Japan). Infrared (IR) analysis was
117 performed in KBr on FT-IR-8900 (Shimadzu, Japan), and Bruker VECTOR 22
118 spectrophotometers (Bruker, France). UV Spectra (nm) were measured in methanol and
119 chloroform on Evolution 300 UV visible spectrophotometer. FT-IR-8900 spectrophotometer
120 was used to record IR spectra (cm^-1^) (JASCO, Japan). Optical rotations were measured in
121 methanol with a digital polarimeter JASCO P-2000 (JASCO, Japan) by using a 10 cm cell tube.
122 ^1^H- and ^13^C-NMR spectra were recorded on Avance NMR spectrometer at 500 and 75–150
123 MHz in CDCl3 or CD3OD, respectively (Bruker, Zurich, Switzerland). Standard pulse
124 sequences were used for DEPT, and 2D-NMR experiments. The ^1^H-NMR, ^13^C-NMR, COSY,
125 HMBC, HMQC, and NOESY spectra were recorded in deuterated chloroform, and methanol
126 on Bruker Avance spectrometers (Bruker, Zurich, Switzerland). X-Ray crystallography data
127

were collected on Bruker D8 venture diffractometer. Cu Kα radiation (1.54178 Å) were used

**Formatted:** Font: 12 pt

128 for X-ray diffraction data collection. SAINT program was employed for data integration and
129 reduction; Direct method was utilized for structure solution with full-matrix least square

method with aid of SHELXTL (SHELX2016/6). ~~[23~~, ~~24].~~

###### Plant

**Material**

**Formatted:** Font: 16 pt

**Formatted:** Font: 12 pt

132 Fresh plants (50 kg.) of *Physalin minima* (L. Var. indica), were collected from Karachi,
133 Pakistan, and identified by a plant taxonomists, Department of Botany, University of Karachi.
134 A voucher specimen (G. H. 68261) was deposited at the herbarium ~~of the Department of~~
135 ~~Botany, University of Karachi~~.

136	**Extraction and Isolation**

137 The air-dried plants (11 kg) were extracted with EtOH (100 L) for 10 days. After evaporation
138 of the solvent, a brownish residue (1.7 kg) was obtained that was allowed to be suspended in
139 distilled H2O (5 L), and defatted with petroleum ether (10 L) [24].. The defatted aqueous extract
140 was further extracted with CH2Cl2 (35 L). The CH2Cl2 extract was concentrated under reduced
141 pressure to obtain a greenish dried powder (85 g) that was subjected to column chromatography
142 and eluted with Pet ether: CH2Cl2, CH2Cl2, CH2Cl2: MeOH (10:90…90:10, 100, 99:1-97:3) to
143 afford four main fractions **A** (2.14 **g**), **B** (1.54 g), **C** (1.14 g), and **D** (24 g), respectively. The
144 repeated column chromatography of fraction **A** (2.14 **g**) afforded one major fraction **a** (300 mg,
145 petroleum: acetone 80: 20) which was further purified on recycling reverse phase HPLC to
146 obtain purified compound **2** (10 mg, CHCl3: MeOH-99:1, Flow rate: 4 mL/min, RT=16 min)
147 and **3** (60 mg, CHCl3: MeOH-99:1, Flow rate: 4 mL /min, RT = 18min). Fraction **B** (1.54 **g**)
148 yielded subfraction **b** (500 mg, petroleum: acetone 70: 30) which was further subjected to
149 recycling reverse phase HPLC to obtain compound **5** (10 mg, CHCl3: MeOH-99:1, Flow rate:
150 4 mL/min, RT=16 min). Fraction **C** (1.14 **g**) afforded one major subfraction **c** (petroleum:
151 acetone 80: 20) which yielded and purified compound **6** (5 mg, CHCl3: MeOH-99:1, Flow rate:
152 4 mL/min, RT=16 min) *via* recycling reverse phase HPLC. Fraction **D** (24 **g**) was subjected to
153 column chromatography and three major subfractions were obtained. Subtraction **d** (1.41)
154 (petroleum ether: acetone-55:45), **e** (1.0) (Petroleum ether: acetone-45:55) and **f** (20 g)
155 (petroleum: acetone-20:80) which were purified by using recycling reverse phase HPLC to
156 obtain compounds **1** (3 mg, CHCl3: MeOH-97:3, Flow rate: 3 mL/min, RT= 24 min) and **4** (4
157 mg, CHCl3: MeOH-96:4, Flow rate: 4 mL/min, RT= 24 min), **7** (10mg, CHCl3: MeOH-97:3,
158 Flow rate: 3 mL/min, RT= 30 min), **8** (6 mg, CHCl3: MeOH-97:3, Flow rate: 3 mL/min, RT=
159 24 min), and **9** (6 mg, CHCl3: MeOH-97:3, Flow rate: 3 mL/min, RT= 32 min).
160 Aminophysalin B (1)
161 Colorless solid (3 mg): [α] D= -65 (c = 0 .01, MeOH), **UV** (MeOH) λmax nm (log∈): 312 (3.2).

162	**IR** (KBr) νmax cm^-1^: 3463 (OH), 1732 (C=O), 1656 (C=C-C=O), **^1^H-NMR** (CDCl3, 500 MHz):

163	Table 1. **^13^C-NMR** (CDCl3, 175 MHz): Table 1. **HRESI-MS** *m/z*: 526.2059

164	[M+H]^+^(C24H31NO9+H requires 526.2072).

165 **Single-Crystal X-Ray Diffraction Data:** Empirical formula= C28 H31 NO9. H2O, Mr = 543.55,
166 Crystal system: orthorhombic, space group: P212121, Unit cell dimensions: **a** = 9.7870(4) Å, **b**

167	= 9.7870(4) Å, **c** = 16.3581(7) Å, Volume: 2412.66(18) Å^3^, Z=4, ρcalc = 1.496 Mg/m3, F(000)

168 1152, Crystal size: 0.900 x 0.060 x 0.030 mm, θ range for data collection: 5.266 to 68.135 deg.
169 Total 15,452 reflections were collected, out of which 4,364 were found unique (Rint= 0.0945).
170 Final R indices were R1 = 0.0551for [I>2σ (I)], wR2 = 0.1446, R indices were R1 = 0.0699,
171 wR2 = 0.1547 for all data, largest diff. peak and hole: 0.434 and -0.369 e. Å^-3^, CCDC No.

172	2177422.

173	**Physalin B** (**2**)

| 174 | Colorless solid (10 mg): [α] D= -257 (c = 0 .05, MeOH), **UV** (MeOH) λmax nm (log ∈): 230 |
| --- | --- |
| 175 | (3.6). **IR** (KBr) νmax cm^-1^ : 3410 (OH), 1748 (lactone), 1653 (C=C-C=O), **^1^H-NMR** (CDCl3, |
| 176 | 500 MHz): 1.19 (s, H3-28), 1.25 (s , H3-19), 1.95 (s, H3-21), 4.35 (t, overlapped *J*22,23 = 2.5 Hz, |
| 177 | H-22), 3.75 (d, *J*27,27 = 12.5 Hz, H2-27), 4.50 (dd overlapped, *J*27,27 = 13.5 Hz, *J*27,25 = 4.5, H2- |
| 178 | 27), 5.91 (dd, *J*2,3 = 10.0 Hz, *J*2, 4ß = 2 Hz, H-2), 6.77 (ddd, *J*3,2 = 10.0 Hz, *J*3,4a = 5.0 Hz, *J*3,4ß = |
| 179 | 2.5, H-3), 5.56 (d, *J*6,7 = 6.0 Hz, H-6), EIMS *m/z* (rel.int. %): 510 (M^+^), 492 (100.0), 477 (21.7), |
| 180 | 464 (48.0), 173 (46.2), 159 (70.9), 145 (26.1), 91 (26.6), 43 (18.1). |
| 181 | **5*β*, 6*β*-Epoxyphysalin B** (**3**) |
| 182 | White solid (60 mg), [α] D= -108 (*c* = 0 .05, MeOH), **UV** (MeOH) λmax nm (log ∈) : 230 (3.5). |
| 183 | **IR** (KBr) νmax cm^-1^ : 3422 (OH), 1778 (lactone), 1656 (C=C-C=O), **^1^H-NMR** (CDCl3, 500 |
| 184 | MHz): 1.24 (s , H3-28), 1.29 (s, H3-19), 1.93 (s, H3-21), 3.25 (d, *J*6α, 7β = 3.0 Hz, H-6), 4.52 (t, |
| 185 | overlapped *J*22,23 = 1.5 Hz, H-22), 3.74 (d, *J*27,27 = 13.5 Hz, H-27), 4.50 (dd overlapped, *J*27,27 = |
| 186 | 14.0 Hz, *J*27,25 = 4.5 Hz, H-27), 5.99 (dd, *J*2,3 = 10.5 Hz, *J*2, 4ß = 2.0 Hz, H-2), 6.84 (ddd, *J*3,2 = |
| 187 | 10.0 Hz, *J*3,4α= 5.0 Hz, *J*3,4ß = 2.5 Hz, H-3), EI-MS *m/z* (rel. int. %): 526 (6.7, M^+^), 498 (100.0), |
| 188 | 480 (15.7), 360 (16.6), 159 (17. 0), 133 (20.6), 91 (14.6), 55 (15.0). |
| 189 | **5*α*-Ethoxy-6ß-hydroxy-5,6-dihydrophysalin B** (**4**) |
| 190 | Colorless solid (4 mg): [α] D = -58 (*c* = 0 .05, MeOH), **UV** (MeOH) λmax nm (log ∈) : 230 (3.2). |
| 191 | **IR** (KBr) νmax cm^-1^ : 3405 (OH), 1787 (lactone), 1667 (C=C-C=O), **^1^H-NMR** (CDCl3, 500 |
| 192 | MHz): 0.98 (t, *J =* 7 Hz, CH3CH2O), 1.24 (s, H3-28), 1.50 (s, H3-19), 1.98 (s, H3-21), 4.00 (m, |
| 193 | H-6), 4.54 (t, overlapped *J*22,23 = 1.5, H-22), 3.76 (d, *J*27,27 = 13.5 Hz, H-27),4.49 (dd, *J*27,25 = |
| 194 | 4.5 Hz H-27), 5.85 (dd, *J*2,3 = 10.0 Hz, *J*2, 4ß = 2.5 Hz , H-2), 6.57 (ddd, *J*3,2 = 10.0 Hz, *J*3,4α = |
| 195 | 5.0 Hz, *J*3,4ß = 2.0 Hz, H-3), EI-MS *m/z* (rel. int. %): 572 (7.4, M^+^), 544 (10.8), 526 (24.8), 508 |
| 196 | (60.1), 454 (30.3), 171 (79.3), 147 (77.1), 133 (100.0), 109 (89.9), 91 (64.3), 55 (75.6). |
| 197 | **Physalin H** (**5**) |

198	Colorless solid (10 mg): [α] D = -260 (*c* = 0 .01, MeOH), **UV** (MeOH) λmax nm (log ∈) : 230

199	(3.8). **IR** (KBr) νmax cm^-1^ : 3426 (OH), 1778 (lactone), 1673 (C=C-C=O), **^1^H-NMR** (CDCl3,

200	500 MHz):1.35 (s, H3-19), 1.25 (s, H3-28), 1.99 (s, H3-21), 4.10 (d, *J*6α, 7β = 3.0 Hz, H-6), 4.54

201	(t, overlapped *J*22,23 = 2.5 Hz, H-22), 3.75 (d, *J*27,27 = 12.5 Hz, H2-27), 4.50 (dd, overlapped,

202	*J*27,27 = 13.5 Hz, *J*27,25 = 4.5 Hz, H2-27), 5.94 (dd, *J*2,3 = 10.0 Hz, *J*2, 4ß = 2.0 Hz, H-2), 6.71 (ddd,

203	*J*3,2 = 10.0 Hz, *J*3,4α= 5.0 Hz, *J*3,4ß = 2.5 Hz, H-4) **HRESI-MS** m/z: 563.1674 [M+H]^+^

204	(C28H31ClO10+H requires 563.1679).

205	**5*β*, 6*β*-Epoxyphysalin C** (**6**)

206	White solid (5 mg): [α] D= -168 (c = 0 .01, MeOH), **UV** (MeOH) λmax nm (log ∈) : 230 (3.1).

207	**IR** (KBr) νmax cm^-1^: 3427 (OH), 1782 (lactone), 1656 (C=C-C=O), **^1^H-NMR** (CH3OD, 500

208	MHz): Table 1. **^13^C-NMR** (CH3OD, 175 MHz): Table 1. **HRESI-MS** *m/z*: 527.1907

209	[C29H30O10+H; 527.1912]

210	**Physalin G** (**7**)

211	White solid (10 mg): [α] D= -92 (*c* = 0 .01, MeOH), **UV** (MeOH) λmax nm (log ∈) : 312 (5.8).**IR**

212	(KBr) νmax cm^-1^ : 3418 (OH), 1776 (lactone), 1622 (C=C-C=O), **^1^H-NMR** (CDCl3, 500

213	MHz):1.25 (s, H3-28), 1.93 (s, H3-19), 1.51 (s, H3-21), 4.49 (m, H-6), 4.52 (m, H-22), 3.74

214	(*J*27,27 = 13.0 Hz, H2-27), 4.50 (m, H2-27), 5.93 (d, *J*2,3 = 5.5 Hz, H-2), 6.82 (dd, *J*3,2 = 9.5 Hz,

215	*J*3,4 = 5.5 Hz, H-4), 6.03 (d, *J*4,3 = 6.0 Hz), **EIMS** *m/z* **(**rel. int. %): 526 (2.8, M^+^), 508 (55.1),

216	482 (79.8), 454 (40.1), 185 (38.9), 159 (27.6), 159 (98.6), 134 (67.1) 109 (43.5), 91 (27.0), 55

217	(32.4).

218 Physalin K (8)
219 Colorless solid (6 mg): [α] D= -266 (c = 0 .01, MeOH), **UV** (MeOH) λmax nm (log ∈) : 229

220	(3.0). **IR**(KBr) ν max cm^-1^ : 3407 (OH), 1775 (lactone), 1630 (C=C-C=O), **^1^H-NMR** (CDCl3,

221	500 MHz):1.12 (s , H3-28), 1.20 (s, H3-19), 1.90 (s, H3-21), 3.90 (d, *J*6α, 7β = 3.0, H-6), 4.55 (m,

| 222 | H-22), 3.76 (d, *J*27,27 = 13.5 Hz, H2-27), 4.49 (dd, *J*22,23α = 13.5 Hz, *J*22,23β = 5.0 Hz, H-22), 6.65 | | | | |
| --- | --- | --- | --- | --- | --- |
| 223 | (dd, *J*3,2 = 6.5 Hz, *J*3,4 = 8.0 Hz, H-3), 7.00 (dd, *J*4,2= 1.0 Hz, *J*4,3= 8.5 Hz, H-4), **HRESI-MS** | | | | |
| 224 | *m/z*: 559.1800 [C28H30O12+H; 559.1810]. | | | | |
| 225 | **Single-Crystal X-Ray Diffraction Data:** Empirical formula= C28 H30 O12. H2O. CH3OH, Mr | | | | |
| 226 | = 358.46, Crystal system: Monoclinic, space group: P21, Unit cell dimensions: **a** = 7.4821(5)Å, | | | | |
| 227 | **b** = 11.7099(7)Å, **c** = 15.2299(11)Å, Volume: 1327.52(15)Å^3^, Z=2, ρcalc = 1.522 Mg/m3, | | | | |
| 228 | F(000) 776, Crystal size: 0.270 x 0.130 x 0.010 mm, θ range for data collection: 4.773 to 68.237 | | | | |
| 229 | deg. A total of 17,151 reflections were collected, out of which 4,824 reflections were judged | | | | |
| 230 | observed (Rint = 0.1337), Final R indices were, R1 = 0.0521for [I>2σ (I)], wR2 = 0.1156, R | | | | |
| 231 | indices were R1 = 0.0816, wR2 = 0.1300 for all data, largest diff. peak and hole: 0.349and -- | | | | |
| 232 | 0.269e. Å^-3^, CCDC No. 2177423. | | | | |
| 233 | **Physalin D** (**9**) | | | | |
| 234 | Colorless solid (6 mg): [α] D= +78.4 (c = 0 .01, MeOH), **UV** (MeOH) λmax nm (log ∈) : 230 | | | | |
| 235 | (3.2).**IR** (KBr) νmax cm^-1^: 3377 (OH), 1788 (lactone), 1653(C=C-C=O),**^1^H-NMR** (CDCl3, 500 | | | | |
| 236 | MHz):1.25 (s, H3-28),1.29 (s, H3-28), 1.98 (s, H3-28), 3.76 (m, H-6), 4.53 (m, H-22), 3.74 (m, | | | | |
| 237 | H2-27), 4.50 (d, *J*27,25= 5.0 Hz, H2-27), 6.65 (ddd, *J*3,2 = 10.5, Hz, *J*3,4a = 4.5 Hz, *J*3,4ß = 2.5, H- | | | | |
| 238 | 3), 5.90 (dd, | *J*2,3 = | 10.5 Hz, | *J*2, 4ß | = 2.5 Hz, H-2), **HRESI-MS** [M+H]^+^ *m/z*: 545.2013 |
| 239 | [C28H32O11+H; 545.2004]. | | | | |

###### 240 ~~Anti-leishmanial Assay~~

241 *~~Leishmania major~~* ~~and~~ *~~Leishmania tropica~~* ~~(Clinical isolates) promastigotes were grown at 22~~
242 ~~± 25ºC in RPMI-1640 (Sigma) medium containing 10% of heat-inactivated (56 ºC for 30 min)~~
243 ~~fetal bovine serum. The promastigote culture in the logarithmic phase of growth was~~
244 ~~maintained, and the final concentration of parasites was adjusted to 2×10~~^6^ ~~parasites/mL. The~~
245 ~~test compound (1 mg) was dissolved in DMSO (50 μL), then the volume was made upto 1 Ml~~
246 ~~with RPMI-1640. Test compound (20 L) was added in the first well, which contained medium~~
247 ~~(180 μL), and was then serially diluted. Parasite suspension (100 L) was added into each well~~
248 ~~of the 96-well plates, and incubated at 21 ± 22 ºC for 72 h, in the presence and absence of~~
249 ~~amphotericin B (positive control). The experiments were carried out in duplicate, and the~~
250 ~~numbers of surviving parasites were counted in a Neubauer chamber. The 50%-inhibitory~~
251 ~~concentration (IC50) was determined with EZ-Fit software (Perrella Scientific).~~
252 ~~MIL resistant line:~~
253 ~~Promastigote cultures~~ *~~Leishmania tropica~~* ~~(Clinical isolates) were maintained at 26 °C in RPMI~~
254 ~~medium containing 15% FBS and 1% penicillin–streptomycin mixture.~~
255 ~~Generation of Miltefosine-unresponsive strain was carried under high MIL pressure by~~ *~~in vitro~~*
256 ~~passage with stepwise increase in the MIL concentration. At each step, parasites were cultured~~
257 ~~and passaged every 3–4 days at an initial concentration of 5x10~~^5^ ~~promastigotes/mL in order to~~
258 ~~achieve the stable growth as compared to the wild type isolate. Growth rates were measured~~
259 ~~for resistant populations and compared with the WT strain. Parasites were counted at an initial~~
260 ~~concentration of 5x10~~^5^ ~~parasites/mL and growth was measured daily using a Neubauer~~
261 ~~chamber until the population reached stationary phase. Furthermore, the fluorescence~~
262 ~~microscopic investigations via DAPI stain were also carried out to supplement the study and~~
263 ~~for confirmations~~
264

**Promastigote Growth Inhibition Assay**

**Formatted:** Font: 16 pt

265 For the estimation of particular concertation at which the any compounds caused 50%
266 inhibition (IC50) of resistant cell proliferation with respect to untreated controls, the MTT assay
267 was employed [25]. The effects of compounds against MIL- resistant line, the promastigotes
268 in their log phase were used in 96-well plates. 1 × 10^6^ resistant promastigotes were dispensed
269 and incubated with tested compounds at a range concentration of 200-10 μM at 27°C for 72 h.
270 After incubation of 72 h, MTT dye (3-(4,5-dimethylthiazol-2-yl)-2,5-diphenyl-2H-tetrazolium
271 bromide) was added and further incubate it for 3-4 h. ~~A~~amphotericin B was used as the positive
272 control, untreated promastigotes were used as negative control moreover DMSO control was
273 also added in the study as stock solution of the compounds were prepared in DMSO. All the
274 experiments were performed in triplicates. After completion of experiment the absorbance by
275 Multiskan ascent plate reader at 452 nm and percent inhibition was calculated by the following
276 formula
277 Cytotoxic concentration (%) = [100- (Absorbance of test)/ (Absorbance of solvent control)

278	×100] %

279 **MIL Resistant Line**
280 Promastigote cultures *Leishmania tropica* (Clinical isolates) were maintained at 26 °C in
281 RPMI-1640 medium containing 15% FBS and 1% penicillin–streptomycin mixture.
282 Generation of miltefosine-unresponsive strain was carried under high MIL pressure by *in vitro*
283 passage with stepwise increase in the MIL concentration. At each step, parasites were cultured
284 and passaged every 3–4 days at an initial concentration of 5x10^5^ promastigotes/mL in order to
285 achieve the stable growth as compared to the wild type isolate. Growth rates were measured
286 for resistant populations and compared with the WT strain. Parasites were counted at an initial
287 concentration of 5x10^5^ parasites/mL and growth was measured daily using a Neubauer
288 chamber until the population reached stationary phase. Furthermore, the fluorescence
289 microscopic investigations via DAPI stain were also carried out to supplement the study [26,

290	27].

291 Cytotoxicity against 3T3 Cell Lines
292 The cytotoxicity of the test compounds against cancer and normal cell lines was evaluated
293 through	MTT	(3-[4,	5-dimethylthiazole-2-yl]-2,	5-diphenyl-tetrazolium	bromide]
294 colorimetric assay in 96-well flat-bottomed microplates [

~~23~~

**Formatted:** Highlight

28]. The 3T3 (mouse fibroblast)

295 were cultured Dulbecco’s Modified Eagle Medium, supplemented with 5% of fetal bovine
296 serum (FBS), 100 IU/mL of penicillin and 100 µg/mL of streptomycin in 75 cm2 flasks, and
297

then incubated in 5% CO2 incubator at 37º~~o~~C. The growing cells were counted by using a haemocytometer, and diluted with the particular medium. The 5x10^4^ cells/mL of the cell culture

**Formatted:** Subscript

**Formatted:** Superscript

299 was prepared, and a sample (100 mL) introduced into each well. Medium was removed after
300 overnight incubation, and 200 µL of fresh medium was added with different concentrations of
301 compounds (1-30 µM). After 48 hours, 200 µL MTT (0.5 mg/mL) was added, and the
302 incubation was further continued for 4 hrs. After that time, 100 µL of DMSO was added to
303 each well. The absorbance was measured using a micro plate reader (Spectra Max Plus,
304 Molecular Devices, CA, USA) at 570 nm for the extent of MTT reduction to formazan within
305 the cells. The cytotoxicity was recorded as IC50 against cells by using Soft- Max Pro software.
306 Doxorubicin and cycloheximide were used as positive control. The experiments was run in
307 triplicate.

308	**Docking Studies**

309 In order to predict the tentative binding affinities and the possible mode of inhibition *via* these
310 compounds, molecular docking studies against various leishmanial metabolic enzymes that
311 have role in the growth and survival of the parasite were conducted.

**Formatted:** Font: 12 pt, Not Bold

312 Protein Preparation
313 For docking studies, the selected protein targets *i. e.,* pteridine reductase (PDB:1E92), pyruvate
314 kinase (PDB:3PP7), glyceraldehyde-3-phosphate dehydrogenase (PDB:1I32), phosphoglucose
315 isomerase	(PDB:1T10),	dihydroortate	dehydrogenase	(PDB:4EF8),	fructose-1,6-
316 bisphosphatase (PDB:5OFU), SAM-dependent methyltransferase (PDB:1XTP), mitochondrial
317 fumarate hydratase (PDB: 6MSO), *N*-myristoyltransferase (PDB: 6QDB), and MAP kinase
318

(PDB: 3UIB), were prepared by *Protein Preparation Wizard* in Maestro Schrödinger 12 [~~24,~~

**Formatted:** Highlight

319 ~~25~~29, 30]. During preparation, the missing hydrogens were added, and partial charges were
320 assigned using OPLS-3e force field. Hydrogens and heavy atoms were optimized by restrained
321 minimization.
322 Ligand P~~p~~reparation and D~~d~~atabase G~~g~~eneration
323 The 2D structures of compounds were converted to 3D structures, *via Ligprep* module in
324 Maestro Schrödinger 12 [

~~26~~

**Formatted:** Highlight

31]. *Ligprep* was used to correct the protonation, and ionization

325 states of the compounds, and assigned proper bond orders. Afterwards, the tautomeric and
326 ionization states was created for each ligand.
327

**Receptor Grid Generation and Molecular Docking**

**Formatted:** Font: 14 pt

328 The grid box was defined by selecting the co-crystallized molecules in the binding site of
329 abovementioned selected protein targets to keep the center of each docked ligand with same
330 dimensions of binding box. Rigid receptor docking protocol was run in standard precision (SP)
331

mode of Glide based on OPLS-3e force field [

32-34]. During the process of docking, the

**Formatted:** Highlight

332 protein was fixed, while ligands were flexible.

~~27-29~~

333 Molecular mechanics-generalized Born surface area (MM-GBSA) method in Prime was used
334 for rescoring the docked pose of ligand in the binding site of selected target protein [

~~30~~

**Formatted:** Highlight

35].

335 These poses were taken as inputs for the energy minimization of the protein-ligand complexes
336 (Ecomplex), free protein (Eprotein), and free ligands (Eligand). The binding free energy ∆Gbind was
337 determined according to the following equation:

338	𝛥𝐺𝑏𝑖𝑛𝑑 = 𝐸𝑐𝑜𝑚𝑝𝑙𝑒𝑥 (𝑚𝑖𝑛𝑖𝑚𝑖𝑧𝑒𝑑) − 𝐸𝑙𝑖𝑔𝑎𝑛𝑑 (𝑚𝑖𝑛𝑖𝑚𝑖𝑧𝑒𝑑) − 𝐸𝑟𝑒𝑐𝑒𝑝𝑡𝑜𝑟 (𝑚𝑖𝑛𝑖𝑚𝑖𝑧𝑒𝑑)

339

**Formatted:** Tab stops: Not at 0.06"

340

341

342

343

344

345

**Statistical Analysis**

Three replicates were used in each experiment, unless otherwise stated. All results were presented as means standard deviations. A one-way ANOVA was used to analyze statistical differences at a P-value 0.05 (95% confidence interval) in conjugation with Dunnett test using Graph pad prism version 9.4.1 (California. San Diego)

##### Results

**Formatted:** Font: 12 pt **Formatted:** Font: 12 pt **Formatted:** Font: 12 pt **Formatted:** Font: 12 pt **Formatted:** Font: 12 pt **Formatted:** Font: 12 pt **Formatted:** Font: 12 pt **Formatted:** Font: 12 pt **Formatted:** Font: 14 pt, Not Bold

ش

###### 346 Isolation and Structural Characterization

347 The whole plant of *P. minima* Linn. yielded nine compounds **1-9**, including a new
348 aminophysalin (**1**)**.** These compounds were characterized by spectroscopic techniques (Fig. 1)
349 Compound **1** was obtained as a colorless crystalline solid. The ESI-MS of 1 exhibited the
350 [M+H]^+^ at *m/z*: 526.2059 [C28H32NO9+H; 526.3072]. The IR spectrum showed a strong
351 absorption at 3463 cm^-1^, indicating the presence of OH groups. The absorption band at 1732
352 cm^-1^ is a characteristic absorption of physalin, appeared due to a five-membered lactone ring.
353 Furthermore, the IR absorption at 1656 cm^-1^ was assigned to an α,β-unsaturated ketone

[~~31-~~

**Formatted:** Highlight

354 ~~33~~36-38]. The^1^H-NMR spectrum (Table 1) of compound **1** showed three characteristic methyl
355 singlets, resonated at δ 1.54, 1.85, and 1.04 and assigned to H-19, H-21, and H-28 methyl
356 respectively. The appearance of three mutually coupled olefin carbon signals appeared at δ 6.00

357	(d, *J*2,3 = 10.0 Hz), 6.98 (dd, *J3*,4= 9.5 Hz, *J*3,4 = 6.0 Hz) and 6.12 (dd, *J*4,3= 5.5 Hz) indicated a

358 2-ene-1-one system in ring A. The presence of nitrogen containing bridge between C-15, and
359 C-27 (*i.e* entirely different from other physalin) was inferred from the appearance of a pair of

360	characteristic signals δ 4.17 (dd, *J*27,25 = 20.5 Hz, *J*27,27= 10.0 Hz) and δ 4.46 (*J*27,27 = 21) for

361

H2-27 [

39]. An overlapped signal was also appeared at δ 4.49 indicating the hydroxylation

**Formatted:** Highlight

362

at C-6 carbon [

40]. ^13^C-NMR spectra (Table 1) showed 28 carbon signals, assigned to eleven

~~36~~

**Formatted:** Highlight

363 quaternary, nine methine, five methylene, and three methyl carbons. There were four carbon

~~39~~

364 signals with chemical shift above δ 170, and assigned to two ketone carbonyls [δc 210.7 (C-1),
365 δc 208.8 (C-14)] and two ester carbonyls [δc 171.3 (C-18), δc 173.0 (C-26)]. The presence of
366 an additional signal at δ 164.9 (C-15), and downfield shift of C-27 at δ 47.7 suggesting the
367 nitrogen containing bridge between C-15 and C-27. The presence of carbonyl carbon at C-14
368 was supported by key HMBC correlations of δ 3.13 (H-8) and 2.52 (H-16) with δc 208.8 (C-
369 14) and 164.9 (C-15), respectively (Fig. 2). The HMBC correlation of H2-27 (δ 4.46, 4.17) with
370 164.9 (C-15) supported the nitrogen containing bridge. COSY correlation between H-25 and
371 H-27 was also supporting the proposed structure. The OH at C-6 was deduced on the basis of
372 HMBC correlations of H-4 (δ 6.12) with C-6 (δ 70.4) (Fig, 2).
373

The structure of compound **1** was unambiguously deduced on the basis of single-crystal X-ray

**Formatted:** Highlight

374 diffraction analysis (Figs. 4 and 5). The ORTEP diagram depicted that the molecule consists
375 of seven fused rings, *i.e.* rings A (C1-C5/C10), B (C5-C10), C(C8/C9/C11-17), D

376	(C13/C17/C18/C20/O9/O10), E (C16/C17/C20/C22-C24), F (C22-C26/O-9), and G (C-15/C-

377 16/C-24/C27-N1). Rings A and B transfused with each other through C5/C10 linkage and
378 exists in half chair and chair conformations, respectively. Ring C (C8/C9/C11-17) appeared in
379 twisted-boat-chair conformation with the cleavage of ether linkage between rings D and E as
380 appeared in other reported physalins. Tetrahydropyridine moiety *i.e.* ring G
381 (C15/C16/C24/C27N1) is completely different from reported physalin [36-37~~31, 32~~]. The
382 result showed that C6-OH and 28-CH3 were on the same side and assigned to be β oriented.
383 Wherears C13-OH, C17-OH and C21-CH3 were deduced to be α-oriented (Fig. 4).

In the

**Formatted:** Highlight

384

crystal lattice molecules found to be linked to form a three dimensional network (Fig. 5).

**Formatted:** Highlight

Previously reported, aminophysalin A [

39] was the first physalin having an unusual

**Formatted:** Highlight

386 structural feature in the with a nitrogen atom. On the basis of spectroscopic compound **1** was s

~~36~~

387 found to be the second member of aminophysalin series.

~~34, 35~~

388

~~36, 37~~

~~35~~

Compounds **2-5** were identified as previously reported physalin B (**2**) [

41, 42], 5β, 6β-

**Formatted:** Highlight

389

epoxyphysalin B (**3**) [

42], 5α-ethoxy-6ß-hydroxy-5,6-dihydrophysalin B (**4**) [

39, 43],

**Formatted:** Highlight

**Formatted:** Highlight

and physalin H (**5**) [~~38~~44] by comparison of the reported spectroscopic data. These compounds had first reported from *P. angulata*, *P. alkekengi,* and *P. minima*, respectively.

**Formatted:** Highlight

392 Compound **6** was isolated here first time from *Physalis minima.* Its structure was previously
393 reported only on the basis of tentative assignment based on the fragmentation pattern
394 studied through UHPLC-QTOF-MS/MS analysis *Physalis alkekengi L. var. franchetii* (Mast.)

~~41~~

395

[ 38]. Therefore, this is the first detailed report of the isolation and validated structure

**Formatted:** Highlight

396 characterization of 5ß,6ß-epoxyphysalin C (**6**). Compound **6** was obtained as a white solid. The
397 ESI-MS [M+H]^+^m/z: 527.1907 [C28H32NO9+H; 527.1912]. The IR spectrum showed strong
398 absorption at 3427, 1782, and 1656 indicating the presence of OH groups, five-membered
399 lactone ring and α,β-unsaturated ketone, respectively. The ^1^H-NMR spectrum (Table 1)
400 showed three characteristic methyl singlets at δ1.21, 1.92, and 1.58, and assigned to H3-19, H3-
401 21, and H3-28 methyl respectively. Two mutually coupled olefin proton signals, appeared at δ

402	5.99 (dd, *J*2,4 = 10.0 Hz, *J*2,3 = 2.5 Hz) and δ 6.83 (ddd, *J*2,3 = 3.0 Hz, *J*3,4 = 6.0 Hz, *J*3,4 = 10.0

403 Hz) indicated a 2-ene-1-one system in ring A. A one-proton downfield broad singlet at δ 3.24
404 (d, *J*=3.0 Hz) was assigned to the C-6 methine proton, geminal to oxy group. The assigned C-
405 6 proton at δ 3.24 was further supported by HMBC correlations with C-8 (δ 39.1) and C-7 (δ
406 26.4) (Fig. 2). HMBC correlation of H-3 (δ 6.83) with C-5 (δ 61.8) supported the presence of
407 an epoxide between C-5 and C-6. The low *J* value (*J*= 3.0 Hz) and comparison of the chemical
408 shift with a known physalin (5β,6β-epoxyphysalin B) suggested α-orientation of H-6 [32~~28~~].
409 Two characteristic signlets at δ 6.64 (s) and 5.72 (s) indicating the absence of oxygen
410 containing bridge between C-14 and C-27. An olefin at C-27 was inferred by HMBC

411	correlations of H2-27 (δ 6.64, δ 5.72) with C-26 (δ 163.7) and C-24 (δ 36.6). The ^13^C-NMR

412 data (Table 1) showed 28 carbon signals assigned to twelve quaternary, seven methine, six
413 methylene, and three methyl carbons. Signal at δ 64.4 was assigned to C-5, and indicated the
414 epoxidation at C-6. Two additional quaternary signals at δ 102.1 and 139.3 were assigned to
415 C-14 and C-24, respectively. NOESY correlation between the H-9 (δ 2.51) and H-6 (δ 3.24)
416 indicated a β epoxide (Fig. 3). Compound **6** was identified as 5β, 6β-epoxyphysalin C on the
417 basis of spectroscopic analysis.
418 Compounds **7** and **9** were identified as known physalins G (**7**) and physalin D (**9**) through
419 comparison of their reported spectroscopic data. [

~~39, 40~~

40, 45]

**Formatted:** Highlight

420 The first report for the isolation of physalin K (**8**) was from *Physalis minima* in 1980 [

~~39~~

**Formatted:** Highlight

40].

421 However the structure was extensively revised later on the basis of spectroscopic techniques in
422

1995 [

44]. We have studied the structural parameters of physalin K (**8**) on the basis of single-

**Formatted:** Highlight

423 crystal X-ray diffraction data and found it in good agreement with the revised structure.

~~41~~

424 Like other physalin, the molecular skeleton found to have rigid framework, consisting of eight
425 condensed rings. Two six membered rings *i.e.* rings A (C1/C2/C5/C10/O2/O3) and B (C2-
426 C5/O2/O3) linked together through epi-dioxy linkage at C2 and C5 adopts screw boat
427 conformation. Ring C (C5-C10) is transfused with ring A through C5/C10 bonds, and exists in
428 chair conformation. The eight membered ring D (O7/C8/C9/C11-C13/C17/C14) and adopts a
429 boat-chair conformation, connected with two *spiro*-fused rings E (C13/C17/C18/C20/O9/O10)
430 and F (C14-C17/O7) through O7/C13/C14/C17- /O7, while dimethyl substituted six membered
431 ring G (C-16/C-17/C-22---C-24) is connected with two lactone ring moieties of H (C14-
432

C16/C24-C27/O6) and I (C22-C26/O11/O12) along C16/C23-C25 linkage (Figs. 6 and 7).

**Formatted:** Highlight

###### 433 Anti-Leishmanial Activity

434 In order to evaluate the anti-leishmania activity, *Physalis minima* extracts (Ethanol/water, Pet
435 ether, Dichloromethane, and Ethyl acetate), active fractions (A-D), and pure physalins **1**-**9** were
436 incubated in various concentrations with axenic cultures of *L. tropica* and *L. major* in
437 comparison to three commercially available drugs, amphotericin B, pentamidine, and
438 miltefosine (Tables 2 and 3). The IC50 values (Table 3) indicated that chloro substituted
439 physalin H (**5**) was the most active member (IC**50 =** 9.59 ± 0.27 μM) of the series, followed by

440	physalins B (**2**, IC**50 =**13.33 ± 0.098 μM), physalins G (**7**, IC**50 =**19.49 ± 0.02 μM), and 5ß,6ß-

441 epoxyphysalin C (6, IC**50 =** 23.76 ± 1.10 μM) against *L. tropica* and showed more potent
442 activity than the standards pentamidine (IC**50 =** 27.20 ± 0.01 μM) and miltefosine (IC**50 =** 42.75
443 ± 1.03 μM). The anti-leishmanial activity of physalins B (**2**), 5ß,6ß-epoxyphysalin B (**3**) and
444 physalin H (**5**) was found in agreement with previous data reported by us [23] against *L. major,*
445 in comparison to amphotericin B. In current study, the comparison of anti-leishmanial activity
446 of physalins B (**2**), 5ß,6ß-epoxyphysalin B (**3**) and physalin H (**5**) against *L. major* include
447 comparison with
448 pentamidine and miltefosine. The results indicate that growth inhibition potential of physalins

449	B (**2,** IC**50 =** 3.04 ± 1.12 μM), 5ß,6ß-epoxyphysalin B (**3,** IC**50 =** 3.76 ± 0.85 μM) and physalin

450	H (**5,** IC**50 =** 3.34 ± 0.64 μM). These were several fold more potent than the standard drugs

451	pentamidine (IC**50 =** 27.20 ± 0.015 μM) and miltefosine (IC**50 =** 25.55 ± 1.03 μM). Compounds

452	**1**, **4,** and **8** appeared to be inactive against both *L. major* and *L. tropica* strains (Table 3).

453

454

455

456

457

458

459

460

461

462

463

Compounds **3**, **5**, and **6** showed low selectivity index (SI) values of 0.19, 0.119, and 0.007, respectively against *L. major* tested on 3T3 cell line. However, compound **2** showed SI value of 3.28. All these compounds showed <1 SI index against *L. tropica* cultured on 3T3 cells. Moreover, on the human fibroblast cell line (BJ), compounds **2**, **3**, **5**, and **6** showed ≥1 SI values of 3.5, 7.34, 3.11, and 1.19 against *L. major,* respectively. While compounds **3**, **5**, and **6** also showed SI values of 1.48, 1.08, and 4.20 against *L. tropica*. The SI value higher than 1 indicated that these compounds are theoretically more effective and safe drugs. However, compound 2 demonstrated a SI value of 0.8 on *L. tropica*. (Tables 5-8)

###### Anti-Leishmanial Activity against MIL resistant *L*. *tropica*

Furthermore, compounds **2-9** were also evaluated for their potential against the Miltefosine-

**Formatted:** Font: Bold **Formatted:** Font: Bold **Formatted:** Font: Bold **Formatted:** Font: Italic **Formatted:** Font: Bold **Formatted:** Font: Italic **Formatted:** Font: Bold **Formatted:** Font: Bold **Formatted:** Font: Bold **Formatted:** Font: Bold **Formatted:** Font: Italic **Formatted:** Font: Bold **Formatted:** Font: Bold **Formatted:** Font: Bold **Formatted:** Font: Italic **Formatted:** Font: Italic

464	unresponsive strain (MIL resistant) *Leishmania tropica*. Compounds **4** (IC50 = 74.65 ± 0.81

465	µM) and **7** (IC50 = 39.44 ± 0.65 µM) were found to be potent antileishmanial agents than the

466	standard Miltefosine (IC50 = 169.55 ± 0.78 µM). It is interesting to be noted that compound **4**

467	found to be inactive against clinical isolates of *L*. *major* and *L*. *tropica* (Table- 4~~3~~).

###### 468 Cytotoxicity of Physalin against BJ (Human Fibroblast) Cell Lines

469	Cytotoxicity *Physalis minima* extracts (Ethanol/water, Pet ether, Dichloromethane, and Ethyl

470	acetate), active fractions (A-D) ~~of~~, and pure physalins **2**-**9** were evaluated against normal

471	fibroblast (3T3) and BJ (human fibroblast) cells lines, and all tested compounds were found to

472	be non-cytotoxic in nature (Tables 5 and 6).

473	**Docking Studies**

474 Compounds with significant *in vitro* anti leishmanial activities were proceeded for docking
475 studies against various therapeutically important enzymes involved in different metabolic
476 pathways. Among various targets, compounds (**2**, **3**, **5**, **6**, **7**, and **9**) showed best binding
477 interaction with phosphoglucose isomerase and *N*-myrositoyltransferase. The docking scores
478 were in the range of -4.3 to -5.7 Kcal/mol (Fig 8), while the binding energies were in the range
479 of -11 to -75 Kcal/mol (Fig 9).

480	**Discussion:**

481 In last few decades, extensive research on medicinal plants have contributed towards increased
482 of natural products against health disorders globally. In continuation of our work to explore the
483 biological properties of medicinal plants, our group has previously reported the *in vitro* anti-
484 leishmanial effect of physalins B (**2**), 5ß,6ß-epoxyphysalin B (**3**) and physalin H (**5**), and other
485 isolated physalins from of *Physalis minima* against promastigotes of *L. major* [21, 22], in
486 comparison to amphotericin B. Amphotericin B is the drug of choice for the treatment of
487 various parasitic and fungal infections, including leishmaniasis. However, the clinical use of
488 amphotericin B is limited because of systematic toxicities, and lack of accessibility and
489

affordability [

47] The recommended least toxic, non-conventional amphotericin B

**Formatted:** Highlight

490 formulations, such as Fungizone®, AmBisome^®^, Abelcet^®^, Amphocil^®^ are expensive, and,

~~42~~

491

therefore, out of the reach of the poor patients [

48, 49]. Unfortunately, the first line

**Formatted:** Highlight

492 pentavalent antimonials are also not available in many leishmanial endemic countries.

~~43, 44~~

493 Therefore, alternatives such as pentamidine, and most recently miltefosine are the
494 recommended US-FDA approved drugs, prescribed by dermatologists for cutaneous
495 leishmaniasis. Therefore, in current study the inhibition abilities of isolated physalins towards
496 promastigotes of *L. major* and *L. tropica* were studied *in vitro,* and compared with
497
500
507

a~~A~~mphotericin B, pentamidine, and miltefosine. The physalins B (**2**), 5ß,6ß-epoxyphysalin B (**3**), and chloro substituted Physalin H (**5**) were previously reported by us as a promising candidates with significant abilities to reduce the growth of promastigotes of *L. major,* in comparison to amphoterin B [2~~3~~1]. The published results were further validated in present study and we further conclude that in addition to amphotericin B, these tested compounds also have several fold more inhibition potential against promastigotes of *L. major* than pentamidine (IC**50 =** 27.20 ± 0.015 μM), and miltefosine (IC**50 =** 25.55 ± 1.03 μM). In current study, the test compound also showed potent anti-leishmanial abilities against *L. tropica* promatigotes *in vitro,* and appeared to be several fold more active than tested standards pentamidine (IC**50 =**

27.20 ± 0.01 μM), and miltefosine (IC**50 =** 42.75 ± 1.03 μM). Activity of some selected compounds were also evaluated against the lab generated MIL-resistant line. Compound **2** showed SI value of >1 while compounds **3**, **5**, and **6** showed <1 SI against *L. major* on 3T3 cell line. Compounds **3**, **5**, and **6** showed >1 SI values against both *L. major* and *L. tropica* cultured on BJ cell line. However, compound **2** demonstrated >1 SI value only against *L. tropica*.

The alkylphosphocholine drug, miltefosine possess its potential against number of parasitic species and different cancer cells, along with activity against various pathogenic fungi and bacteria [50]. Knowledge of experimental MIL resistance in Leishmania is limited to defects in drug internalization (defective inward translocation of MIL) and increased drug efflux [51]. A major potential drawback in the use of miltefosine for the treatment of leisminiasis could be the relatively rapid generation of drug resistance in vitro [52]. According to Temmy Sunyoto et al, 2018 miltefosine is considered a breakthrough in the treatment, making it feasible to

**Formatted:** Font: Times New Roman, 12 pt, Font color: Auto

**Formatted:** Font: Times New Roman, 12 pt, Bold, Font color: Auto

**Formatted:** Font: Times New Roman, 12 pt, Font color: Auto

**Formatted:** Font: Times New Roman, 12 pt, Bold, Font color: Auto

**Formatted:** Font: Times New Roman, 12 pt, Font color: Auto

**Formatted:** Font: Times New Roman, 12 pt, Bold, Font color: Auto

**Formatted:** Font: Times New Roman, 12 pt, Font color: Auto

**Formatted:** Font: Times New Roman, 12 pt, Bold, Font color: Auto

**Formatted:** Font: Times New Roman, 12 pt, Font color: Auto

**Formatted:** Font: Times New Roman, 12 pt, Italic, Font color: Auto

**Formatted:** Font: Times New Roman, 12 pt, Font color: Auto

**Formatted:** Font: Times New Roman, 12 pt, Bold, Font color: Auto

**Formatted:** Font: Times New Roman, 12 pt, Font color: Auto

**Formatted:** Font: Times New Roman, 12 pt, Bold, Font color: Auto

**Formatted:** Font: Times New Roman, 12 pt, Font color: Auto

**Formatted:** Font: Times New Roman, 12 pt, Bold, Font color: Auto

**Formatted:** Font: Times New Roman, 12 pt, Font color: Auto

**Formatted:** Font: Times New Roman, 12 pt, Bold, Font color: Auto

**Formatted:** Font: Times New Roman, 12 pt, Font color: Auto

518 eliminate a regional disease, unfortunately the acquisition of resistance is of major concern.
519 Mechanisms that are responsible for the resistance acquisition in leishmaina parasite against
520 miltefosine includes reduction in drug uptake, increased efflux and alteration in permeability
521 of plasma membrane [53-57]. Due to this type of data and research highlights there is a
522 necessity to find out the alternative therapeutic options for leishmaniasis. During the study
523 presented the resistant strain was generated and libraries of compounds were evaluated against
524 the developed line. L. tropica MIL-unresponsive / resistant parasites were generated by using
525 the step wise selection of drug-miltefosine up to the concentration of 196 μM. No marked
526 difference in growth pattern were also analyzed between WT and MIL-resistant strain.
527 Compound **4** and **7** were observed as significantly potent as compared to the miltefosine,
528 revealing the potential of these natural compounds against drug –unresponsive strains.
529 However, these results of the current study demonstrated the promising anti-leishmanial
530 potential of physalins against *L. major* and *L. trpoica* promastigotes that need to be further
531 validated by *in vivo* studies as an anti-leishamanial agent for oral use.
532 Targeting the metabolic and biochemical pathways of leishmania is one of the most appropriate
533 therapeutic strategy. Following this rationale, compounds **2**, **3**, **5-7**, and **9** with significant *in-*
534 *vitro* antileishmanial activity were validated through docking studies against various metabolic
535 enzymes, in order to predict their mechanism of action. For this purpose, various enzymes from
536 major metabolic pathways were selected including glycolysis **(**pyruvate kinase,
537 glyceraldehyde-3-phosphate dehydrogenase, phosphoglucose isomerase), folate pathway
538 (pteridine	reductase),	gluconeogenesis	(fructose-1,6-bisphosphatase	cytosolic	and
539 mitochondrial fumarate hydratase), pyrimidine biosynthesis (dihydroortate dehydrogenase),
540 lipid metabolism (*N*-myrositoyl transferase), posttranslational modification (SAM-dependent
541 methyltransferase), and cell signalling (glycogen synthase kinase, MAP kinase). Docking
542 scores were observed in the range of -2 to -7 Kcal, while the binding energies were in the range
543 of -11 to -75 Kcal/mol. The results were then compared with the docking scores and binding
544 energies of antileishmanial clinical drugs including amphotericin B, pentamidine, and
545 miltefosin, and were found to be comparable with them. ~~(Fig 8 and 9)~~ The results further
546 indicated that the compounds might affect the glycolytic and lipid metabolic pathways by
547 targeting phospoglucose isomerase and *N*-myristoyltransferase, respectively.

548	**Conclusion**

549 Leishmaniasis, a neglected tropical disease is a rapidly growing infection in more than 98~~7~~
550 countries of the world. The presented study concluded with identification of natural steroidal
551 lactones i.e. physalins as potential candidates to be explore further against cutaneous
552 leishmaniasis. The complex structures of all natural compounds **1-9** were elucidated *via*
553 combined use of MS, IR, and NMR spectroscopic techniques. The assigned structures of
554 compounds **1**, and **8** were further supported by single-crystal X-ray diffraction studies. Anti-
555 leishmanial activities of isolated physlains was evaluated against *L. major* and *L. tropica .* The
556 literature survey revealed that all of the physalins possess as anti-leishmanial activity against *L.*
557 *tropica* (clinical isolate of Pakistan), reported for the first time. The promising results
558 of compounds 4 and 7 against the miltefosine-unresponsive *Leishmania tropica* strain (MIL
559 resistant) concluded the anti-leishmanial potential of tested compounds against resistant strain.
560 The compounds were also able to interact with therapeutically relevant enzymes of leishmania
561 including phosphoglucose isomerase, and *N*-myrositoyltransferase.

562	**~~Acknowledgments~~**

563	~~“The research leading to these results has, in part, received funding from UK Research and~~

564	~~Innovation via the Global Challenges Research Fund under grant agreement ‘A Global~~

565	~~Network for Neglected Tropical Diseases’, grant number MR/P027989/1.”~~

566

**Formatted:** RSC B02 Article Text, Left, Right: 0.2", Pattern: Clear

567	**References**

568	1. Mann S, Frasca K, Scherrer S, Henao-Martínez AF, Newman S, Ramanan P, Suarez

569	JA. A Review of Leishmaniasis: Current Knowledge and Future Directions. Curr Trop

570	Med Rep. 2021 Mar;17:1-12.

571 2. CDC.	Epidemiology	and	risk	factors.	[Available	from:	https://
572 [www.cdc.gov/parasites/leishmaniasis/epi.html]](http://www.cdc.gov/parasites/leishmaniasis/epi.html).
573 3. WHO.	Leishmaniasis	[Available	from:	https://[www.who.int/newsroom/fact-](http://www.who.int/newsroom/fact-)
574 sheets/detail/leishmaniasis].
575 4. Ruiz-Postigo JA, Jain S, Maia-Elkhoury AMAN, Valadas S, Warusavithana S, Osman
576 M, Lin Z, Beshah A, Yajima A, Gasimov E. Global leishmaniasis surveillance: 2019–
577 2020, a baseline for the 2030 roadmap. Weekly Epidemiological Record, 2021;96(35):

578	401+.

579 5. Haldar AK, Sen P, Roy S. Use of antimony in the treatment of leishmaniasis: current
580 status and future directions. Mol Biol Int. 2011;2011:571242.
581 6. Zahedifard F, Rafati S. Prospects for antimicrobial peptide-based immunotherapy
582 approaches in *Leishmania* control. Expert Rev Anti Infect Ther. 2018;16(6):461-469.
583 7. Sundar S, Chakravarty J. Antimony toxicity. Int J Environ Res Public Health. 2010

584	Dec;7(12):4267-77

585	8. Choudhary M, Kumar V, Malhotra H, Singh S. Medicinal plants with potential anti-

586	arthritic activity. J Intercult Ethnopharmacol. 2015;4(2):147-79.

587	9. Petrovska BB. Historical review of medicinal plants' usage. Pharmacogn Rev.

588	2012;6(11):1-5.

589	10. Šantić Ž, Pravdić N, Bevanda M, Galić K. The historical use of medicinal plants in

590	traditional and scientific medicine. Psychiatr Danub. 2017;29:787-792.

591	11. Vs A, Kp M, Krishna JG. Preliminary pharmacognostical and phytochemical

592	evaluation of *Physalis minima* Linn. (Ṭankārī). *J Pharmacogn Phytochem. 2018;7:*

593	1664-1669.

594	12. Patel T, Shah K, Jiwan K, Shrivastava N. Study on the antibacterial potential of physalis

595	minima linn. Indian J Pharm Sci. 2011;73(1):111-5.

596	13. Khan MA, Khan H, Khan S, Mahmood T, Khan PM, Jabar A. Anti-inflammatory,

597	analgesic and antipyretic activities of *Physalis minima* Linn. J Enzyme Inhib Med

598	Chem. 2009;24(3):632-7.

599	14. K. S., A. S. Screening of anti-diabetic activity of *Physalis Minima* Fruits. Int. J. Pharma

600	Bio Sci. (2020):11.

601 15. Chothani DL, Vaghasiya HUA. phyto-pharmacological overview on *Physalis minima*
602 Linn. Indian J Nat Prod Resour. 2012:3(4), 477-482.
603 16. Sun JL, Jiang YJ, Cheng L. Two new physalin derivatives from *Physalis alkekengi* L.
604 var. franchetii (Mast.) Makino. Nat Prod Res. 2021;35(2):203-206.
605 17. Zhang M, Jiang B, He X, Cao S, Ding L, Kang N, Chen L, Qiu F. New cytotoxic
606 withanolides from *Physalis minima*. Fitoterapia. 2020;146:104728.
607 18. Wu J, Zhang T, Yu M, Jia H, Zhang H, Xu Q, Gu Y, Zou Z. Anti-inflammatory
608 Withanolides from Physalis minima. ACS Omega. 2020;5(21):12148-12153.
609 19. Le Canh VC, Le Ba V, Thi Hai Yen P, Le Thi L, Thi Thuy Hoai P, Huu Dat TT, Thao
610 DT, Bach LG, Kim YH, Tuan Anh HL. Identification of potential cytotoxic inhibitors
611 from *Physalis minima*. Nat Prod Res. 2021; 35(12):2082-2085.
612 20. Choudhary	MI,	Yousuf	S,	Samreen,	Ahmed	S	Atta-ur-Rahman. New
613 leishmanicidalphysalins from *Physalis minima* , Nat Prod Res. 2007; 21:877-883.
614 21. Choudhary MI, Yousaf S, Ahmed S; Samreen, Yasmeen K; Atta-ur-Rahman.
615 Antileishmanial physalins from *Physalis minima*. Chem Biodivers. 2005;2(9):1164-73.
616 22. Choudhary MI, Yousaf S, Ahmed S; Samreen, Yasmeen K, Atta-ur-Rahman.
617 Discovery of leishmanicidal agents from medicinal plants. Pure Appl Chem. 2008;
618

80(8):1783-1790.

**Formatted:** English (United States)

619 23. Silver J, Let Us Teach Proper Thin Layer Chromatography Technique! J Chem Educ. 2020;97
620

(12): 4217-4219.

**Formatted:** Font: 12 pt, English (United States)

621 24. Sasidharan S, Chen Y, Saravanan D, Sundram KM, Yoga Latha L. Extraction, isolation
622 and characterization of bioactive compounds from plants' extracts. Afr J Tradit
623 Complement Altern Med. 2011;8 :1-10.
624 25. .Choudhary MI, Rizvi F, Siddiqui H, Yousuf S, Zafar H, Shaikh M. Microwave assisted
625 Biology-Oriented Drug Synthesis (BIODS) of new N,N′-disubstituted benzylamine
626 analogous of 4-aminoantipyrine against leishmaniasis – In vitro assay and in silico-
627 predicted molecular interactions with key metabolic targets. Bioorganic Chemistry,

628	2022;120: 105621

629 26. Hendrickx S, Inocencio da Luz RA, Bhandari V, Kuypers K, Shaw CD, Lonchamp J,
630 et al. Experimental induction of paromomycin resistance in antimony-resistant strains
631 of L. donovani: outcome dependent on in vitro selection protocol. PLoS neglected
632 tropical diseases. 2012;6: e1664.
633 ~~22.~~27.	Turner KG, Vacchina P, Robles-Murguia M, Wadsworth M, McDowell MA,
634 Morales MA Fitness and Phenotypic Characterization of Miltefosine-Resistant
635

Leishmania major. PLoS Negl Trop Dis 2015;9: e0003948.

**Formatted:** Font: Times New Roman, 12 pt

636 ~~23.~~28.	Dimas, K, Demetzos, C, Marsellos, M, Sotiriadou, R, Malamas, M,
637 Kokkinopoulos, D. Cytotoxic activity of labdane type diterpenes against human
638 leukemic cell lines in vitro. Planta. Med. 1998, 64, 208-211. DOI: 10.1055/s-2006-

639	957410

640 ~~24.~~29.	Schrödinger Release 2020-1: Protein Preparation Wizard; Epik, Schrödinger,
641 LLC, New York, NY, 2020; Impact, Schrödinger, LLC, New York, NY, 2020; Prime,
642 Schrödinger, LLC, New York, NY, 2020.
643 ~~25.~~30.	Sastry GM, Adzhigirey M. Day T. Annabhimoju R. Sherman W. Protein and
644 ligand preparation: parameters, protocols, and influence on virtual screening
645 enrichments. *J Comput Aided Mol Des*. 2013; 27(3):221-234.
646 ~~26.~~31.	Schrödinger Release 2020-1: LigPrep, Schrödinger, LLC, New York, NY,

647	2020.

648 ~~27.~~32.	Schrödinger Release 2020-1: Glide, Schrödinger, LLC, New York, NY, 2020.
649 ~~28.~~33.	Halgren TA, Murphy RB, Friesner RA, Beard HS, Frye LL, Pollard WT, Banks
650 JL. Glide: a new approach for rapid, accurate docking and scoring. 2. Enrichment
651 factors in database screening. J Med Chem. 2004; 47(7):1750-1759.
652 ~~29.~~34.	Friesner RA, Banks JL, Murphy RB, Halgren TA, Klicic JJ, Mainz DT, Shaw
653 DE. Glide: a new approach for rapid, accurate docking and scoring. 1. Method and
654 assessment of docking accuracy. J Med Chem. (2004);47(7):1739-1749.
655 ~~30.~~35.	Schrödinger Release 2020-1: Prime, Schrödinger, LLC, New York, NY, 2020
656 ~~31.~~36.	YANG Yan-Jun, SHA Cong-Wei, CHEN Mei-Guo, HU Ling. Extraction and
657 crystal structure of physalin B. *Chinese J Struc Chem*. 2014;33(5): 795-800.
658 ~~32.~~37.	Mangwala Kimpende P, Lusakibanza M, Mesia K, Tona L, Tits M, Angenot L,
659 Frédérich M, Van Meervelt L. Isolation, pharmacological activity and structure
660 determination of physalin B and 5β,6β-epoxyphysalin B isolated from Congolese
661 *Physalis angulata* L. *Acta Crystallogr C*. 2013 Dec 15; 69:1557-62.
662 38. Huang C, Xu Q, Chen C, Song C, Xu Y, Xiang Y, Feng Y, Ouyang H, Zhang Y, Jiang
663 H. The rapid discovery and identification of physalins in the calyx of Physalis alkekengi
664 L.var.franchetii (Mast.) Makino using ultra-high performance liquid chromatography-
665 quadrupole time of flight tandem mass spectrometry together with a novel three-step
666 data mining strategy. J Chromatogr A. 2014 Sep 26;1361:139-52.
667 39. Men RZ, Li N, Ding WJ, Hu ZJ, Ma ZJ, Cheng L. Unprecedent aminophysalin from
668 *Physalis angulata*. Steroids. 2014;88:60-5.
669 ~~33.~~40.	Row LR, Reddy KS, Sarma N, Matsuura T, & Nakashima R. New physalins

**Formatted:** Tab stops: 0.06", Left

670 from *Physalis angulata* and *Physalis lancifolia*. Structure and reactions of physalins D,
671

I, G and K. Phytochemistry, 1980; 19:1175-1181.

**Formatted:** Font: (Default) Times New Roman, 12 pt

672 ~~34.~~41.	Kawai M, Makino B, Taga T, Miwa Y, Yamamoto T, Furuta T, Yamamura H,
673 Butsugan Y, Ogawa K, Hayashi M. Crystal structures of 5α,6α-epoxy and 2,3-dihydro
674 derivatives of Physalin B, a 13,14-Seco-16,24-cyclosteroid, and Their 1H NMR
675 Spectral Analysis. Bull Chem Soc Jpn. 1994:67, 222-226.
676 ~~35.~~42.	Glotter E, Kirson I, Abraham A, Sethi PD, Subramanian SS. Steroidal
677 constituents of *Physalis minima* (solanaceae). J Chem Soc Perkin 1. 1975;(14):1370-4.
678

Men RZ, Li N, Ding WJ, Hu ZJ, Ma ZJ, Cheng L. Unprecedent aminophysalin from

36.

**Formatted:** Highlight

679 *~~Physalis angulata~~*~~. Steroids. 2014;88:60-5.~~
680 ~~37.~~43.	Kawai M, Makino B, Yamamura H, Butsugan Y. (1996). Upon ‘physalin L’
681 isolated from Physalis minima. Phytochemistry, 43, 661-663.
682 ~~38.~~44.	Bunsho Makino, Masao Kawai, Toichi Ogura, Masaki Nakanishi, Hatsuo
683 Yamamura, and Yasuo Butsugan, Structural Revision of Physalin H Isolated from
684 *Physalis angulate*. Journal of Natural Products 1995;58(11):1668-1674,
685

Row LR, Reddy KS, Sarma N, Matsuura T, & Nakashima R. New physalins from

39.

**Formatted:** Highlight

686 *~~Physalis angulata~~* ~~and~~ *~~Physalis lancifolia~~*~~. Structure and reactions of physalins D, I, G~~

687	~~and K. Phytochemistry, 1980; 19:1175-1181.~~

688	~~40.~~45.	Januário AH, Filho ER, Pietro RC, Kashima S, Sato DN, França SC.

689	Antimycobacterial physalins from *Physalis angulata* L. (Solanaceae). Phytother Res.

690	2002:16(5):445-8.

691 ~~41.~~46.	Makino B, Kawai M, Iwata Y, Yamamura H, Butsugan Y, Ogawa K, Hayashi
692 M. Physalins Possessing an Endoperoxy Structure from *Physalis alkekengi* var.
693 *francheti*. Structural Revision of Physalin K. Bull Chem Soc Jpn. 1995; 68:219-226.
694 ~~42.~~47.	de Menezes JP, Guedes CE, Petersen AL, Fraga DB, Veras PS. Advances in
695 development of new treatment for leishmaniasis. *Biomed Res Int.* 2015;2015:815023.
696 ~~43.~~48.	Clemons KV, Stevens DA. Comparative efficacies of four amphotericin B
697 formulations--Fungizone, amphotec (Amphocil), AmBisome, and Abelcet--against
698 systemic murine aspergillosis*. Antimicrob Agents Chemother.* 2004;48(3):1047-50.
699 49. Cuddihy G, Wasan EK, Di Y, Wasan KM. The development of oral amphotericin B to
700 treat systemic fungal and parasitic infections: has the myth been finally realized?
701

*Pharmaceutics*. 2019;11(3):99.

**Formatted:** English (United States)

702 50. Dorlo TP, Balasegaram M, Beijnen JH, de Vries PJ. Miltefosine: a review of its
703 pharmacology and therapeutic efficacy in the treatment of leishmaniasis. The Journal
704 of antimicrobial chemotherapy. 2012;67: 2576–97.
705 51. Perez-Victoria FJ, Sanchez-Canete MP, Seifert K, Croft SL, Sundar S, Castanys S, et
706 al. Mechanisms of experimental resistance of Leishmania to miltefosine: Implications
707 for clinical use. Drug Resist Updat. 2006;9 :26–39.
708 52. Varela-M RE, Villa-Pulgarin JA, Yepes E, Müller I, Modolell M, Muñoz DL, et al. In
709 Vitro and In Vivo Efficacy of Ether Lipid Edelfosine against Leishmania spp. and SbV-
710 Resistant Parasites. PLoS Negl Trop Dis 2012;6: e1612.
711 53. Perez-Victoria FJ, Castanys S, Gamarro F. Leishmania donovani resistance to
712 miltefosine involves a defective inward translocation of the drug. Antimicrob Agents

713	Chemother 2003;47: 2397–2403.

714 54. Seifert K, Matu S, Pe´rez-Victoria FJ, Castanys S, Gamarro F, Croft SL.
715 Characterisation	of	Leishmania	donovani	promastigotes	resistant	to
716 hexadecylphosphocholine (miltefosine). Int J Antimicrob Agents 2003;22: 380–387.
717 55. Kulshrestha A, Sharma V, Singh R, Salotra P. Comparative transcript expression
718 analysis of miltefosine-sensitive and miltefosine-resistant Leishmania donovani.

719	Parasitol Res 2014;113:1171–1184.

720	56. Sanchez-Cañete MP, Carvalho L, Victoria-Pe´rez FJ, Gamarro F, Castanys S. Low

721	plasma membrane expression of the miltefosine transport complex renders Leishmania

722	braziliensis refractory to the drug. Antimicrob Agents Chemother 2009;53: 305–313.

723	31.

724	57. Mondelaers A, Sanchez-Cañete MP, Hendrickx S, Eberhardt E, Garcia-Hernandez R,

725	Lachaud L, et al. Genomic and molecular characterization of miltefosine resistance in

726	Leishmania infantum strains with either natural or acquired resistance through

727	experimental selection of intracellular amastigotes. PLoS One 2016; 11: e0154101.

728

**Formatted:** Normal, Indent: Left: 0.25", No bullets or numbering

**Formatted:** Font: Times New Roman, 12 pt

729

730

731

732

~~44.~~

**Table 1:** ^1^H-NMR ^13^C-NMR chemical shift data of compounds **1** and **6** (δ in ppm, *J* in Hz)

| **C. No.** | **_1_a** | **_1_b** | **_6_a** | **_6_b** |
| --- | --- | --- | --- | --- |
| **1** | - | 208.8 | - | 204.4 |
| **2** | 6.00 (d, *J* 2,3 = 10.0) | 119.8 | 5.99 (dd, *J*2,3 = 10.0, *J*2,4 = 2.5) | 145.5 |
| **3** | 6.98 (dd, *J* 3,4 = 9.5, *J* 3,4 = 6.0) | 140.9 | 6.83 (ddd, *J*3,2 = 10.0, *J*3,4β = 2.5, *J*3,4α = 6.0) | 128.6 |
|  | 6.12 (d, *J* 3,4 = 5.5) | 125.5 | 2.93, m; 2.04, m | 32.9 |
| **5** | - | 155.6 | - | 61.8 |

| 733 | **^a^** 500 MHz, CDCl3 |
| --- | --- |
| 734 |  |
| 735 | **Table 2: Anti-Leishmanial Activity of Extracts and Fraction against** |
| 736 | ***L. major* and *L. tropica.*** |

| **6** | 4.49, m | 70.4 | 3.24 (d, *J*3,4 = 2.5) | 64.6 |
| --- | --- | --- | --- | --- |
| **7** | 2.10, m; 2.53, m | 29.4 | 2.35, m; 1.71, m | 29.8 |
| **8** | 3.13, m | 39.4 | 2.22, m | 39.1 |
| **9** | 2.57, m | 34.3 | 2.51, m | 35.5 |
| **10** | - | 52.0 | - | 49.8 |
| **11** | 2.35, m; 1.65, m | 28.2 | 2.26, m; 1.14, m | 22.4 |
| **12** | 2.54, m; 2.04, m | 29.5 | 2.64, m; 1.76, m | 26.4 |
| **13** | - | 79.4 | - | 79.7 |
| **14** | - | 210.7 | - | 102.1 |
| **15** | - | 171.5 | - | 210.8 |
| **16** | 2.52, m | 56.5 | 2.50, m | 54.6 |
| **17** | - | 83.3 | - | 81.8 |
| **18** | - | 173.0 | - | 171.8 |
| **19** | 1.54, s | 23.0 | 1.21, s | 14.3 |
| **20** | - | 80.0 | - | 82.7 |
| **21** | 1.85, s | 21.7 | 1.92, s | 20.9 |
| **22** | 4.50, m | 77.3 | 4.58, (dd, *J*22,23 = 4.5, *J*22,23 = 2) | 76.8 |
| **23** | 2.05, m; 1.97, m | 31.6 | 1.99, m; 2.11, m | 31.7 |
| **24** | - | 29.1 | - | 36.6 |
| **25** | 2.36, m | 42.2 | - | 139.3 |
| **26** | - | 164.9 | - | 163.7 |
| **27** | 4.17 (dd, *J*27,27 = 10.0, *J*27,25 = 20.5)  4.46 (*J*27,27 = 21) | 47.7 | 4.20 (6.64, s; 5.72, s) | 133.3 |
| **28** | 1.04, s | 28.2 | 1.58, s | 27.3 |

|  |  | ***~~L. major~~*** | ***~~L. tropica~~*** |
| --- | --- | --- | --- |
|  |  | **~~IC50 (~~*~~μ~~*~~g/ml ± SD)~~** | **~~IC50 (~~*~~μ~~*~~g/ml ± SD)~~** |
| **~~Extracts~~** | ~~Ethanol/water~~ | ~~>100~~ | ~~>100~~ |
|  | ~~Pet ether~~ | ~~>100~~ | ~~>100~~ |
|  | ~~Dichloromethane~~ | ~~19.01±0.7~~ | ~~19.53±1.3~~ |
|  | ~~Ethyl acetate~~ | ~~>100~~ | ~~>100~~ |

| **~~Fractions~~** | ~~A~~ | ~~6.67±0.1~~ | ~~10.16±2.0~~ |
| --- | --- | --- | --- |
|  | ~~B~~ | ~~9.95±1.9~~ | ~~6.41±0.074~~ |
|  | ~~C~~ | ~~6.30±0.018~~ | ~~6.34±0.045~~ |
|  | ~~D~~ | ~~6.37±0.015~~ | ~~6.37±0.015~~ |
| **~~Standard drugs~~** | ~~Amphotericin~~ | ~~3.14 ± 0.04~~ | ~~3.16 ± 0.04~~ |
|  | ~~Pentamidine~~ | ~~9.25 ± 0.005~~ | ~~9.25 ± 0.005~~ |
|  | ~~Miltefosine~~ | ~~10.4 ± 0.42~~ | ~~17.4 ± 0.42~~ |

737

|  |  | *L. major* | | | | *L. tropica* | | | |
| --- | --- | --- | --- | --- | --- | --- | --- | --- | --- |
|  |  | **IC50**  **(*μ*g/ml**  **± SD)** | **p-value** Amphote ricin | **p-value** Pentami dine | **p- value** Miltefo sine | **IC50**  **(*μ*g/ml**  **± SD)** | **p-value** Amphote ricin | **p-value** Pentami dine | **p- value** Miltefo sine |
|  | Ethanol/wa ter | >100 | - |  |  | >100 | - |  |  |
| Extra | Pet ether | >100 | - |  |  | >100 | - |  |  |
| cts | Dichlorom ethane | 19.01±  0.7 | <0.0001 | <0.0001 | <0.000  1 | 19.53±  1.3 | <0.0001 | <0.0001 | 0.0021 |
|  | Ethyl acetate | >100 | - |  |  | >100 | - |  |  |
|  | A | 6.67±0.  1 | <0.0001 | <0.0001 | <0.000  1 | 10.16±  2.0 | <0.0001 | 0.0568 | <0.000  1 |
| Fracti | B | 9.95±1.  9 | <0.0001 | 0.8898 | 0.5084 | 6.41±0.  074 | <0.0001 | 0.0001 | <0.000  1 |
| ons |  |  |  |  |  |  |  |  |  |
|  | C | 6.30±0.  018 | <0.0001 | <0.0001 | <0.000  1 | 6.34±0.  045 | <0.0001 | <0.0001 | <0.000  1 |
|  | D | 6.37±0.  015 | <0.0001 | <0.0001 | <0.000  1 | 6.37±0.  015 | <0.0001 | 0.0001 | <0.000  1 |
|  | Amphoteri | 3.14 ± |  |  |  | 3.16 ± |  |  |  |
|  | cin | 0.04 |  |  |  | 0.04 |  |  |  |
| Stand ard drugs |  |  |  |  |  |  |  |  |  |
|  | Pentamidin e | 9.25 ±  0.005 |  |  |  | 9.25 ±  0.005 |  |  |  |
|  | Miltefosine | 10.4 ±  0.42 |  |  |  | 17.4 ±  0.42 |  |  |  |

738

739

740

741

742	Table 3: Anti-Leishmanial Activity of Compounds 1-9 against

743	***L. major* and *L. tropica.***

|  |  | ***~~L. major~~*** | ***~~L. tropica~~*** |
| --- | --- | --- | --- |
|  | **~~Compounds~~** | **~~IC50~~**  **~~(~~*~~μ~~*~~M± SD)~~** | **~~IC50~~**  **~~(~~*~~μ~~*~~M± SD)~~** |
| **~~Pure~~ ~~Compounds~~** | **1** | ~~inactive~~ | ~~inactive~~ |
|  | **2** | ~~3.04 ± 1.12~~ | ~~13.33 ± 0.098~~ |
|  | **3** | ~~3.76 ± 0.85~~ | ~~18.53 ± 0.28~~ |
|  | **4** | ~~inactive~~ | ~~inactive~~ |
|  | **5** | ~~3.34 ± 0.64~~ | ~~9.59 ± 0.27~~ |
|  | **6** | ~~84.79 ± 1.56~~ | ~~23.76 ± 1.10~~ |
|  | **7** | ~~52.28 ± 1.18~~ | ~~19.49 ± 0.02~~ |
|  | **8** | ~~inactive~~ | ~~Inactive~~ |
|  | **9** | ~~inactive~~ | ~~55.24± 0.01~~ |
| **~~Standard~~ ~~drugs~~** | ~~Amphotericin~~ | ~~3.39 ± 0.043~~ | ~~3.42 ± 0.04~~ |
|  | ~~Pentamidine~~ | ~~27.20 ± 0.015~~ | ~~27.20 ± 0.01~~ |
|  | ~~Miltefosine~~ | ~~25.55 ± 1.03~~ | ~~42.75 ± ± 1.03~~ |

744

**Formatted:** Width: 11.69", Height: 8.27", Header distance from edge: 0.49", Footer distance from edge: 0.49"

745	**Table**

746

747	Table 3: Anti-Leishmanial Activity of Compounds 1-9 against

748	***L. major* and *L. tropica.***

749

| Compounds | *L. major* | | | | *L. tropica* | | | |
| --- | --- | --- | --- | --- | --- | --- | --- | --- |
|  | **IC50 (*μ*M± SD)** | **p-value**  Amphotericin | **p-value**  Pentamidine | **p-value**  Miltefosine | **IC50 (*μ*M± SD)** | **p-value**  Amphotericin | **p-value**  Pentamidine | **p-value**  Miltefosine |
| 1 | Inactive | - | - | - | Inactive | - | - | - |
| 2 | 3.04 ± 1.12 | 0.9001 | <0.0001 | <0.0001 | 13.33 ± 0.098 | <0.0001 | <0.0001 | <0.0001 |
| 3 | 3.76 ± 0.85 | 0.9997 | <0.0001 | <0.0001 | 18.53 ± 0.28 | <0.0001 | <0.0001 | <0.0001 |
| 4 | Inactive | 0.9999 | <0.0001 | <0.0001 | Inactive | <0.0001 | <0.0001 | <0.0001 |
| 5 | 3.34 ± 0.64 | <0.0001 | <0.0001 | <0.0001 | 9.59 ± 0.27 | <0.0001 | 0.0009 | <0.0001 |
| 6 | 84.79 ± 1.56 | <0.0001 | <0.0001 | <0.0001 | 23.76 ± 1.10 | <0.0001 | <0.0001 | <0.0001 |
| 7 | 52.28 ± 1.18 | 0.9001 | <0.0001 | <0.0001 | 19.49 ± 0.02 | <0.0001 | <0.0001 | <0.0001 |
| 8 | Inactive | - | - | - | Inactive | - | - | - |
| 9 | Inactive | - | - | - | 55.24± 0.01 | <0.0001 | <0.0001 | <0.0001 |
| **Amphotericin** | 3.39 ± 0.043 |  |  |  | 3.42 ± 0.04 |  |  |  |
| **Pentamidine** | 27.20 ± 0.015 |  |  |  | 27.20 ± 0.01 |  |  |  |
| **Miltefosine** | 25.55 ± 1.03 |  |  |  | 42.75 ± ± 1.03 |  |  |  |

750

34

751	4: Anti-Leishmanial Activity of Compounds 2-9 against the MIL

752	**Resistant *L. tropica*.**

|  |  | **MIL Resistant**  ***L. tropica*** |
| --- | --- | --- |
|  | **Compounds** | **IC50**  **(*μ*M± SD)** |
| **Pure Compounds** | **2** | inactive |
|  | **3** | inactive |
|  | **4** | 74.65 ± 0.81 |
|  | **5** | inactive |
|  | **6** | inactive |
|  | **7** | 39.44 ± 0.65 |
|  | **8** | Inactive |
|  | **9** | inactive |
| **Standard drugs** | Miltefosine | 169.55 ± 0.78 |

753

754

755

756

757

758

759

760

761

762

763

764

765

766

767

35

768	Table 5: Selectivity Index of Pure Compounds (BJ Fibroblast Cell line)

**Formatted:** Width: 11.69", Height: 8.27", Header distance from edge: 0.49", Footer distance from edge: 0.49"

|  | **Samples** | **BJ cell line CC50 (*μ*M± SD)** | ***L. major***  **IC50 (*μ*M± SD)** | ***L. tropica***  **IC50 (*μ*M± SD)** | ***L. major***  **SI (CC50/ IC50)** | ***L. tropica***  **SI (CC50/ IC50)** |
| --- | --- | --- | --- | --- | --- | --- |
| **Extracts** | Ethanol/water | >100 | >100 | >100 | - | - |
|  | Pet ether | >100 | >100 | >100 | - | - |
|  | Dichloromethane | 26.5±1.1 | 19.01±0.7 | 19.53±1.3 | 1.39 | 1.35 |
|  | Ethyl acetate | >100 | >100 | >100 | - | - |
| **Fractions** | Fraction-A | 10.4 ± 1.0 | 6.67±0.1 | 10.16±2.0 | 1.49 | 1.02 |
|  | Fraction-B | >100 | 9.95±1.9 | 6.41±0.074 | - | - |
|  | Fraction-C | >100 | 6.30±0.018 | 6.34±0.045 | - | - |
|  | Fraction-D | >100 | 6.37±0.015 | 6.37±0.015 | - | - |

769

770	Table 6: Selectivity Index of Crude Fractions (BJ Fibroblast Cell line)

771

772

773

| **Samples** | **BJ cell line CC50 (*μ*M± SD)** | ***L. major***  **IC50 (*μ*M± SD)** | ***L. tropica***  **IC50 (*μ*M± SD)** | ***L. major***  **SI (CC50/ IC50)** | ***L. tropica***  **SI (CC50/ IC50)** |
| --- | --- | --- | --- | --- | --- |
| **1** | Non cytotoxic | Inactive | Inactive | - |  |
| **2** | 10.8 ± 1.5 | 3.04 ± 1.12 | 13.33 ± 0.098 | 3.5 | 0.8 |
| **3** | 27.6 | 3.76 ± 0.85 | 18.53 ± 0.28 | 7.34 | 1.48 |
| **4** | 14.2 ± 0.8 | Inactive | Inactive | - | - |
| **5** | 10.4 ± 1.0 | 3.34 ± 0.64 | 9.59 ± 0.27 | 3.11 | 1.08 |
| **6** | >100 | 84.79 ± 1.56 | 23.76 ± 1.10 | 1.19 | 4.20 |
| **7** | >100 | 52.28 ± 1.18 | 19.49 ± 0.02 | - | - |
| **8** | >100 | Inactive | Inactive | - | - |
| **9** | >100 | Inactive | 55.24± 0.01 | - | - |
| **Amphotericin** | 16.059 | 3.39 ± 0.043 | 3.42 ± 0.04 | 4.37 | 4.69 |

774

775

776

777

778

779

780

781	Table 7: Selectivity Index of Pure Compounds (3T3 Mouse Fibroblast Cell line)

| **Samples** | **3T3 Cell line CC50 (*μ*M± SD)** | ***L. major***  **IC50 (*μ*M± SD)** | ***L. tropica***  **IC50 (*μ*M± SD)** | ***L. major***  **SI (CC50/ IC50)** | ***L. tropica***  **SI (CC50/ IC50)** |
| --- | --- | --- | --- | --- | --- |
| **1** | - | Inactive | Inactive | - | - |
| **2** | 10 ± 0.4 | 3.04 ± 1.12 | 13.33 ± 0.098 | 3.28 | 0.007 |
| **3** | 0.72 ± 0.5 | 3.76 ± 0.85 | 18.53 ± 0.28 | 0.19 | 0.038 |
| **4** | 19.6± 0.8 | Inactive | Inactive | - | - |
| **5** | 0.4 ± 0.07 | 3.34 ± 0.64 | 9.59 ± 0.27 | 0.119 | 0.041 |
| **6** | 0.6 ± 0.04 | 84.79 ± 1.56 | 23.76 ± 1.10 | 0.007 | 0.025 |
| **7** | Non cytotoxic | 52.28 ± 1.18 | 19.49 ± 0.02 | - | - |
| **8** | Non cytotoxic | Inactive | Inactive | - | - |
| **9** | Non cytotoxic | Inactive | 55.24± 0.01 | - | - |

782

783

784

785

786

787	Table 8: Selectivity Index of Pure Compounds (3T3 Mouse Fibroblast Cell line)

|  | **Samples** | **3T3 cell line CC50 (*μ*M± SD)** | ***L. major***  **IC50 (*μ*M± SD)** | ***L. tropica***  **IC50 (*μ*M± SD)** | ***L. major***  **SI (CC50/ IC50)** | ***L. tropica***  **SI (CC50/ IC50)** |
| --- | --- | --- | --- | --- | --- | --- |
| **Extracts** | Ethanol/water | >100 | >100 | >100 | - | - |
|  | Pet ether | >100 | >100 | >100 | - | - |
|  | Dichloromethane | 4.5 ± 0.5 | 19.01±0.7 | 19.53±1.3 | 0.23 | 0.23 |
|  | Ethyl acetate | >100 | >100 | >100 | - | - |
| **Fractions** | Fraction-A | 12.2 ± 1.5 | 6.67±0.1 | 10.16±2.0 | 7.30 | 1.20 |
|  | Fraction-B | 4.8 ± 1.6 | 9.95±1.9 | 6.41±0.074 | 0.48 | 0.74 |
|  | Fraction-C | 2.7 ± 0.3 | 6.30±0.018 | 6.34±0.045 | 0.42 | 0.42 |
|  | Fraction-D | 3.3 ± 0.3 | 6.37±0.015 | 6.37±0.015 | 0.51 | 0.51 |

788

789

790	Cytotoxicity of Extracts and Fraction against 3T3 (Mouse Fibroblast)

791	**and BJ (Human Fibroblast) Cell Lines.**

|  |  | **~~3T3~~** ~~(mouse~~ ~~fibroblast)~~ | **~~BJ~~** ~~(human~~ ~~fibroblast~~**~~)~~** |
| --- | --- | --- | --- |
|  |  | **~~IC~~50 ~~(~~*~~μ~~*~~g/ml ± SD)~~** | **~~IC~~50 ~~(~~*~~μ~~*~~g/ml ± SD)~~** |
| **~~Extracts~~** | ~~Ethanol/water~~ | ~~>100~~ | ~~>100~~ |
|  | ~~Pet ether~~ | ~~>100~~ | ~~>100~~ |
|  | ~~Dichloromethane~~ | ~~4.5 ± 0.5~~ | ~~26.5±1.1~~ |
|  | ~~Ethyl acetae~~ | ~~>100~~ | ~~>100~~ |
| **~~Fractions~~** | ~~A~~ | ~~6.67 ± 0.112.2 ± 1.5~~ | ~~15.2 ± 1.0~~ |
|  | ~~B~~ | ~~4.8 ± 1.69.95 ± 1.9~~ | ~~6.06 ± 0.4~~ |
|  | ~~C~~ | ~~2.7 ± 0.36.30 ±~~  ~~0.018~~ | ~~9.8 ± 0.4~~ |
|  | ~~D~~ | ~~3.3 ± 0.36.37 ±~~  ~~0.015~~ | ~~23.8 ± 0.9~~ |
| **~~Standard drugs~~** | ~~Doxorubicin~~ | ~~0.1 ± 0.02~~ | ~~0.1± 0.02~~ |

792

793

794

795	~~Table 6: Cytotoxicity of Compounds 1-9 against 3T3 (Mouse~~

796	**~~Fibroblast) and, BJ (Human Fibroblast) Cell Lines.~~**

797

|  |  | **~~3T3~~** ~~(mouse~~ ~~fibroblast)~~ | **~~BJ~~** ~~(human~~ ~~fibroblast~~**~~)~~** |
| --- | --- | --- | --- |
|  | **~~Plant extracts~~** | **~~IC~~50 ~~(~~*~~μ~~*~~M ± SD)~~** | **~~IC~~50 ~~(~~*~~μ~~*~~M± SD)~~** |
| **~~Pure Compounds~~** | **1** | ~~Non cytotoxic~~ | ~~Non cytotoxic~~ |
|  | **2** | ~~10 ± 0.4~~ | ~~27.6 ± 0.9~~ |
|  | **3** | ~~0.72 ± 0.5~~ | ~~10.8 ± 1.5~~ |
|  | **4** | ~~19.6± 0.8~~ | ~~˂50~~ |
|  | **5** | ~~0.4 ± 0.07~~ | ~~14.2 ± 0.8~~ |
|  | **6** | ~~0.6 ± 0.04~~ | ~~10.4 ± 1.0~~ |
|  | **7** | ~~Non cytotoxic~~ | ~~Non cytotoxic~~ |
|  | **8** | ~~Non cytotoxic~~ | ~~Non cytotoxic~~ |
|  | **9** | ~~Non cytotoxic~~ | ~~Non cytotoxic~~ |

| **~~Standard drugs~~** | ~~Doxorubicin~~ | ~~0.1 ± 0.02~~ | ~~0.1± 0.02~~ |
| --- | --- | --- | --- |
|  |  |  | --- |

798

799
