# Supplementary material for: Anti-leishmanial physalins—Phytochemical investigation, in vitro evaluation against clinical and MIL-resistant L. tropica strains and in silico studies
Source: PLoS One. 2022 Nov 28;17(11):e0274543. doi: 10.1371/journal.pone.0274543 (PMC9704608; doi:10.1371/journal.pone.0274543)
Supplement: Attachment — Submitted filename: Response to reviewers.docx [file pone.0274543.s030.docx]

**Comments from Reviewer**

**[PONE-D-22-16415R1] - [EMID: d6921b01d1c73774]**

| **S. No** | **Comment** | **Response** |
| --- | --- | --- |
| **1.** | The title still needs corrections as I displayed by track changes. | The correction has done accordingly |
| **2.** | In the abstract, the authors abbreviated cutaneous leishmaniasis in two places (L 27-30) | In the whole manuscript after first appearance of Cutaneous Leishmaniasis the rest has changed to CL except the first line of introduction as its looks odd to start the first line with an abbreviation. |
| **3.** | The authors did not use an MTT assay to check the viability of promastigotes. Did they check for viability before performing the treatments to make sure they are all viable (motile) because they counted them by eyes? | For the evaluation of parasite life cycle and morphology the reference standard protocol of wet mount method was used. Cell viability was initially evaluated by wet mount method by observing the live cells under compound microscope. Parasite growth was assessed by counting live / motile promastigotes in a Neubauer chamber. Viable cell count was used for the further experimentations.  Whereas, the MTT assay was used for the promastigote growth and inhibition assays.  The details have been elaborated now in experimental section on Page- 10, line 224-246. |
| **4.** | The authors visually counted the promastigote stage, why did they use DAPI stain? How did it support the study? | DAPI staining was not used for the counting of promastigotes, however, it was used for the evaluation of any possible morphological and other alterations including nuclear fragmentation and apoptosis in *lesihmania* parasite caused by acquisition of high concentration of miltefosine. The purpose of use of DAPI is now well explained in revised manuscript page 19, line 444-449. |
| **5.** | Mention why you docked with phosphoglucose isomerase. | Compounds were docked against various metabolically important enzymes. Among them, they showed better binding affinities against phosphoglucose isomerase and myrositoyltransferase. (Mentioned in revised manuscript, lines 465-466).  Phosphoglucose isomerase (PGI) is considered a promising target for the development of anti-parasitic drugs, as it acts on two essential metabolic pathways, glycolysis and gluconeogenesis. PGI is an aldose-ketose isomerase that catalyzes the reversible interconversion of G6P into fructose-6-phosphate (F6P) (Mentioned in revised manuscript, lines 537-541). |
| **6** | Why the authors did not use a uniform and standard positive control like AmpB throughout the experiments so that one could be able to compare the outcome? All of the positive controls even AmpB, pentamidine, and MIL are the second-line drugs, except AmBisome (a liposomal form of AmpB is used to treat Indian and Mediterranean type kala-azar (visceral leishmaniasis due to *L.donovani* and *L.infantum*, respectively). The authors could use sodium stibogluconate (Pentostam®) or meglumine antimoniate (Glucantime®) and if resistance is a common phenomenon then AmpB would be OK, otherwise using different positive controls is not realistic. Miltefosine does not affect *L. tropica* it is only active at around 83%-85% against *L. major* and *L. infantum*. | One of the recommended treatment regimens for leishmaniasis includes the combination of amphotericin B and miltefosine. Combination therapies are effective and safe, and decrease the duration of therapy. Based on above statement in the presented study Amphotericin B, pentamidine and miltefosine were used as a positive control to broaden the range against commercially available drugs. The statement has elaborated in revised manuscript page 20, line 483-489. |
| **7** | AmpB is not the drug of choice for *L.tropica* and *L. major*. It is rather a liposomal form OF AmpB (AmBisome used against VL). It is all right you used other second-choice drugs because you had no access to meglumine antimonite (Glucantime®) or sodium stibogluconate(Pentostam®), but you should have used similar positive controls to be able to compare the activity of each component. | The one of the mechanisms of action of miltefosine is the inhibition and alteration in mitochondrial functions which is similar to the Sodium stibogluconate which inhibit the formation of ATP and GTP. The similarity pushed us to use miltefosine. |
| **8** | How could you tell that the difference between the test results and the untreated control group is significant, or how could you predict the significant difference among groups?  The author should mention the statistical test and define the P-value. In the results whenever a significant level is seen the p-value should be pointed out. | A one-way ANOVA was used to analyze statistically differences at a *P-value* of 0.05 (95% confidence interval) in conjugation with the Dunnett test using Graph pad prism version 9.4.1.  The statistical analysis test method has now been incorporated in revised manuscript page 13, line 307-309.  The *P-values* has also been added in the revised manuscript’s result and discussion (pages 17 and 18, lines 405-418) and in the tables 2 and 3. |
| **9.** | The docking technique is employed to predict the tentative binding affinity of the ligand-receptor complex ahead of time. So this should preferably be presented at the beginning of the M+M | The molecular docking studies were conducted for the identified anti-leishmanial physalins on the basis of results of anti-leishmanial activities. We therefore arrange the experimental in a step-by-step way we did i.e. Phytochemical investigation and structure elucidation, anti-leishmanial activity evaluation and finally the docking studies. |
| **10.** | After using Leishmania tropica or L.major the genus should be used in form of abbreviation throughout the manuscript (L.tropica or L.major). Mixed using of the species wording is used and this should be corrected consistently. | The mixing has been corrected accordingly. |
| **11** | The Old and New World should be written with capital first initials (like World). | The correction has been made. |
| **12** | The scientific name of organisms either parasites or sand flies should be printed in the capital font ( e.i., Lutzomyia). In some places, Leishmania is not italic or with a small l. | The word “*Leishmania*” is written with “L” and italic now throughout the revised manuscript |
| **13** | Subtitles are not uniformly written and they are written with mixed capital and small ones, whereas the journal style is different. I advise the authors once again to review the journal’s instructions or one of the newly published Plos One articles and follow through and make the corrections. | The subtitles and rest of the manuscript has now been carefully formatted as per format of Plos One. |
